# Supplementary material for: TNF-α-mediated m6A modification of ELMO1 triggers directional migration of mesenchymal stem cell in ankylosing spondylitis
Source: Nat Commun. 2021 Sep 10;12:5373. doi: 10.1038/s41467-021-25710-4 (PMC8433149; doi:10.1038/s41467-021-25710-4)
Supplement: Supplementary file 1 — Supplementary Information File [file 41467_2021_25710_MOESM1_ESM.pdf]

Supplementary Information File

TNF-α-mediated m^{6}A modification of ELMO1 triggers directional migration of mesenchymal stem cell in ankylosing spondylitis

Zhongyu Xie[#], Wenhui Yu[#], Guan Zheng[#], Jinteng Li, Shuizhong Cen, Guiwen Ye, Zhaofeng Li, Wenjie Liu, Ming Li, Jiajie Lin, Zepeng Su, Yunshu Che, Feng Ye, Peng Wang[*], Yanfeng Wu[*], Huiyong Shen[*]

Supplementary Figure 1

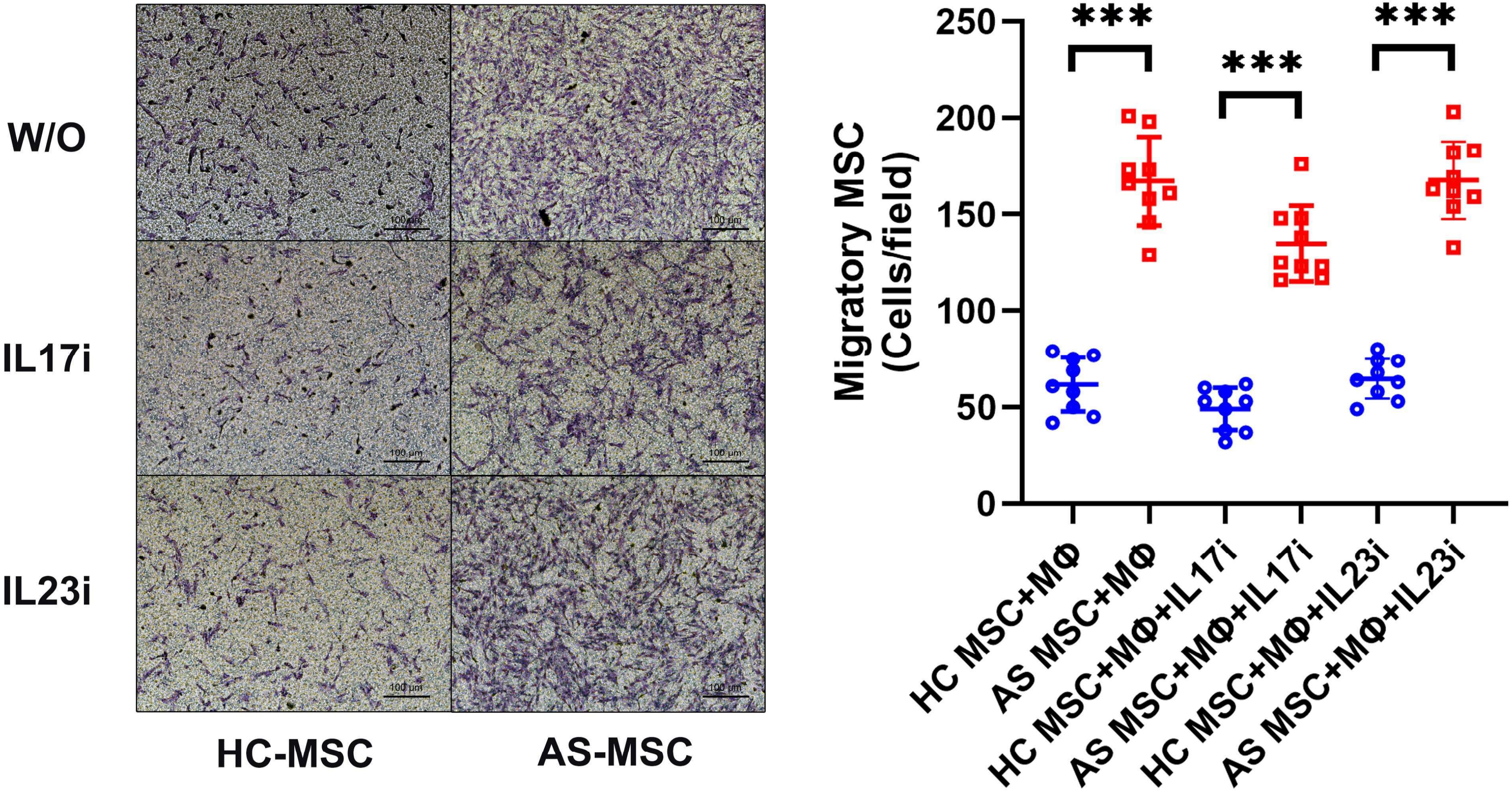

Supplementary Figure 1 Migration of HC-MSC and AS-MSC with IL17 and IL23 neutralizing antibody

The number of stained migratory AS-MSC (n=9) was much more than that of HC-MSC (n=9) with the IL17 (IL17i; P=4.171E-9) or IL23 (IL23i; 2.9817E-10) neutralizing antibodies. Data were analyzed using two-tailed Student's *t*-test. Values are presented as the mean ± SD. *** indicates P<0.001.

Supplementary Figure 2

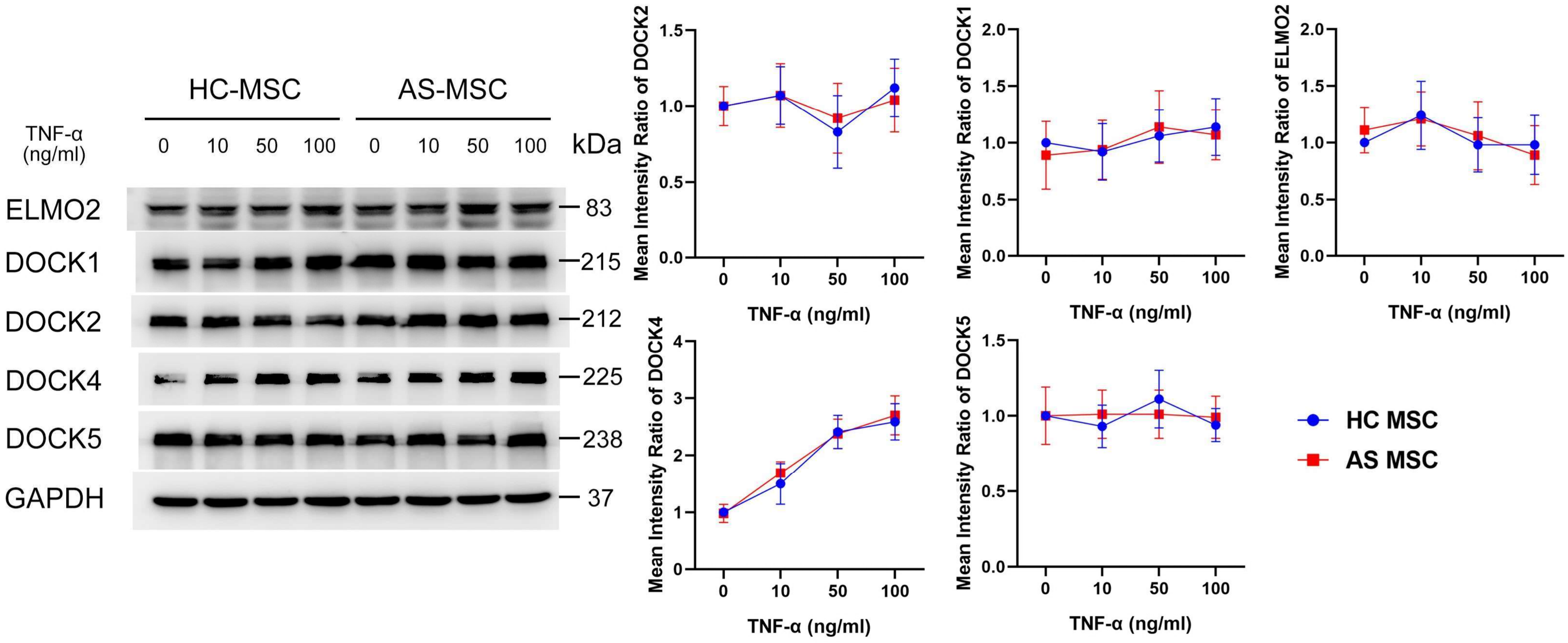

Supplementary Figure 2 Bioinformatic analysis of RNA sequencing data

(a) The volcano figures of RNA sequencing. **(b)** The GO analysis including biological process term, molecular function term and cellular component of RNA sequencing. **(b)** The signal pathway figure in KEGG datasets of chemokine signaling pathway.

Supplementary Figure 3

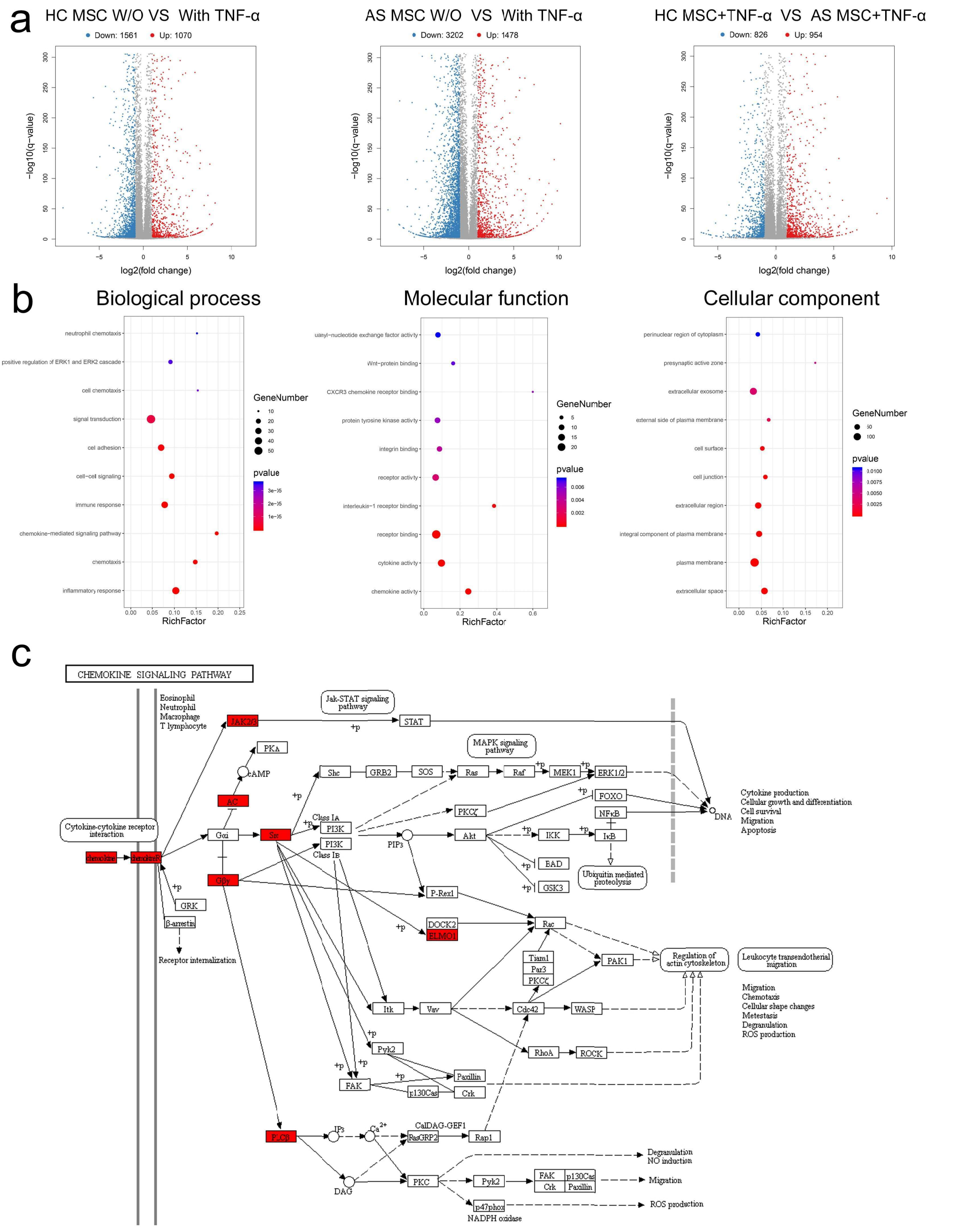

Supplementary Figure 3 Expression of ELMOs and DOCKs in TNF-α treat HC-MSC and AS-MSC

With treated with TNF-α at a concentration of 0 to 100 ng/ml, the expression levels of ELMO2, DOCK1, DOCK2, DOCK4 and DOCK5 of AS-MSC (n=9) were equal to those of HC-MSC (n=9). Values are presented as the mean ± SD.

Supplementary Figure 4

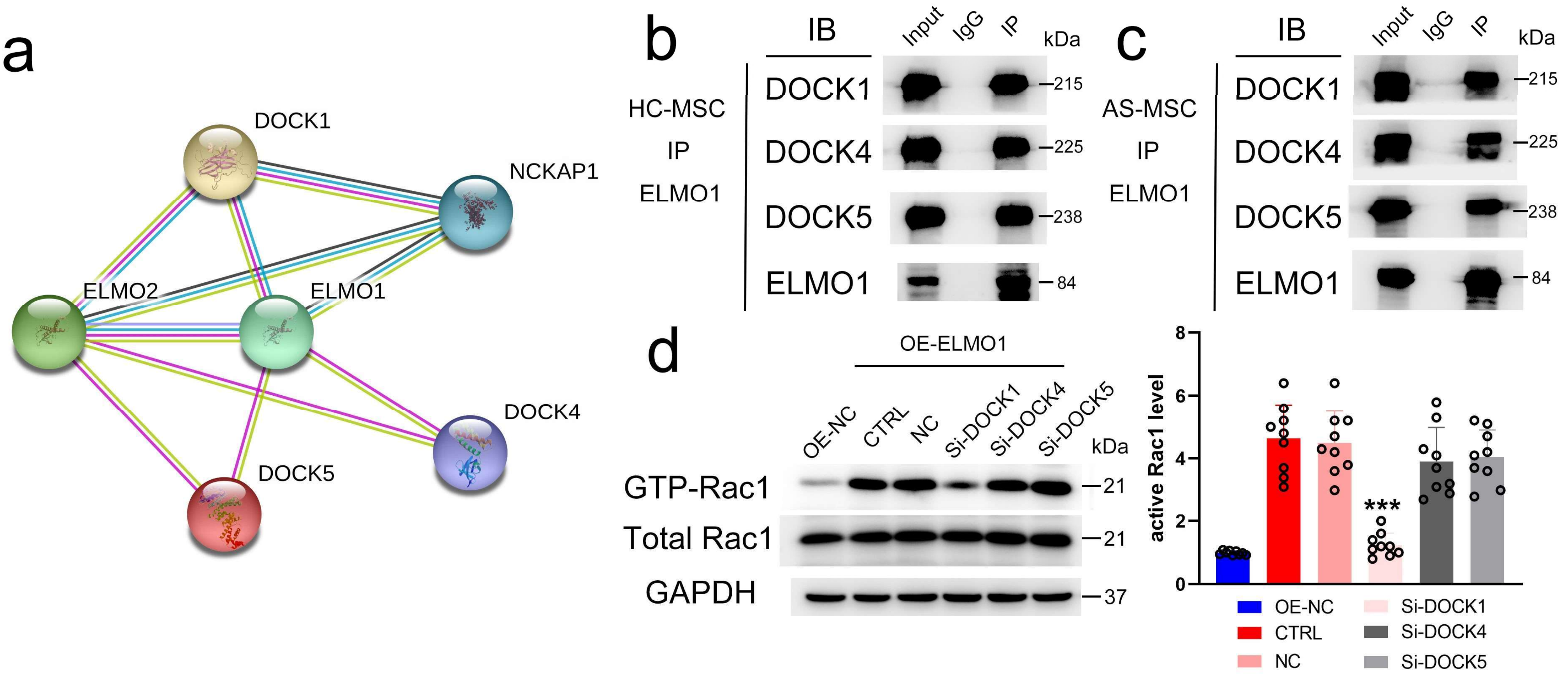

Supplementary Figure 4 Protein-Protein interaction of ELMO1 in TNF-α treated MSC

(a) Results of Co-IP and LC-MS/MS showed that ELMO1 could bind to ELMO1, DOCK1, DOCK4, DOCK5 and NCKAP1 in MSC. **(b & c)** Co-IP and western blotting confirmed that ELMO1 could bind to DOCK1, 4 and 5 in both TNF-α treated HC-MSC and AS-MSC. Experiment was repeated three times independently. **(d)** With TNF-α treatment, the Rac1 active level of MSC was much higher in the OE-ELMO1 group (n=9), but was inhibited after siRNA-DOCK1 transfection (n=9; P=5.02E-10) rather than siRNA-DOCK4 (n=9) or siRNA-DOCK5 (n=9). Data were analyzed using one-way ANOVA followed by Bonferroni's post hoc comparisons. Values are presented as the mean ± SD. *** indicates P<0.001.

Supplementary Figure 5

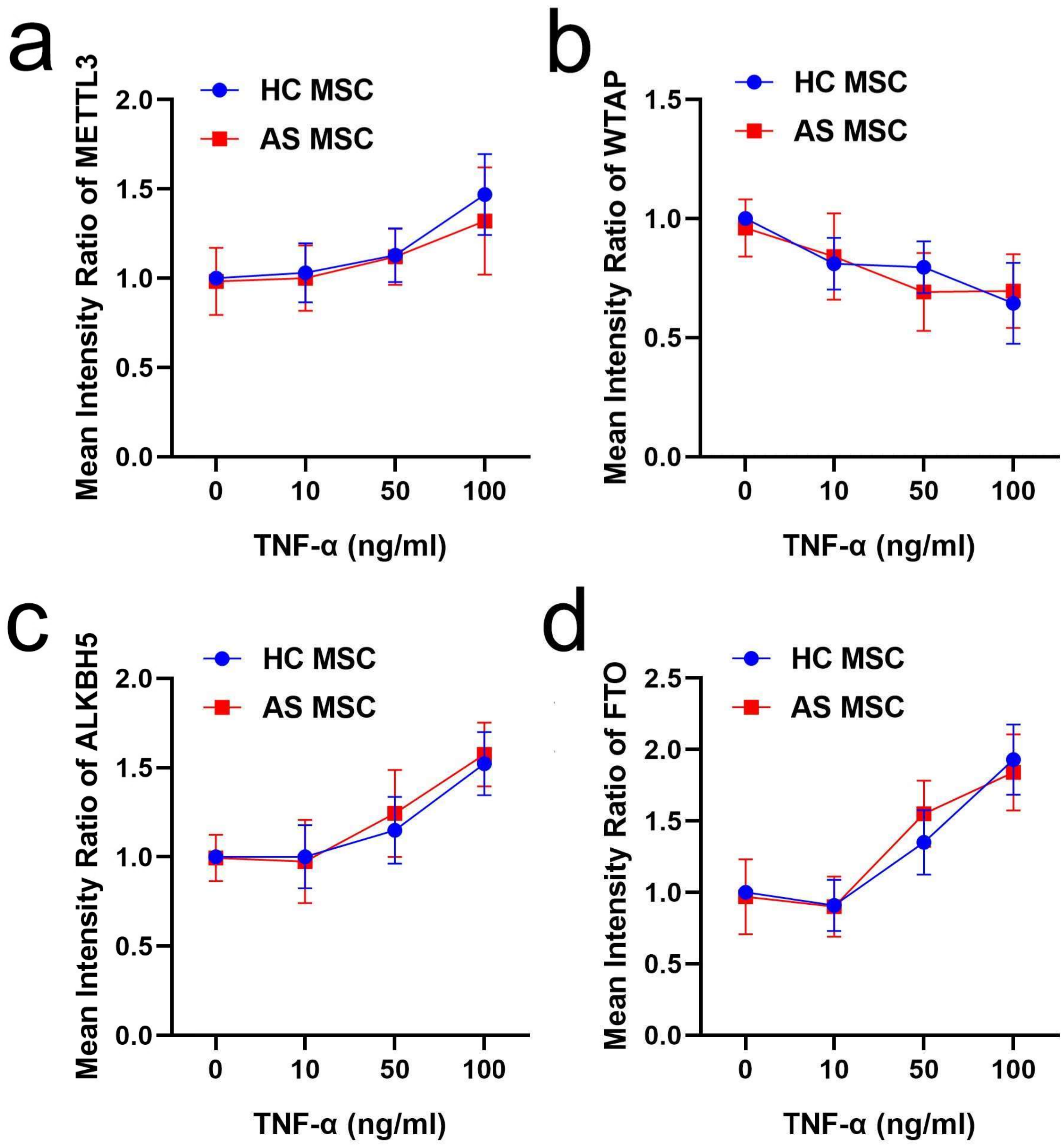

Supplementary Figure 5 METTL3, WTAP, FTO and ALKBH5 expression in TNF-α treated HC-MSC and AS-MSC.

(a-d) WTAP expression was decreased, and METTL3, FTO and ALKBH5 expression was increased in MSC after TNF-α treatment. No differences in METTL3, WTAP, FTO and ALKBH5 expression were shown between TNF-α treated HC-MSC (n=9) and AS-MSC (n=9). Values are presented as the mean ± SD.

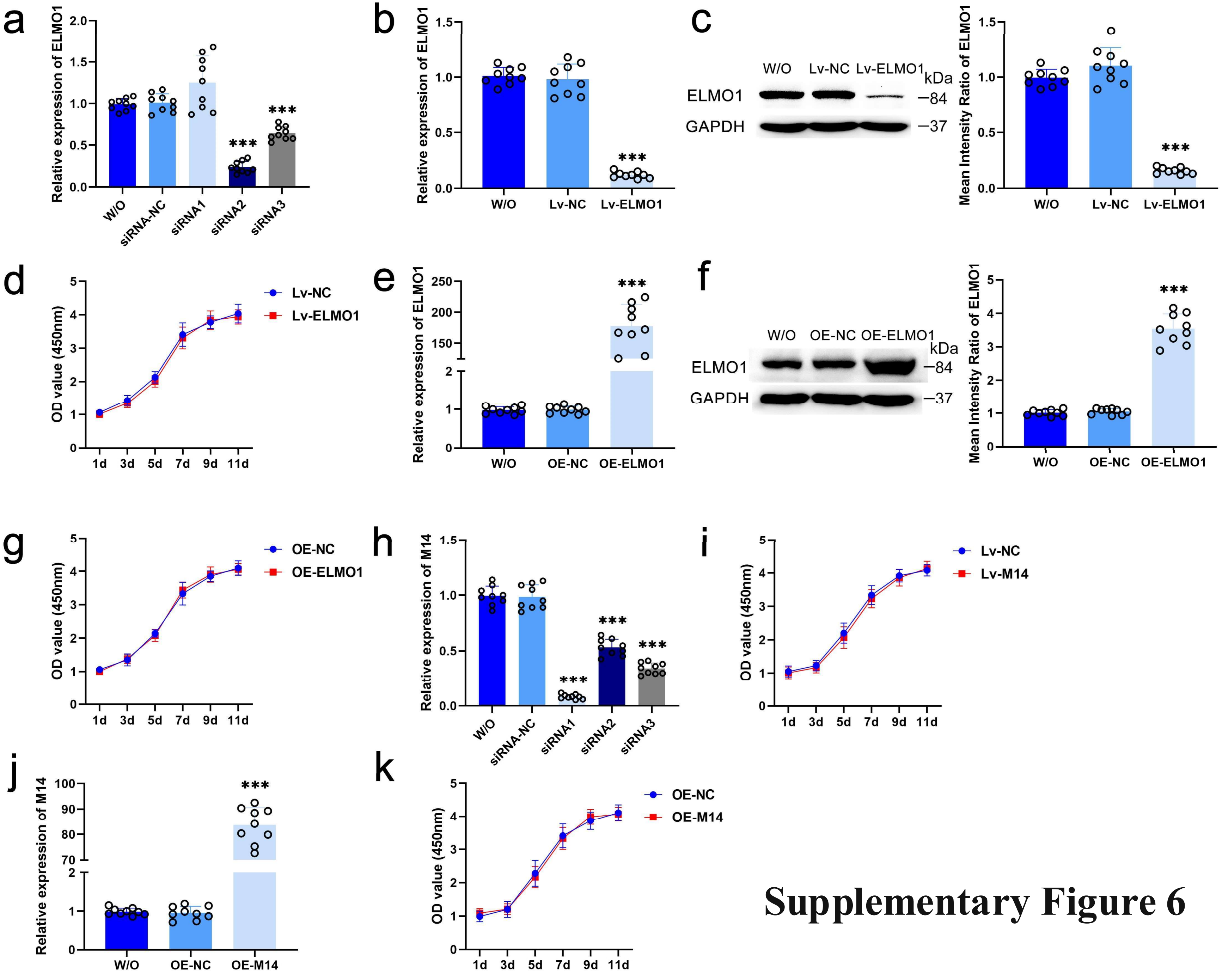

Supplementary Figure 6

Supplementary Figure 6 Efficiency of lentiviruses and siRNAs

(a) The *ELMO1* expression in MSC (n=9) was significantly lower by siRNA2 (n=9; P=1.2458E-11) and siRNA3 groups (n=9; P=0.000209). **(b)** The *ELMO1* expression in gene level was markedly lower in Lv-ELMO1 group (n=9) than Lv-NC group (n=9; P=7.4424E-16). **(c)** The ELMO1 expression in protein level was markedly lower in Lv-ELMO1 group (n=9) than Lv-NC group (n=9; P=1.4056E-15). (d) The OD values of CCK-8 assay were equal between the Lv-NC group (n=9) and Lv-ELMO1 group (n=9) from day 1 to 11. **(e)** The *ELMO1* expression in gene level was markedly higher in OE-ELMO1 group (n=9) than OE-NC group (n=9; P=3.8788E-15). **(f)** The ELMO1 expression in protein level was markedly higher in OE-ELMO1 group (n=9) than OE-NC group (n=9; P=5.6136E-16). (g) The OD values of CCK-8 assay were equal between the OE-NC group (n=9) and OE-ELMO1 group (n=9) from day 1 to 11. **(h)** The METTL14 expression was inhibited by siRNA1, siRNA2 and siRNA3, and the siRNA1 had the highest inhibitory efficiency (n=9 biologically independent cells; P=1.7222E-25). **(i)** The OD values of CCK-8 assay were equal between the Lv-NC group (n=9) and Lv-M14 group (n=9) from day 1 to 11. **(j)** The *METTL14* expression in gene level was markedly higher in OE-ELMO1 group (n=9) than OE-NC group (n=9; P=8.0527E-24). **(k)** The OD values of CCK-8 assay were equal between the OE-NC group (n=9) and OE-M14 group (n=9) from day 1 to 11. Data were analyzed using one-way ANOVA followed by Bonferroni's post hoc comparisons. Values were present as means ± SD. *** indicates $P<0.001$. Lv-NC or OE-NC indicates MSC transfected with control lentiviruses. Lv-ELMO1 indicates MSC transfected with lentiviruses encoding

an shRNA specific for ELMO1. OE-ELMO1 indicates MSC transfected with lentiviruses overexpressing ELMO1.

Supplementary Figure 7

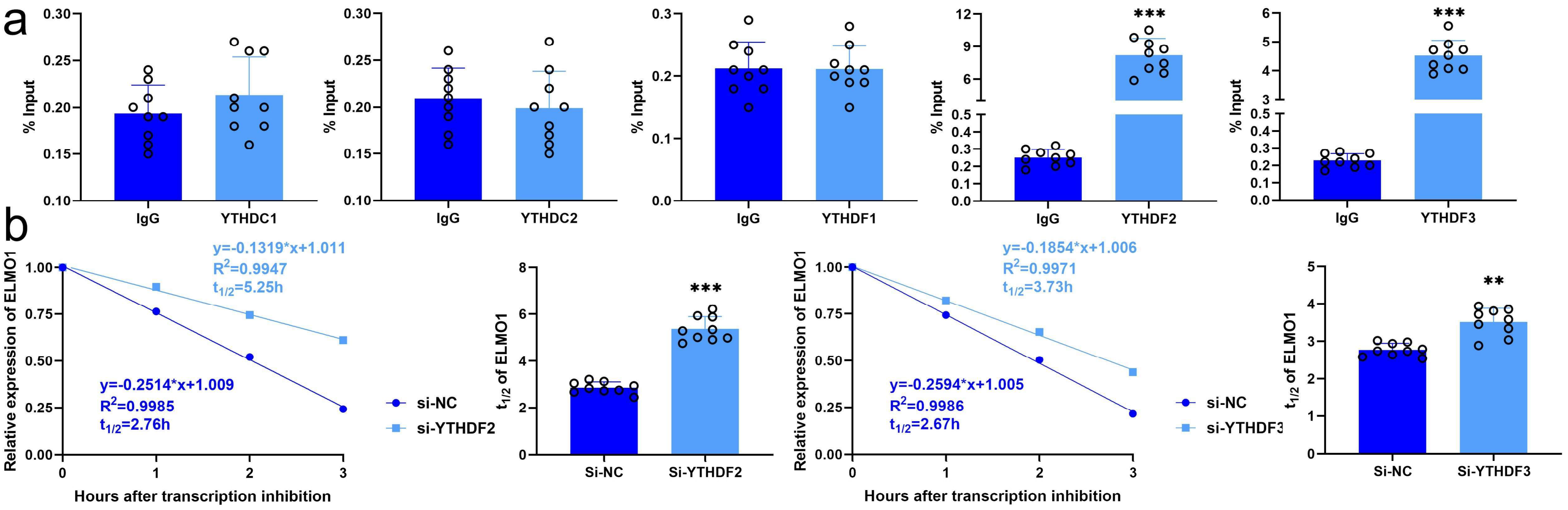

Supplementary Figure 7 YTHDF2 and YTHDF3 contribute to *ELMO1* mRNA degeneration in MSC

(a) The %Input levels of the YTHDC1, YTHDC2 and YTHDF1 groups were equal to those of the IgG group (n=9). The %Input levels of the YTHDF2 (n=9; P=6.8265E-11) and YTHDF3 (n=9; P=4.6463E-14) groups were higher than those of the IgG group (n=9). **(b)** The degeneration rates of *ELMO1* mRNA in the si-YTHDF2 (n=9; P=5.6429E-10) and si-YTHDF3 (n=9; P=0.000052) groups were both lower compared to the si-NC groups (n=9). Data were analyzed using two-tailed Student's *t*-test. Values are presented as the mean ± SD. *** indicates P<0.001.

Supplementary Table 1 Characteristics of the study subjects

	**Healthy controls**	**AS patients**
Number	15	15
Age, year	29.1±5.4	32.0±10.8
No.(%) male	11(73%)	11(73%)
HLA-B27 positive no.(%)	0	15(100%)*
Disease duration, year	NA	8.5±6.0
CRP, mg/L	3.0±0.7	25.4±7.2*
ESR, mm/h	6.9±2.4	38.2±14.9*
BASDAI	0.52±0.28	4.06±1.27*

Mean±SD. AS, ankylosing spondylitis; HLA-B27, human leukocyte antigen B27; CRP, C-reactive protein; ESR, erythrocyte sedimentation rate; BASDAI, the bath ankylosing spondylitis disease activity index; Data were analyzed using two-tailed Student's *t*-test or Chi-square test; * indicates $P < 0.05$ compared to healthy controls.

Supplementary Table 2 Primers for qRT-PCR

Gene	Forward primer (5`-3`)	Reverse primer (5`-3`)
GAPDH	GGAGCGAGATCCCTCCAAAAT	GGCTGTTGTCATACTTCTCATGG
ELMO1	TATTGTCGGCTTTCGCCAAAT	TCAAGCACCTCCTTGTTTTGT
METTL14	GAACACAGAGCTTAAATCCCCA	TGTCAGCTAAACCTACATCCCTG

Supplementary Table 3 siRNA sequence

Target	siRNA	Sequence
Control	siRNA-NC	GCACTAAGGAGTTCGTCTTTG
	siRNA-1	AACATTTTGGGGACTTACAAT
ELMO1	siRNA-2	CCCCAAACTCATGGAAATTGA
	siRNA-3	GCCGATAGTTCAAACTTCTAT
	siRNA-1	AAGAAAGGTTGGATAAGAGTT
METTL14	siRNA-2	GACAAGTACTCGGGATAGTGA
	siRNA-3	CAGAGAGAAATTGCTGAAACA
	siRNA-1	GCACATGATCTATGACCTTAT
DOCK1	siRNA-2	GCTGGTGATGAAGCGATTTCA
	siRNA-3	GCACGATCTTATCGTCTATAA
	siRNA-1	GCTCCCTCTTTGTGGATTTGG
DOCK4	siRNA-2	GCAGTTTCCTTACAGCTATTG
	siRNA-3	GCTGCAAGATAGCAAATTTCA
	siRNA-1	GCCTCTATGTGAACTTCAAGA
DOCK5	siRNA-2	GCCTTTGGGTATCCTTGAAGC
	siRNA-3	GCTGGATATGAAGGCATTTCA
	siRNA-1	GCTCCTGGCATGAATACTATA
YTHDF2	siRNA-2	GCAGTGGGTTCGGTCATAATG
	siRNA-3	GGACGTTCCCAATAGCCAACT

	siRNA-1	GGATCTCAGGGACAATCAACA
YTHDF3	siRNA-2	GCTGGATTTGGCAATGATACT
	siRNA-3	GGATTTGGCAATGATACTTTG

Supplementary Table 4 Characteristics of the study subjects for enthesis immunofluorescence

	**AS patients**	**non-AS patients**
Number	9	9
Age, year	29.2±4.6	31.0±6.3
No.(%) male	4(66.7%)	4(66.7%)
HLA-B27 positive no.(%)	6(100%)	0
Disease duration, year	6.2±1.9	7.1±3.0
CRP, mg/L	21.9±11.2	3.2±0.8
ESR, mm/h	57.7±18.3	5.5±1.8
BASDAI	4.37±1.01	1.28±0.44

Mean±SD. AS, ankylosing spondylitis; HLA-B27, human leukocyte antigen B27; CRP, C-reactive protein; ESR, erythrocyte sedimentation rate; BASDAI, the bath ankylosing spondylitis disease activity index. Non-AS patients were patients with lumbar intervertebral disc herniation.

Supplementary Table 5 LC-MS/MS results of the ELMO1 group

Accession	Description	Exp. q-value	Sum PEP Score	Coverage (%)	Unique Peptides	AAs	MW (kDa)	calc. pI	Score
P35579-1	Myosin-9	0	417.172	52	78	1960	226.4	5.6	570.43
Q92556-1	Engulfment and cell motility protein 1	0	388.718	71	43	727	83.8	6.28	627.69
P60709	Actin, cytoplasmic 1	0	223.801	65	9	375	41.7	5.48	346.85
P14618	Pyruvate kinase PKM	0	212.654	65	31	531	57.9	7.84	292.01
P04406-1	glyceraldehyde-3-phosphate dehydrogenase	0	211.255	73	19	335	36	8.46	319.68
P35580	Myosin-10	0	193.313	29	34	1976	228.9	5.54	239.34
P08238	Heat shock protein HSP 90-beta	0	192.696	55	20	724	83.2	5.03	275.08
P04264	Keratin, type II cytoskeletal 1	0	183.041	61	33	644	66	8.12	306.14
P07437	tubulin beta chain	0	171.821	67	4	444	49.6	4.89	246.57

P07900	Heat shock protein HSP 90-alpha	0	169.878	46	18	732	84.6	5.02	249.53
P68371	Tubulin beta-4B chain	0	167.08	67	1	445	49.8	4.89	242.37
P38646	Stress-70 protein, mitochondrial	0	144.808	53	33	679	73.6	6.16	211.3
P68363	Tubulin alpha-1B chain	0	139.802	60	3	451	50.1	5.06	219.08
P68104	Elongation factor 1-alpha 1	0	136.372	57	8	462	50.1	9.01	238.2
P35527	Keratin, type I cytoskeletal 9	0	133.163	51	26	623	62	5.24	193.39
P04350	Tubulin beta-4A chain	0	130.26	55	1	444	49.6	4.88	180.94
P35908	Keratin, type II cytoskeletal 2 epidermal	0	129.511	56	23	639	65.4	8	191.55
P11142-1	Heat shock cognate 71 kDa protein	0	120.21	42	21	646	70.9	5.52	182.13
Q9BQE3	Tubulin alpha-1C chain	0	115.556	60	3	449	49.9	5.1	178.06
Q86YZ3	Hornerin	0	113.715	24	18	2850	282.2	10.04	114.29
P60842	Eukaryotic initiation factor 4A-I	0	112.155	46	9	406	46.1	5.48	133.78
Q13885	Tubulin beta-2A chain	0	106.985	47	1	445	49.9	4.89	184.27

Q9BVA1	Tubulin beta-2B chain	0	106.971	47	1	445	49.9	4.89	184.36
P13645	Keratin, type I cytoskeletal 10	0	106.871	42	21	584	58.8	5.21	191.43
P04075	fructose-bisphosphate aldolase A	0	106.721	75	18	364	39.4	8.09	170.21
P0DMV8	heat shock 70 kDa protein 1A	0	104.385	43	22	641	70	5.66	161.59
P13639	Elongation factor 2	0	103.356	38	28	858	95.3	6.83	146.65
P10809	60 kDa heat shock protein, mitochondrial	0	95.543	43	23	573	61	5.87	152.39
P49327	Fatty acid synthase	0	85.639	12	20	2511	273.3	6.44	102.08
Q14185	Dedicator of cytokinesis protein 1	0	82.515	16	23	1865	215.2	7.56	101.47
P11021	78 kDa glucose-regulated protein	0	79.854	38	20	654	72.3	5.16	108.53
P68133	Actin, alpha skeletal muscle	0	78.707	30	1	377	42	5.39	152.82
Q13509	tubulin beta-3 chain	0	78.032	33	1	450	50.4	4.93	111.71
Q00610-1	Clathrin heavy chain 1	0	75.725	15	18	1675	191.5	5.69	92.43

P06576	ATP synthase subunit beta, mitochondrial	0	72.357	36	12	529	56.5	5.4	69.84
P78371-1	T-complex protein 1 subunit beta	0	72.088	39	18	535	57.5	6.46	101.42
P07195	L-lactate dehydrogenase B chain	0	68.776	39	12	334	36.6	6.05	111.67
P49368-1	T-complex protein 1 subunit gamma	0	66.741	40	17	545	60.5	6.49	90.24
P12277	Creatine kinase B-type	0	66.258	42	12	381	42.6	5.59	99.99
P23396-1	40S ribosomal protein S3	0	65.978	70	16	243	26.7	9.66	94.64
P50990	T-complex protein 1 subunit theta	0	65.843	38	19	548	59.6	5.6	100.95
P06733-1	alpha-enolase	0	65.759	47	16	434	47.1	7.39	93.84
P26641	elongation factor 1-gamma	0	64.382	33	16	437	50.1	6.67	101.37
P52272	Heterogeneous nuclear ribonucleoprotein M	0	64.097	25	17	730	77.5	8.7	93.77
P31943	Heterogeneous nuclear	0	61.823	40	11	449	49.2	6.3	80.16

	ribonucleoprotein H								
P25705-1	ATP synthase subunit alpha, mitochondrial	0	61.45	31	15	553	59.7	9.13	89.71
P00338-1	L-lactate dehydrogenase A chain	0	57.325	40	13	332	36.7	8.27	82.38
P61978	Heterogeneous nuclear ribonucleoprotein K	0	57.173	33	13	463	50.9	5.54	95.86
P11586	C-1-tetrahydrofolate synthase, cytoplasmic	0	57.138	22	16	935	101.5	7.3	76.56
P05023	Sodium/potassium-transporting ATPase subunit alpha-1	0	57.096	18	14	1023	112.8	5.49	76.95
P12956	X-ray repair cross-complementing protein 6	0	56.962	25	12	609	69.8	6.64	67.04
Q00839	Heterogeneous nuclear	0	56.088	24	18	825	90.5	6	75.55

	ribonucleoprotein U								
P13797	Plastin-3	0	55.636	28	13	630	70.8	5.6	62.95
P78527	DNA-dependent protein kinase catalytic subunit	0	55.397	6	22	4128	468.8	7.12	68.86
P14625	Endoplasmin	0	55.204	29	19	803	92.4	4.84	74.16
P22314	Ubiquitin-like modifier-activating enzyme 1	0	53.923	17	13	1058	117.8	5.76	66.05
P36578	60S ribosomal protein L4	0	52.741	33	14	427	47.7	11.06	74.71
P09651-1	Heterogeneous nuclear ribonucleoprotein A1	0	52.411	39	9	372	38.7	9.13	84.26
Q05639	Elongation factor 1-alpha 2	0	52.373	30	1	463	50.4	9.03	103.1
P04843	Dolichyl-diphosphooligosaccharide--protein glycosyltransferase subunit 1	0	52.06	25	11	607	68.5	6.38	63.66

Q99623	Prohibitin-2	0	51.003	46	12	299	33.3	9.83	83.36
P00558	phosphoglycerate kinase 1	0	48.834	43	16	417	44.6	8.1	68.57
P08670	Vimentin	0	47.601	44	15	466	53.6	5.12	74.12
P11940-1	Polyadenylate-binding protein 1	0	47.515	23	11	636	70.6	9.5	58.2
P50991	T-complex protein 1 subunit delta	0	47.278	34	14	539	57.9	7.83	65.72
O43175	D-3-phosphoglycerate dehydrogenase	0	47.202	30	13	533	56.6	6.71	71.86
P63244	Receptor of activated protein C kinase 1	0	47.06	34	9	317	35.1	7.69	72.29
Q15366-3	Isoform 3 of Poly(rC)-binding protein 2	0	46.72	46	8	362	38.2	6.79	61.97
P09874	Poly [ADP-ribose] polymerase 1	0	45.877	19	15	1014	113	8.88	56.67
Q4VCS5	Angiomotin	0	45.805	17	14	1084	118	7.64	70.92
P01834	immunoglobulin kappa constant	0	45.248	86	6	107	11.8	6.52	62.39
P19338	Nucleolin	0	44.443	23	17	710	76.6	4.7	69.84
P05141	ADP/ATP translocase 2	0	44.219	45	6	298	32.8	9.69	73.03

P06748	Nucleophosmin	0	43.727	37	11	294	32.6	4.78	59.74
P14868	Aspartate--tRNA ligase, cytoplasmic	0	43.705	32	13	501	57.1	6.55	43.36
P40227-1	T-complex protein 1 subunit zeta	0	42.824	25	15	531	58	6.68	77.45
P21796	voltage-dependent anion-selective channel protein 1	0	42.74	49	8	283	30.8	8.54	53.29
P18621	60S ribosomal protein L17	0	42.732	46	8	184	21.4	10.17	58.24
P62424	60S ribosomal protein L7a	0	42.492	40	11	266	30	10.61	67.13
P23528	Cofilin-1	0	42.378	63	8	166	18.5	8.09	66.42
P62937	peptidyl-prolyl cis-trans isomerase A	0	41.06	67	11	165	18	7.81	69.61
P45880	Voltage-dependent anion-selective channel protein 2	0	41.053	41	8	294	31.5	7.56	58.87
P30050-1	60S ribosomal protein L12	0	40.945	64	8	165	17.8	9.42	60.88
Q06830	peroxiredoxin-1	0	40.758	55	7	199	22.1	8.13	61.06

P61247	40S ribosomal protein S3a	0	40.692	52	13	264	29.9	9.73	64.59
P22234	multifunctional protein ADE2	0	40.55	26	10	425	47	7.23	54.91
Q13263	Transcription intermediary factor 1-beta	0	40.118	24	14	835	88.5	5.77	50.18
P60891	ribose-phosphate pyrophosphokinase 1	0	39.862	32	4	318	34.8	6.98	54.9
P12268	inosine-5'-monophosphate dehydrogenase 2	0	38.756	28	10	514	55.8	6.9	52.61
Q02790	Peptidyl-prolyl cis-trans isomerase FKBP4	0	38.449	28	9	459	51.8	5.43	53
P35232	Prohibitin	0	38.131	44	11	272	29.8	5.76	59.14
P22626	heterogeneous nuclear ribonucleoproteins A2/B1	0	38.121	33	10	353	37.4	8.95	57.52
P62701	40S ribosomal protein S4, X isoform	0	37.314	40	12	263	29.6	10.15	66.37
P49411	elongation factor Tu, mitochondrial	0	37.299	25	9	452	49.5	7.61	46.15

P27824	Calnexin	0	37.155	14	8	592	67.5	4.6	49.79
P54577	Tyrosine--tRNA ligase, cytoplasmic	0	37.006	29	14	528	59.1	7.05	39.06
P17987	T-complex protein 1 subunit alpha	0	37.001	22	10	556	60.3	6.11	54.83
P55060-1	Exportin-2	0	36.955	12	10	971	110.3	5.77	44.7
Q14240-1	eukaryotic initiation factor 4A-II	0	36.503	23	1	407	46.4	5.48	32.31
Q02878	60S ribosomal protein L6	0	36.397	37	13	288	32.7	10.58	58.71
P13647	keratin, type II cytoskeletal 5	0	36.356	20	6	590	62.3	7.74	65.56
Q07065	Cytoskeleton-associated protein 4	0	36.256	21	8	602	66	5.92	44.5
Q92945	Far upstream element-binding protein 2	0	35.686	27	11	711	73.1	7.3	45.42
P53396-1	ATP-citrate synthase	0	34.5	17	17	1101	120.8	7.33	33.29
Q13501-1	sequestosome-1	0	34.028	45	9	440	47.7	5.22	31.77
Q99832	T-complex protein 1 subunit eta	0	33.871	21	11	543	59.3	7.65	52.69
Q7Z406-1	myosin-14	0	32.722	7	6	1995	227.7	5.6	41.45

P62906	60S ribosomal protein L10A	0	32.605	37	9	217	24.8	9.94	47.04
P02768-1	Serum albumin	0	32.307	12	9	609	69.3	6.28	60.45
P39023	60S ribosomal protein L3	0	31.722	29	11	403	46.1	10.18	38.73
P15880	40S ribosomal protein S2	0	31.579	35	10	293	31.3	10.24	43.15
Q13838-1	spliceosome RNA helicase DDX39B	0	31.522	29	10	428	49	5.67	46.91
Q92841-1	Isoform 2 of Probable ATP-dependent RNA helicase DDX17	0	31.499	17	6	650	72.3	8.59	34.97
Q04637	eukaryotic translation initiation factor 4 gamma 1	0	31.034	9	10	1599	175.4	5.33	34.44
P46777	60S ribosomal protein L5	0	30.628	28	9	297	34.3	9.72	30.89
B9A064-1	Immunoglobulin lambda-like polypeptide 5	0	30.239	31	3	214	23	8.84	37.84
P62277	40S ribosomal protein S13	0	30.037	40	7	151	17.2	10.54	52.01

P62917	60S ribosomal protein L8	0	29.938	43	9	257	28	11.03	52.03
P28799	Granulins	0	29.902	21	11	593	63.5	6.83	44.61
P48643	T-complex protein 1 subunit epsilon	0	29.886	22	11	541	59.6	5.66	40.19
P62241	40S ribosomal protein S8	0	29.653	38	7	208	24.2	10.32	53.21
P17844	probable ATP-dependent RNA helicase DDX5	0	29.653	21	9	614	69.1	8.92	38.18
P13010	X-ray repair cross-complementing protein 5	0	28.955	15	10	732	82.7	5.81	34.63
Q15365	Poly(RC)-binding protein 1	0	28.825	22	3	356	37.5	7.09	40.86
P12004	proliferating cell nuclear antigen	0	28.382	35	7	261	28.8	4.69	40.35
P62826	GTP-binding nuclear protein RAN	0	28.241	39	7	216	24.4	7.49	44.46
P07814	Bifunctional glutamate/proline--tRNA ligase	0	27.963	10	11	1512	170.5	7.33	36.21

P42167	Lamina-associated polypeptide 2, isoforms beta/gamma	0	27.873	26	7	454	50.6	9.38	41.37
P39656	Dolichyl-diphosphooligosaccharide--protein glycosyltransferase 48 kDa subunit	0	27.434	19	7	456	50.8	6.55	43.84
Q14103	heterogeneous nuclear ribonucleoprotein D0	0	26.956	20	6	355	38.4	7.81	28.79
P62280	40S ribosomal protein S11	0	26.791	51	8	158	18.4	10.3	45.27
P61313-1	60S ribosomal protein L15	0	26.763	40	8	204	24.1	11.62	40.18
P62888	60S ribosomal protein L30	0	26.7	59	6	115	12.8	9.63	27.92
P11908	ribose-phosphate pyrophosphokinase 2	0	26.699	20	1	318	34.7	6.61	33.88
P0DOY2	immunoglobulin lambda constant 2	0	26.626	56	2	106	11.3	7.24	39.12
P32969	60S ribosomal protein L9	0	26.14	29	5	192	21.9	9.95	30.62

P15531	Nucleoside diphosphate kinase A	0	26.083	39	1	152	17.1	6.19	42.5
Q12931	heat shock protein 75 kDa, mitochondrial	0	26.074	13	7	704	80.1	8.21	38.66
P61353	60S ribosomal protein L27	0	26.005	42	5	136	15.8	10.56	43.05
P07737	profilin-1	0	25.662	54	7	140	15	8.27	36.11
P08195	4F2 cell-surface antigen heavy chain	0	25.21	15	7	630	68	5.01	35.27
P63173	60s ribosomal protein l38	0	25.036	36	3	70	8.2	10.1	33.88
Q15084-1	Protein disulfide-isomerase A6	0	25.019	24	7	440	48.1	5.08	36.19
P02533	Keratin, type I cytoskeletal 14	0	24.995	18	5	472	51.5	5.16	32.85
P25398	40S ribosomal protein S12	0	24.872	54	6	132	14.5	7.21	35.69
P12236	ADP/ATP translocase 3	0	24.87	34	3	298	32.8	9.74	57.44
Q14697-1	Neutral alpha-glucosidase AB	0	24.654	10	7	944	106.8	6.14	29.48
O14950	Myosin regulatory light chain 12B	0	24.643	44	7	172	19.8	4.84	37.87

P30101	Protein disulfide-isomerase A3	0	24.551	21	8	505	56.7	6.35	25.91
P05388	60S acidic ribosomal protein P0	0	24.492	27	7	317	34.3	5.97	30.39
P46783	40S ribosomal protein S10	0	24.103	38	6	165	18.9	10.15	39.46
P22392	nucleoside diphosphate kinase b	0	23.958	35	1	152	17.3	8.41	38.82
P08708	40S ribosomal protein S17	0	23.848	40	6	135	15.5	9.85	37.06
P63241-1	Eukaryotic translation initiation factor 5A-1	0	23.305	29	6	154	16.8	5.24	25.99
Q9H0U4	ras-related protein Rab-1B	0	23.235	43	2	201	22.2	5.73	31.25
P62820	Ras-related protein Rab-1A	0	23.197	48	4	205	22.7	6.21	35.95
P31948	stress-induced-phosphoprotein 1	0	23.138	17	10	543	62.6	6.8	38.53
P61204	ADP-ribosylation factor 3	0	23.124	39	3	181	20.6	7.43	29.21
P06744	glucose-6-phosphate isomerase	0	23.082	18	11	558	63.1	8.32	33.49
P38919	Eukaryotic initiation factor 4A-III	0	23.016	21	6	411	46.8	6.73	29.18

Q14204	Cytoplasmic dynein 1 heavy chain 1	0	22.99	3	10	4646	532.1	6.4	27.98
P04844-1	Dolichyl-diphosphooligosaccharide--protein glycosyltransferase subunit 2	0	22.936	11	4	631	69.2	5.69	21.27
P05387	60S acidic ribosomal protein P2	0	22.931	53	3	115	11.7	4.54	23.68
P69905	Hemoglobin subunit alpha	0	22.694	18	3	142	15.2	8.68	30.03
P41252	isoleucine--tRNA ligase, cytoplasmic	0	22.558	8	8	1262	144.4	6.15	25.08
Q14974	Importin subunit beta-1	0	22.452	9	6	876	97.1	4.78	33.28
P26373-1	60S ribosomal protein L13	0	22.448	32	7	211	24.2	11.65	38.6
P27635	60S ribosomal protein L10	0	22.271	25	7	214	24.6	10.08	35.04
P30041	Peroxiredoxin-6	0	22.118	40	7	224	25	6.38	31.2
P21333	Filamin-A	0	22.094	5	9	2647	280.6	6.06	25.1
Q9NVI7-2	Isoform 2 of ATPase family AAA domain-containing protein 3A	0	21.975	13	7	586	66.2	9.25	32.55

P62244	40S ribosomal protein S15a	0	21.966	39	6	130	14.8	10.13	25.72
P62258-1	14-3-3 protein epsilon	0	21.799	35	7	255	29.2	4.74	34.49
Q9Y230	RuvB-like 2	0	21.471	17	6	463	51.1	5.64	25.84
O00571	ATP-dependent RNA helicase DDX3X	0	21.459	12	7	662	73.2	7.18	35.86
Q99729-3	Isoform 3 of Heterogeneous nuclear ribonucleoprotein A/B	0	21.332	27	5	285	30.6	7.91	25.83
P52597	Heterogeneous nuclear ribonucleoprotein F	0	21.156	18	4	415	45.6	5.58	25.4
P39019	40S ribosomal protein S19	0	21.137	49	9	145	16.1	10.32	43.5
P63104-1	14-3-3 protein zeta/delta	0	20.788	29	4	245	27.7	4.79	34.47
Q02543	60S ribosomal protein L18a	0	20.749	39	7	176	20.7	10.71	27.78
P10412	Histone H1.4	0	20.74	28	1	219	21.9	11.03	32.42
P25205	DNA replication licensing factor mcm3	0	20.622	12	8	808	90.9	5.77	26.54

P51149	ras-related protein Rab-7a	0	20.582	39	6	207	23.5	6.7	25.67
P02786	Transferrin receptor protein 1	0	20.443	13	8	760	84.8	6.61	25.22
P62269	40S ribosomal protein S18	0	20.373	49	9	152	17.7	10.99	45.57
P62979	Ubiquitin-40S ribosomal protein S27a	0	20.349	34	4	156	18	9.64	24.88
P08865	40S ribosomal protein SA	0	20.335	31	5	295	32.8	4.87	22.77
P60866	40S ribosomal protein S20	0	20.271	23	3	119	13.4	9.94	34.72
P61586	Transforming protein RhoA	0	20.018	39	7	193	21.8	6.1	20.15
P29966	Myristoylated alanine-rich C-kinase substrate	0	19.983	27	5	332	31.5	4.45	37.3
Q96AE4	Far upstream element-binding protein 1	0	19.866	18	7	644	67.5	7.61	22.91
P51659-1	peroxisomal multifunctional enzyme type 2	0	19.795	10	5	736	79.6	8.84	18.19
P16403	Histone H1.2	0	19.777	30	1	213	21.4	10.93	32.37

Q12905	Interleukin enhancer-binding factor 2	0	19.654	14	4	390	43	5.26	13.67
Q14566	DNA replication licensing factor MCM6	0	19.627	11	7	821	92.8	5.41	19.56
Q16891	MICOS complex subunit Mic60	0	19.579	10	6	758	83.6	6.48	21.18
O60506	Heterogeneous nuclear ribonucleoprotein Q	0	19.53	12	2	623	69.6	8.59	21.82
P35613	Basigin	0	19.474	17	6	385	42.2	5.66	24.19
Q12797	Aspartyl/Asparaginyl beta-hydroxylase	0	19.373	7	3	758	85.8	5.01	16.14
P51991-1	Heterogeneous nuclear ribonucleoprotein A3	0	19.348	14	2	378	39.6	9.01	21.74
P23246-1	splicing factor, proline- and glutamine-rich	0	19.208	10	5	707	76.1	9.44	25.86
Q96JJ3	Engulfment and cell motility protein 2	0	19.194	6	1	720	82.6	5.9	40.09
P62829	60S ribosomal protein L23	0	19.183	55	7	140	14.9	10.51	33.68

Q9Y490	Talin-1	0	19.005	3	5	2541	269.6	6.07	22.32
Q8NC51-1	Plasminogen activator inhibitor 1 RNA-binding protein	0	18.979	13	4	408	44.9	8.65	26.81
P62753	40S RIBOSOMAL PROTEIN S6	0	18.972	29	7	249	28.7	10.84	39.18
P46781	40S ribosomal protein S9	0	18.947	46	10	194	22.6	10.65	43.61
P05787	Keratin, type II cytoskeletal 8	0	18.906	10	1	483	53.7	5.59	36.66
Q86VP6-1	cullin-associated nedd8-dissociated protein 1	0	18.877	7	7	1230	136.3	5.78	19.96
Q12906-1	Interleukin enhancer-binding factor 3	0	18.681	10	7	894	95.3	8.76	21.59
P09972	Fructose-bisphosphate aldolase C	0	18.3	13	2	364	39.4	6.87	31.55
P62249	40S ribosomal protein S16	0	18.037	55	9	146	16.4	10.21	33.69
P24752	Acetyl-CoA acetyltransferase, mitochondrial	0	17.997	12	4	427	45.2	8.85	23.55

P18124	60S ribosomal protein L7	0	17.83	34	9	248	29.2	10.65	34.24
P53985	Monocarboxylate transporter 1	0	17.694	9	4	500	53.9	8.66	20.11
Q9H9B4	Sideroflexin-1	0	17.559	21	6	322	35.6	9.07	27.12
P83731	60S ribosomal protein L24	0	17.504	43	9	157	17.8	11.25	29.54
P16615	Sarcoplasmic/endoplasmic reticulum calcium ATPase 2	0	17.355	7	6	1042	114.7	5.34	18.14
P07910-1	Heterogeneous nuclear ribonucleoproteins C1/C2	0	17.314	24	7	306	33.7	5.08	29.42
P18085	ADP-ribosylation factor 4	0	17.23	32	3	180	20.5	7.14	24.96
P34932	Heat shock 70 kDa protein 4	0	17.189	10	6	840	94.3	5.19	15.54
P46778	60S ribosomal protein L21	0	17.037	20	3	160	18.6	10.49	28.83
P36542-1	ATP synthase subunit gamma, mitochondrial	0	16.822	18	5	298	33	9.22	24.5

Q9Y383	Putative RNA-binding protein Luc7-like 2	0	16.752	15	5	392	46.5	10.01	27.91
Q13151	Heterogeneous nuclear ribonucleoprotein A0	0	16.697	25	5	305	30.8	9.29	15.92
Q9Y265	RuvB-like 1	0	16.554	14	6	456	50.2	6.42	18.67
P50914	60S ribosomal protein L14	0	16.382	23	5	215	23.4	10.93	36.18
P49588	Alanine--tRNA ligase, cytoplasmic	0	16.314	7	6	968	106.7	5.53	19.99
O43707	Alpha-actinin-4	0	16.253	6	5	911	104.8	5.44	14.5
Q15233	Non-POU domain-containing octamer-binding protein	0	16.216	10	5	471	54.2	8.95	19.37
P24539	ATP synthase F(0) complex subunit B1, mitochondrial	0	16.154	20	5	256	28.9	9.36	24.19
O00231	26S proteasome non-ATPase regulatory	0	16.104	14	5	422	47.4	6.48	15.24

	subunit 11								
B5ME19	eukaryotic translation initiation factor 3 subunit C-like protein	0	16.014	8	6	914	105.4	5.64	16.37
P09429	High mobility group protein B1	0	15.895	27	4	215	24.9	5.74	19.21
P05455	Lupus La protein	0	15.655	15	5	408	46.8	7.12	18.64
P67809	Nuclease-sensitive element-binding protein 1	0	15.635	17	2	324	35.9	9.88	13.39
P09211	Glutathione S-transferase P	0	15.543	25	4	210	23.3	5.64	25.05
P27348	14-3-3 protein theta	0	15.534	24	3	245	27.7	4.78	30.41
P23526-1	Adenosylhomocysteinase	0	15.326	12	4	432	47.7	6.34	14.92
P46779	60S ribosomal protein L28	0	15.216	42	7	137	15.7	12.02	28.74
P62263	40S ribosomal protein S14	0	15.216	38	4	151	16.3	10.05	25.19
P17812	CTP synthase 1	0	15.179	10	4	591	66.6	6.46	17.7

O43390-1	heterogeneous nuclear ribonucleoprotein r	0	14.97	11	2	633	70.9	8.13	12.61
P84098	60S ribosomal protein L19	0	14.825	27	5	196	23.5	11.47	24.2
P30153	serine/threonine-protein phosphatase 2A 65 kDa regulatory subunit A alpha isoform	0	14.62	8	4	589	65.3	5.11	17.67
P37802	Transgelin-2	0	14.538	30	6	199	22.4	8.25	23.96
P61513	60S ribosomal protein L37a	0	14.432	42	3	92	10.3	10.43	27.52
P42677	40S ribosomal protein S27	0	14.431	38	3	84	9.5	9.45	22.45
P52907	F-actin-capping protein subunit alpha-1	0	14.4	17	3	286	32.9	5.69	17.06
O14980	Exportin-1	0	14.323	5	5	1071	123.3	6.06	14.21
P62910	60S ribosomal protein L32	0	14.303	22	3	135	15.9	11.33	14.88
O15372	Eukaryotic translation initiation factor 3	0	14.272	15	4	352	39.9	6.54	14.86

	subunit H								
P24534	Elongation factor 1-beta	0	14.188	24	4	225	24.7	4.67	20.79
P62333	26S proteasome regulatory subunit 10B	0	14.137	14	5	389	44.1	7.49	14.81
P29692	Elongation factor 1-delta	0	14.122	18	4	281	31.1	5.01	17.96
O00264	Membrane-associated progesterone receptor component 1	0	14.059	26	3	195	21.7	4.7	21.22
P54136-1	arginine--tRNA ligase, cytoplasmic	0	13.996	9	5	660	75.3	6.68	15.93
P20742	Pregnancy zone protein	0	13.786	2	1	1482	163.8	6.38	18.9
Q00325-1	Phosphate carrier protein, mitochondrial	0	13.664	15	7	362	40.1	9.38	24.49
O95433	activator of 90 kDa heat shock protein ATPase homolog 1	0	13.648	17	4	338	38.3	5.53	12.3
P62913	60S ribosomal protein L11	0	13.615	29	5	178	20.2	9.6	19.29
P13667	Protein disulfide-isomerase A4	0	13.368	9	4	645	72.9	5.07	15.96

P48047	ATP synthase subunit O, mitochondrial	0	13.313	25	4	213	23.3	9.96	17.8
P00918	Carbonic anhydrase 2	0	13.271	18	4	260	29.2	7.4	20.29
P46782	40S ribosomal protein S5	0	13.226	24	6	204	22.9	9.72	21.52
Q9P2E9-1	Ribosome-binding protein 1	0	13.211	4	4	1410	152.4	8.6	11.91
Q9UJZ1	Stomatin-like protein 2, mitochondrial	0	13.211	12	3	356	38.5	7.39	14.75
P19474	E3 ubiquitin-protein ligase TRIM21	0	13.125	12	5	475	54.1	6.38	18.61
P31946	14-3-3 protein beta/alpha	0	13.102	15	1	246	28.1	4.83	24.68
P33993-1	DNA replication licensing factor MCM7	0	13.053	8	5	719	81.3	6.46	11.37
Q16643	drebrin	0	12.943	6	2	649	71.4	4.45	13
Q08211	Atp-dependent rna helicase a	0	12.92	2	2	1270	140.9	6.84	9.18
Q15181	Inorganic pyrophosphatase	0	12.912	19	5	289	32.6	5.86	7.42
P26599	Polypyrimidine tract-binding protein 1	0	12.898	10	5	531	57.2	9.17	18.7
Q92499	ATP-dependent RNA helicase DDX1	0	12.792	8	5	740	82.4	7.23	9.22

Q9P2J5	Leucine--tRNA ligase, cytoplasmic	0	12.731	4	4	1176	134.4	7.3	12.32
P62851	40S ribosomal protein S25	0	12.661	41	5	125	13.7	10.11	21.33
P62805	histone H4	0	12.641	50	5	103	11.4	11.36	23.02
P42704	Leucine-rich PPR motif-containing protein, mitochondrial	0	12.598	4	6	1394	157.8	6.13	10.17
O15371	Eukaryotic translation initiation factor 3 subunit D	0	12.566	9	4	548	63.9	6.05	11.24
Q07020	60S ribosomal protein L18	0	12.511	27	5	188	21.6	11.72	24.07
P11177	Pyruvate dehydrogenase E1 component subunit beta, mitochondrial	0	12.444	18	5	359	39.2	6.65	13.19
P00387	NADH-cytochrome b5 reductase 3	0	12.21	15	4	301	34.2	7.59	19.39
P62847-1	40S ribosomal protein S24	0	12.121	17	2	133	15.4	10.78	14.81
P62750	60S ribosomal protein L23a	0	12.006	30	4	156	17.7	10.45	17.7

P46776	60S ribosomal protein L27a	0	11.991	28	4	148	16.6	11	17.9
P61254	60S ribosomal protein L26	0	11.9	34	6	145	17.2	10.55	27.11
O75964	ATP synthase subunit g, mitochondrial	0	11.765	34	3	103	11.4	9.64	13.45
O76094	Signal recognition particle subunit SRP72	0	11.692	8	4	671	74.6	9.26	10.65
P56192	Methionine--tRNA ligase, cytoplasmic	0	11.685	5	4	900	101.1	6.16	14.29
Q13310	Polyadenylate-binding protein 4	0	11.651	7	2	644	70.7	9.26	14.92
P01023	alpha-2-macroglobulin	0	11.628	3	3	1474	163.2	6.46	19.6
Q9P035	Very-long-chain (3R)-3-hydroxyacyl-CoA dehydratase 3	0	11.435	10	2	362	43.1	8.94	13
P62491-1	Ras-related protein Rab-11A	0	11.305	21	5	216	24.4	6.57	15.52
O95831-1	Apoptosis-inducing factor 1, mitochondrial	0	11.258	7	3	613	66.9	8.95	11.3

Q9NZI8	Insulin-like growth factor 2 mRNA-binding protein 1	0	11.198	11	6	577	63.4	9.2	13.53
P04181-1	Ornithine aminotransferase, mitochondrial	0	11.037	12	4	439	48.5	7.03	7.72
P01857	Immunoglobulin heavy constant gamma 1	0	10.904	12	3	330	36.1	8.19	21.1
P14866	Heterogeneous nuclear ribonucleoprotein L	0	10.778	12	5	589	64.1	8.22	10.08
P60174-1	Isoform 2 of Triosephosphate isomerase	0	10.776	25	4	249	26.7	6.9	18.93
Q00341-1	Vigilin	0	10.637	3	4	1268	141.4	6.87	7.89
P39748	Flap endonuclease 1	0	10.609	11	3	380	42.6	8.62	14.09
P51148	Ras-related protein Rab-5C	0	10.555	17	3	216	23.5	8.41	9.37
P16989-1	Y-box-binding protein 3	0	10.515	10	1	372	40.1	9.77	9.52

O75821	Eukaryotic translation initiation factor 3 subunit G	0	10.468	12	4	320	35.6	6.13	11.19
P26640	Valine--tRNA ligase	0	10.461	5	5	1264	140.4	7.59	6.75
O00232-1	26s proteasome non-atpase regulatory subunit 12	0	10.378	10	3	456	52.9	7.65	11.64
Q13283	Ras GTPase-activating protein-binding protein 1	0	10.253	10	3	466	52.1	5.52	5.7
Q7KZF4	staphylococcal nuclease domain-containing protein 1	0	10.234	7	4	910	101.9	7.17	10.48
O95373	Importin-7	0	10.122	4	4	1038	119.4	4.82	11.45
P40939	Trifunctional enzyme subunit alpha, mitochondrial	0	10.001	5	3	763	82.9	9.04	11.02
P62266	40S ribosomal protein S23	0	9.981	29	4	143	15.8	10.49	20.29

P20700	Lamin-B1	0	9.977	6	4	586	66.4	5.16	13.56
Q9Y262	eukaryotic translation initiation factor 3 subunit L	0	9.96	9	4	564	66.7	6.34	7.82
Q8N1I0	Dedicator of cytokinesis protein 4	0	9.942	2	5	1966	225.1	7.65	12.5
P55209	Nucleosome assembly protein 1-like 1	0	9.894	7	2	391	45.3	4.46	13.56
Q96AG4	Leucine-rich repeat-containing protein 59	0	9.878	14	3	307	34.9	9.57	13.47
P13073	Cytochrome c oxidase subunit 4 isoform 1, mitochondrial	0	9.856	24	4	169	19.6	9.51	20.74
Q53GQ0	Very-long-chain 3-oxoacyl-CoA reductase	0	9.845	14	3	312	34.3	9.32	11.2
P50395-1	Rab GDP dissociation inhibitor beta	0	9.806	13	4	445	50.6	6.47	13.83
Q9Y277	Voltage-dependent anion-selective	0	9.606	14	2	283	30.6	8.66	13.16

	channel protein 3								
Q14498-1	RNA-binding protein 39	0	9.564	5	2	530	59.3	10.1	11.25
Q16576-1	Histone-binding protein RBBP7	0	9.513	8	3	425	47.8	5.05	10.75
P26196	Probable ATP-dependent RNA helicase DDX6	0	9.503	9	2	483	54.4	8.66	12.93
P62195-1	26S proteasome regulatory subunit 8	0	9.473	11	3	406	45.6	7.55	10.86
P61006	Ras-related protein Rab-8A	0	9.469	19	2	207	23.7	9.07	8.65
P61026	ras-related protein rab-10	0	9.451	15	2	200	22.5	8.38	10.32
Q9Y3I0	tRNA-splicing ligase RtcB homolog	0	9.433	11	4	505	55.2	7.23	7.29
P43686	26S proteasome regulatory subunit 6B	0	9.35	10	2	418	47.3	5.21	4.23
Q9Y2L1-1	exosome complex exonuclease RRP44	0	9.298	4	3	958	108.9	7.14	8.52
Q9NX00	Transmembrane protein 160	0	9.277	22	5	188	19.6	8.03	17.63
P62081	40S ribosomal protein S7	0	9.269	19	4	194	22.1	10.1	17.97

P50402	Emerin	0	9.251	19	4	254	29	5.5	10.61
Q13200	26S proteasome non-ATPase regulatory subunit 2	0	9.238	5	3	908	100.1	5.2	10.56
P49591	Serine--tRNA ligase, cytoplasmic	0	9.193	9	3	514	58.7	6.43	8.57
Q16777	Histone H2A type 2-C	0	9.133	27	3	129	14	10.9	17.1
P18669	Phosphoglycerate mutase 1	0	9.039	11	3	254	28.8	7.18	10.19
O94832	Unconventional myosin-Id	0	9.029	5	5	1006	116.1	9.39	9.87
P51572	B-cell receptor-associated protein 31	0	9.027	16	5	246	28	8.44	15.49
P47897	glutamine--tRNA ligase	0	8.941	4	2	775	87.7	7.15	9.04
Q96FW1	Ubiquitin thioesterase otub1	0	8.906	16	4	271	31.3	4.94	9.03
Q9Y5M8	signal recognition particle receptor subunit beta	0	8.802	12	2	271	29.7	9.04	6.7
P07237	Protein disulfide-isomerase	0	8.782	6	2	508	57.1	4.87	10.02

Q8IZP2	Putative protein FAM10A4	0	8.669	19	4	240	27.4	5.08	5.22
Q8WUM4	Programmed cell death 6-interacting protein	0	8.517	4	3	868	96	6.52	7.33
Q14739	Lamin-B receptor	0	8.507	6	4	615	70.7	9.36	16.89
P61981	14-3-3 protein gamma	0	8.47	15	1	247	28.3	4.89	18.94
O95292	Vesicle-associated membrane protein-associated protein B/C	0	8.454	17	3	243	27.2	7.3	11.09
Q9BZZ5-4	Apoptosis inhibitor 5	0	8.408	5	2	524	59	7.34	6.75
P62873	Guanine nucleotide-binding protein G(I)/G(S)/G(T) subunit beta-1	0	8.35	10	3	340	37.4	6	11.79
O15173	Membrane-associated progesterone receptor component 2	0	8.337	13	1	223	23.8	4.88	14.24
Q01081	Splicing factor U2AF 35 kDa subunit	0	8.314	19	3	240	27.9	8.81	7.9

Q9Y6C9	Mitochondrial carrier homolog 2	0	8.154	11	2	303	33.3	7.97	14.06
P41250	Glycine--tRNA ligase	0	8.104	5	4	739	83.1	7.03	8.75
Q9P0L0	vesicle-associated membrane protein-associated protein A	0	8.083	13	3	249	27.9	8.62	11.87
Q9BXP5	serrate RNA effector molecule homolog	0	8.06	4	3	876	100.6	5.96	7.73
P51571	translocon-associated protein subunit delta	0	8.019	20	3	173	19	6.15	9.17
P47914	60S ribosomal protein L29	0	7.992	14	2	159	17.7	11.66	6.84
Q96A26	Protein FAM162A	0	7.973	17	3	154	17.3	9.77	19.33
P06493	Cyclin-dependent kinase 1	0	7.941	10	2	297	34.1	8.4	13.1
Q14152	Eukaryotic translation initiation factor 3 subunit A	0	7.918	3	4	1382	166.5	6.79	12.49
P32119	Peroxiredoxin-2	0	7.851	18	3	198	21.9	5.97	4.87

P02765	Alpha-2-HS-glycoprotein	0	7.837	5	3	367	39.3	5.72	10.63
Q92973-1	transportin-1	0	7.828	5	3	898	102.3	4.98	7.56
P62854	40S ribosomal protein S26	0	7.775	34	3	115	13	11	6.98
P49755	Transmembrane emp24 domain-containing protein 10	0	7.721	10	2	219	25	7.44	7.3
Q9UMR2-1	ATP-dependent RNA helicase DDX19B	0	7.702	7	3	479	53.9	6.3	9.18
O75396	Vesicle-trafficking protein SEC22b	0	7.669	11	2	215	24.6	6.92	12.59
Q07666	KH domain-containing, RNA-binding, signal transduction-associated protein 1	0	7.667	7	3	443	48.2	8.66	8.34
Q93050-1	Isoform 2 of V-type proton ATPase 116 kDa subunit a isoform 1	0	7.579	5	3	831	95.7	6.65	8.56
P61019-1	Ras-related protein Rab-2A	0	7.546	13	2	212	23.5	6.54	6.74

P40429	60S ribosomal protein L13a	0	7.537	19	5	203	23.6	10.93	11.45
Q13148-1	TAR DNA-binding protein 43	0	7.525	8	3	414	44.7	6.19	9.02
P01859	Immunoglobulin heavy constant gamma 2	0	7.482	10	1	326	35.9	7.59	11.93
Q00688	peptidyl-prolyl cis-trans isomerase FKBP3	0	7.462	15	2	224	25.2	9.28	3.58
P60660	Myosin light polypeptide 6	0	7.391	15	2	151	16.9	4.65	13.27
P34897-1	Serine hydroxymethyltransferase, mitochondrial	0	7.388	7	3	504	56	8.53	10.2
P52789	Hexokinase-2	0	7.319	4	4	917	102.3	6.05	9.4
O95573	long-chain-fatty-acid--CoA ligase 3	0	7.296	3	2	720	80.4	8.38	9.28
Q96H79	Zinc finger CCCH-type antiviral protein 1-like	0	7.282	10	2	300	32.9	8.13	3.17

P49207	60S ribosomal protein L34	0	7.249	28	4	117	13.3	11.47	17.83
O43809	Cleavage and polyadenylation specificity factor subunit 5	0	7.145	8	1	227	26.2	8.82	9.59
P49321	Nuclear autoantigenic sperm protein	0	7.136	5	2	788	85.2	4.3	8.04
Q9Y3F4	Serine-threonine kinase receptor-associated protein	0	7.058	7	2	350	38.4	5.12	7.03
P01619	Immunoglobulin kappa variable 3-20	0	7.038	14	1	116	12.5	4.96	9.06
Q9UMS4	Pre-mRNA-processing factor 19	0	7.003	8	2	504	55.1	6.61	5.64
P49257	Protein ERGIC-53	0	7.001	7	2	510	57.5	6.77	7.11
Q9NZ45	CDGSH iron-sulfur domain-containing protein 1	0	6.963	14	1	108	12.2	9.09	11.82
Q96HS1-1	Serine/threonine-protein phosphatase Pgam5, mitochondrial	0	6.918	11	3	289	32	8.68	13.41

P42766	60S ribosomal protein L35	0	6.814	15	2	123	14.5	11.05	13.17
P61604	10 kDa heat shock protein, mitochondrial	0	6.804	35	3	102	10.9	8.92	6.17
Q9BWF3-1	RNA-binding protein 4	0	6.661	7	2	364	40.3	7.08	13.4
Q12904-1	aminoacyl tRNA synthase complex-interacting multifunctional protein 1	0	6.658	15	3	312	34.3	8.43	5.55
P53618	Coatomer subunit beta	0	6.652	3	2	953	107.1	6.05	10.22
P62714	serine/threonine-protein phosphatase 2A catalytic subunit beta isoform	0	6.624	10	2	309	35.6	5.43	5.68
Q8WWM7-1	ataxin-2-like protein	0	6.624	4	3	1075	113.3	8.59	9.21
Q16543	Hsp90 co-chaperone Cdc37	0	6.601	7	2	378	44.4	5.25	5.76
P33992	DNA replication licensing factor mcm5	0	6.548	6	3	734	82.2	8.37	5.08

P40925	Malate dehydrogenase, cytoplasmic	0	6.464	7	2	334	36.4	7.36	8.29
Q9BVK6	Transmembrane emp24 domain-containing protein 9	0	6.458	11	3	235	27.3	8.02	7.52
Q9UNM6	26S proteasome non-ATPase regulatory subunit 13	0	6.392	7	3	376	42.9	5.81	8.7
P53621-1	coatomer subunit alpha	0	6.375	3	4	1224	138.3	7.66	8.5
O75643-1	U5 small nuclear ribonucleoprotein 200 kDa helicase	0	6.337	2	2	2136	244.4	6.06	4.91
P13674-1	prolyl 4-hydroxylase subunit alpha-1	0	6.312	4	2	534	61	6.01	5.23
P11802	Cyclin-dependent kinase 4	0	6.277	7	1	303	33.7	7.01	5.74
P33991	DNA replication licensing factor MCM4	0	6.274	3	2	863	96.5	6.74	7.06
P62899	60S ribosomal protein L31	0	6.262	18	2	125	14.5	10.54	8.98
P62891	60S ribosomal protein L39	0	6.224	20	1	51	6.4	12.56	10.27

Q9NRN7	L-aminoadipate-semialdehyde dehydrogenase-phosphopantetheinyl transferase	0	6.208	5	1	309	35.8	6.8	10.9
P12532	Creatine kinase U-type, mitochondrial	0	6.186	5	2	417	47	8.34	5.92
O43242	26S proteasome non-ATPase regulatory subunit 3	0	6.168	4	2	534	60.9	8.44	8.03
Q9UNF1	Melanoma-associated antigen D2	0	6.109	4	1	606	64.9	9.32	0
Q9Y3U8	60S ribosomal protein L36	0	6.084	24	3	105	12.2	11.59	7.29
Q9NX63	MICOS complex subunit MIC19	0	6.073	11	2	227	26.1	8.28	8.68
Q92598	Heat shock protein 105 kDa	0	6.063	3	2	858	96.8	5.39	10.01
Q9H3N1	Thioredoxin-related transmembrane protein 1	0	6.02	8	2	280	31.8	4.98	4.6
P55084	Trifunctional enzyme subunit beta,	0	6.015	9	3	474	51.3	9.41	6.35

	mitochondrial								
P08559	Pyruvate dehydrogenase E1 component subunit alpha, somatic form, mitochondrial	0	5.995	6	2	390	43.3	8.06	7.04
Q2TAY7	WD40 repeat-containing protein SMU1	0	5.761	7	2	513	57.5	7.18	7.44
P62841	40S ribosomal protein S15	0	5.756	8	1	145	17	10.39	8.2
P35606	Coatomer subunit beta'	0	5.723	2	2	906	102.4	5.27	6.85
P33176	Kinesin-1 heavy chain	0	5.688	2	1	963	109.6	6.51	3.4
P55072	Transitional endoplasmic reticulum ATPase	0	5.655	3	2	806	89.3	5.26	8.89
P52292	Importin subunit alpha-1	0	5.579	3	1	529	57.8	5.4	4.06
P60981-1	Destrin	0	5.539	18	3	165	18.5	7.85	3.09
P22695	Cytochrome b-c1 complex subunit 2,	0	5.532	5	2	453	48.4	8.63	3.66

	mitochondrial								
P62140	Serine/threonine-protein phosphatase PP1-beta catalytic subunit	0	5.52	11	3	327	37.2	6.19	4.38
Q5QNW6-1	Histone H2B type 2-F	0	5.52	20	3	126	13.9	10.32	9.2
O43169	Cytochrome b5 type B	0	5.501	23	1	146	16.3	4.97	4.73
O43776	Asparagine--tRNA ligase, cytoplasmic	0	5.476	6	2	548	62.9	6.25	3.97
P07099	epoxide hydrolase 1	0	5.456	5	2	455	52.9	7.25	6.64
Q9P258	Protein RCC2	0	5.448	7	3	522	56	8.78	7.04
P62306	Small nuclear ribonucleoprotein F	0	5.421	24	2	86	9.7	4.67	9.73
Q96CS3	FAS-associated factor 2	0	5.369	6	3	445	52.6	5.62	11.52
P05534	HLA class I histocompatibility antigen, A-24 alpha chain	0	5.354	7	1	365	40.7	6.34	9.56

P18077	60S ribosomal protein L35a	0	5.282	25	3	110	12.5	11.06	11.62
O75400	pre-mRNA-processing factor 40 homolog A	0	5.247	3	2	957	108.7	7.56	5.91
P27144	Adenylate kinase 4, mitochondrial	0	5.235	10	2	223	25.3	8.4	8.46
Q96BM9	ADP-ribosylation factor-like protein 8A	0	5.231	17	2	186	21.4	7.77	5.52
P61163	Alpha-centractin	0	5.172	9	2	376	42.6	6.64	5.35
Q13347	Eukaryotic translation initiation factor 3 subunit I	0	5.151	9	3	325	36.5	5.64	4.23
Q14166	Tubulin--tyrosine ligase-like protein 12	0	5.109	4	2	644	74.4	5.53	5.07
Q14008	Cytoskeleton-associated protein 5	0	5.1	2	2	2032	225.4	7.8	6.23
Q13162	Peroxiredoxin-4	0	5.068	11	2	271	30.5	6.29	5.84
Q15428	splicing factor 3a subunit 2	0	5.033	3	1	464	49.2	9.64	6.7
Q15836	Vesicle-associated membrane protein 3	0	5.031	17	1	100	11.3	8.79	7.7

Q13547	histone deacetylase 1	0	4.976	5	2	482	55.1	5.48	6.95
O60749	Sorting nexin-2	0	4.974	5	2	519	58.4	5.12	4.94
Q99497	protein/nucleic acid deglycase DJ-1	0	4.969	16	3	189	19.9	6.79	9.29
O75489	NADH dehydrogenase [ubiquinone] iron-sulfur protein 3, mitochondrial	0	4.908	10	2	264	30.2	7.5	9.7
P19623	spermidine synthase	0	4.857	11	2	302	33.8	5.49	3.9
Q96C36	Pyrroline-5-carboxylate reductase 2	0	4.848	7	2	320	33.6	7.77	3.68
Q9NTJ3-1	Structural maintenance of chromosomes protein 4	0	4.822	2	2	1288	147.1	6.79	5.32
Q15029	116 kDa U5 small nuclear ribonucleoprotein component	0	4.812	2	1	972	109.4	5	7.49
O75534	cold shock domain-containing protein E1	0	4.809	3	3	798	88.8	6.25	6.35

P48444	Coatomer subunit delta	0	4.788	4	2	511	57.2	6.21	2.42
Q9Y2Q3-1	Glutathione S-transferase kappa 1	0	4.781	11	2	226	25.5	8.41	6.96
P62495	Eukaryotic peptide chain release factor subunit 1	0	4.777	5	2	437	49	5.71	4.54
P78347	General transcription factor II-I	0	4.767	2	2	998	112.3	6.39	4.09
P55786	puromycin-sensitive aminopeptidase	0	4.724	2	2	919	103.2	5.72	7.68
P60228	Eukaryotic translation initiation factor 3 subunit E	0	4.704	4	2	445	52.2	6.04	7.15
O00139	Kinesin-like protein KIF2A	0	4.683	2	1	706	79.9	6.68	2.86
P49915	GMP synthase [glutamine-hydrolyzing]	0	4.661	3	2	693	76.7	6.87	8.36
P02042	Hemoglobin subunit delta	0	4.638	13	2	147	16	8.05	12.01
Q16186	Proteasomal ubiquitin receptor ADRM1	0	4.637	4	1	407	42.1	5.07	6.72
Q969Q0	60S ribosomal protein L36a-like	0	4.633	16	2	106	12.5	10.65	7.25

Q9Y266	nuclear migration protein nudC	0	4.633	9	3	331	38.2	5.38	6.02
Q9UHB9	Signal recognition particle subunit SRP68	0	4.62	3	2	627	70.7	8.56	4.68
Q9NYF8-1	Bcl-2-associated transcription factor 1	0	4.615	1	1	920	106.1	9.98	4.96
P35268	60S ribosomal protein L22	0	4.604	10	1	128	14.8	9.19	8.48
P29401	Transketolase	0	4.599	6	2	623	67.8	7.66	2.16
Q9Y4L1	Hypoxia up-regulated protein 1	0	4.593	2	1	999	111.3	5.22	2.78
P13489	Ribonuclease inhibitor	0	4.592	5	2	461	49.9	4.82	4.67
O14929	histone acetyltransferase type B catalytic subunit	0	4.579	3	1	419	49.5	5.69	3.24
Q92616	eIF-2-alpha kinase activator GCN1	0	4.5	0	1	2671	292.6	7.47	3.19
P27105	erythrocyte band 7 integral membrane protein	0	4.478	10	2	288	31.7	7.88	4.94

P62857	40S ribosomal protein S28	0	4.476	30	2	69	7.8	10.7	4.59
P27708	CAD protein	0	4.467	2	3	2225	242.8	6.46	3.86
P22061	protein-L-isoaspartate(D-aspartate) O-methyltransferase	0	4.466	7	1	227	24.6	7.21	3.52
Q7RTS7	Keratin, type II cytoskeletal 74	0	4.459	5	1	529	57.8	7.71	18.27
P80748	Immunoglobulin lambda variable 3-21	0	4.441	14	1	117	12.4	5.29	4.38
Q99460	26S proteasome non-ATPase regulatory subunit 1	0	4.414	2	2	953	105.8	5.39	2.62
Q9UHX1-1	poly(U)-binding-splicing factor PUF60	0	4.409	7	2	559	59.8	5.29	4.46
P61803	Dolichyl-diphosphooligosaccharide--protein glycosyltransferase subunit DAD1	0	4.397	19	2	113	12.5	7.08	7.13
P63167	Dynein light chain 1, cytoplasmic	0	4.393	20	2	89	10.4	7.4	4.06

P23284	peptidyl-prolyl cis-trans isomerase B	0	4.385	9	1	216	23.7	9.41	5.75
P47756-1	F-actin-capping protein subunit beta	0	4.379	5	1	277	31.3	5.59	2.9
P55036-1	26S proteasome non-ATPase regulatory subunit 4	0	4.374	7	1	377	40.7	4.79	8.03
Q99541	perilipin-2	0	4.371	5	2	437	48	6.8	7.08
P51570	galactokinase	0	4.367	5	2	392	42.2	6.46	9.45
P62861	40S ribosomal protein S30	0	4.331	31	2	59	6.6	12.15	6.41
Q8N8S7	Protein enabled homolog	0	4.316	5	2	591	66.5	6.93	3.5
P38606	V-type proton ATPase catalytic subunit A	0	4.312	4	2	617	68.3	5.52	4.28
Q9NRP0-1	oligosaccharyltransferase complex subunit OSTC	0	4.29	8	1	149	16.8	9.13	6.29
O94905-1	Erlin-2	0	4.269	6	2	339	37.8	5.62	3.07

Q8NE71-1	ATP-binding cassette sub-family F member 1	0	4.268	3	2	845	95.9	6.8	2.52
O00429	Dynamin-1-like protein	0	4.266	2	1	736	81.8	6.81	2.65
P62316	Small nuclear ribonucleoprotein Sm D2	0	4.235	16	2	118	13.5	9.91	5.17
P43487	Ran-specific GTPase-activating protein	0	4.225	9	2	201	23.3	5.29	4.6
P54709	sodium/potassium-transporting ATPase subunit beta-3	0	4.214	5	1	279	31.5	8.35	6.21
P20042	Eukaryotic translation initiation factor 2 subunit 2	0	4.205	6	1	333	38.4	5.8	3.8
Q13247	Serine/arginine-rich splicing factor 6	0	4.204	5	1	344	39.6	11.43	4.15
Q9H773	dCTP pyrophosphatase 1	0	4.176	8	1	170	18.7	5.03	6.75
O75369-1	Filamin-B	0	4.176	1	2	2602	278	5.73	2.59
P15170	Eukaryotic peptide chain release factor	0	4.167	4	2	499	55.7	5.62	5.22

	GTP-binding subunit ERF3A								
P61224-1	Ras-related protein Rap-1b	0	4.165	11	2	184	20.8	5.78	5.01
P49756-1	RNA-binding protein 25	0	4.165	2	1	843	100.1	6.32	3.39
Q15459	splicing factor 3A subunit 1	0	4.102	2	1	793	88.8	5.22	5.76
Q9NS69	Mitochondrial import receptor subunit TOM22 homolog	0	4.083	18	1	142	15.5	4.34	4.42
Q01650	large neutral amino acids transporter small subunit 1	0	4.073	4	1	507	55	7.72	2.98
Q12792-2	Twinfilin-1	0	4.073	8	2	350	40.3	6.96	2.37
O00487	26S proteasome non-ATPase regulatory subunit 14	0	4.062	6	2	310	34.6	6.52	7.56
P35998	26S proteasome regulatory subunit 7	0	4.055	4	2	433	48.6	5.95	4.76
P61088	ubiquitin-conjugating enzyme E2 N	0	4.049	19	2	152	17.1	6.57	2.41

P08574	Cytochrome c1, heme protein, mitochondrial	0	4.041	4	1	325	35.4	9	3.43
Q99615-1	DnaJ homolog subfamily C member 7	0	4.037	4	2	494	56.4	6.96	2.55
Q9UQ80	proliferation-associated protein 2G4	0	3.939	7	2	394	43.8	6.55	3.66
Q96QK1	Vacuolar protein sorting-associated protein 35	0	3.896	2	2	796	91.6	5.49	2.11
Q9NRW1	Ras-related protein Rab-6B	0	3.889	6	1	208	23.4	5.53	6.81
P35520-1	Cystathionine beta-synthase	0	3.883	3	2	551	60.5	6.65	2.82
P57088	Transmembrane protein 33	0	3.868	4	1	247	28	9.7	2.52
Q9BVC6	Transmembrane protein 109	0	3.862	5	1	243	26.2	10.48	2.9
P22102-1	trifunctional purine biosynthetic protein adenosine-3	0	3.844	2	1	1010	107.7	6.7	3.44
O14949	Cytochrome b-c1 complex subunit 8	0	3.841	16	1	82	9.9	10.08	2.91

P84103	Serine/arginine-rich splicing factor 3	0	3.834	14	2	164	19.3	11.65	5.06
P41091	eukaryotic translation initiation factor 2 subunit 3	0	3.802	7	2	472	51.1	8.4	5.02
Q15717	ELAV-like protein 1	0	3.8	6	2	326	36.1	9.17	4.78
P12270	Nucleoprotein TPR	0	3.786	2	2	2363	267.1	5.02	5.93
P27816-1	Microtubule-associated protein 4	0	3.768	2	2	1152	120.9	5.43	2.4
Q6P2Q9	Pre-mRNA-processing-splicing factor 8	0	3.733	1	1	2335	273.4	8.84	3.03
Q8N4V1	Membrane magnesium transporter 1	0	3.713	18	1	131	14.7	9.16	2.68
P61158	actin-related protein 3	0	3.679	3	1	418	47.3	5.88	3.09
Q6UN15-1	Pre-mRNA 3'-end-processing factor FIP1	0	3.663	2	1	594	66.5	5.59	2.6
Q9NZ01-1	Very-long-chain enoyl-CoA reductase	0	3.659	3	1	308	36	9.45	4.09
Q9NX58	Cell growth-regulating nucleolar protein	0	3.616	2	1	379	43.6	9.54	3.06

P43307	Translocon-associated protein subunit alpha	0	3.608	9	2	286	32.2	4.49	2.55
Q06210-1	glutamine--fructose-6-phosphate aminotransferase [isomerizing] 1	0	3.604	3	2	699	78.8	7.11	4.27
Q9H7D0-1	dedicator of cytokinesis protein 5	0	3.581	1	1	1870	215.2	7.96	3.95
Q9GZZ1-1	N-alpha-acetyltransferase 50	0	3.581	7	1	169	19.4	8.81	5.75
Q86V81	THO complex subunit 4	0	3.543	4	1	257	26.9	11.15	2.28
O75947-1	ATP synthase subunit d, mitochondrial	0	3.509	12	2	161	18.5	5.3	1.84
P51809	vesicle-associated membrane protein 7	0	3.487	5	1	220	24.9	8.6	2.44
Q9BQB6-1	vitamin K epoxide reductase complex subunit 1	0	3.465	8	1	163	18.2	9.36	3.05
Q14254	Flotillin-2	0	3.432	3	1	428	47	5.25	5.5
Q14157-1	Isoform 2 of Ubiquitin-associated	0	3.432	3	2	983	103.9	6.98	4.21

	protein 2-like								
Q8N5F7	NF-kappa-B-activating protein	0	3.408	7	1	415	47.1	10.11	3.75
P14406	Cytochrome c oxidase subunit 7A2, mitochondrial	0	3.404	16	1	83	9.4	9.76	4.23
Q9HC07	Transmembrane protein 165	0	3.383	8	1	324	34.9	7.02	3.28
P11310-1	medium-chain specific acyl-CoA dehydrogenase, mitochondrial	0	3.363	3	1	421	46.6	8.37	2.29
Q01813	ATP-dependent 6-phosphofructokinase, platelet type	0	3.331	2	1	784	85.5	7.55	2.86
P50750	Cyclin-dependent kinase 9	0	3.331	6	1	372	42.8	8.79	4.54
P10599-1	thioredoxin	0	3.308	12	1	105	11.7	4.92	4.79
Q08170	Serine/arginine-rich splicing factor 4	0	3.283	3	1	494	56.6	11.52	2.19
Q53H12	Acylglycerol kinase, mitochondrial	0	3.274	3	1	422	47.1	8.09	2.41

Q13451-1	Peptidyl-prolyl cis-trans isomerase FKBP5	0	3.272	4	2	457	51.2	5.9	2.2
Q9Y617-1	phosphoserine aminotransferase	0	3.261	8	2	370	40.4	7.66	2.69
Q5D862	Filaggrin-2	0	3.256	1	1	2391	247.9	8.31	2.23
Q8IWS0-1	PHD finger protein 6	0	3.238	4	1	365	41.3	8.68	2.51
Q16181	Septin-7	0	3.237	4	2	437	50.6	8.63	2.34
O43143	Pre-mRNA-splicing factor ATP-dependent RNA helicase DHX15	0	3.221	1	1	795	90.9	7.46	5.7
Q96ER3	protein saal1	0	3.198	3	1	474	53.5	4.5	4.95
Q8WXF1	Paraspeckle component 1	0	3.184	3	1	523	58.7	6.67	4.43
O43676	NADH dehydrogenase [ubiquinone] 1 beta subcomplex subunit 3	0	3.177	11	1	98	11.4	9.2	3.97
O75844	caax prenyl protease 1 homolog	0	3.177	2	1	475	54.8	7.49	4.68

P30086	phosphatidylethanolamine-binding protein 1	0	3.172	7	1	187	21	7.53	2.98
P82979	SAP domain-containing ribonucleoprotein	0	3.152	9	1	210	23.7	6.42	2.74
P14174	Macrophage Migration inhibitory factor	0	3.143	14	2	115	12.5	7.88	7.76
P50454	Serpin H1	0	3.097	8	2	418	46.4	8.69	2.8
P09132-1	Signal recognition particle 19 kDa protein	0	3.085	10	1	144	16.1	9.85	2.48
P56134	ATP synthase subunit f, mitochondrial	0	3.084	12	1	94	10.9	9.67	3.06
P15924-1	Desmoplakin	0	3.078	1	2	2871	331.6	6.81	1.69
Q86UP2-1	Kinectin	0	3.071	1	1	1357	156.2	5.64	2.32
O76003	glutaredoxin-3	0	3.071	4	1	335	37.4	5.39	2.32
P08579	U2 small nuclear ribonucleoprotein B"	0	3.061	5	1	225	25.5	9.72	6.03

Q99714-1	3-hydroxyacyl-CoA dehydrogenase type-2	0	3.057	9	1	261	26.9	7.78	2.73
Q00765	Receptor expression-enhancing protein 5	0	3.034	5	1	189	21.5	8.1	4.33
Q9BY77	Polymerase delta-interacting protein 3	0	3.017	3	1	421	46.1	9.99	4.52
Q9Y247	protein FAM50B	0	3.016	4	1	325	38.7	8.87	6.86
P28331-1	NADH-ubiquinone oxidoreductase 75 kDa subunit, mitochondrial	0	3.012	2	1	727	79.4	6.23	2.17
Q13404	Ubiquitin-conjugating enzyme E2 variant 1	0	3.006	7	1	147	16.5	7.93	5.06
P84243	histone H3.3	0	2.991	12	2	136	15.3	11.27	8.25
Q96HE7	ERO1-like protein alpha	0	2.987	3	1	468	54.4	5.68	2.44
Q9NR30-1	Nucleolar RNA helicase 2	0	2.947	2	1	783	87.3	9.28	2.4

Q9BUJ2-1	Heterogeneous nuclear ribonucleoprotein U-like protein 1	0	2.946	1	1	856	95.7	6.92	2.73
O43684	Mitotic checkpoint protein BUB3	0	2.942	6	2	328	37.1	6.84	1.99
P62191	26S proteasome regulatory subunit 4	0	2.932	4	1	440	49.2	6.21	3.55
O15355	Protein phosphatase 1G	0	2.932	2	1	546	59.2	4.36	3.22
Q3MHD2	Protein LSM12 homolog	0	2.922	7	1	195	21.7	7.74	0
P60763	Ras-related C3 botulinum toxin substrate 3	0	2.902	5	1	192	21.4	8.15	3.87
P38117	electron transfer flavoprotein subunit beta	0	2.882	7	1	255	27.8	8.1	2.13
Q16637	Survival motor neuron protein	0	2.861	7	2	294	31.8	6.55	3.95
P31689-1	DnaJ homolog subfamily A member 1	0	2.846	3	1	397	44.8	7.08	4.92
O94826	Mitochondrial import receptor subunit	0	2.846	3	2	608	67.4	7.12	4.69

	TOM70								
O95747	Serine/threonine-protein kinase OSR1	0	2.842	3	1	527	58	6.43	5.17
Q9UNL2	Translocon-associated protein subunit gamma	0	2.837	8	1	185	21.1	9.61	2.06
P23588	eukaryotic translation initiation factor 4B	0	2.821	3	1	611	69.1	5.73	3.63
O95793	double-stranded RNA-binding protein Staufen homolog 1	0	2.816	2	1	577	63.1	9.44	0
P23193	Transcription elongation factor A protein 1	0	2.777	4	1	301	33.9	8.38	5.07
Q04837	Single-stranded DNA-binding protein, mitochondrial	0	2.764	10	1	148	17.2	9.6	3.33
Q8IX12	Cell division cycle and apoptosis	0	2.758	1	1	1150	132.7	5.76	2.2

	regulator protein 1								
Q92734-1	Protein TFG	0	2.727	3	1	400	43.4	5.1	4.29
Q9Y5B9	FACT complex subunit SPT16	0	2.704	1	1	1047	119.8	5.66	1.61
Q52LJ0	Protein Fam98b	0	2.672	4	1	330	37.2	6.29	2.41
O75828	Carbonyl reductase [NADPH] 3	0	2.652	5	1	277	30.8	6.18	2.35
P61421	V-type proton ATPase subunit d 1	0	2.645	3	1	351	40.3	5	1.88
O14974	Protein phosphatase 1 regulatory subunit 12A	0	2.618	2	1	1030	115.2	5.4	2.16
O60684	Importin subunit alpha-7	0	2.614	3	1	536	60	4.98	1.86
P82650	28S ribosomal protein S22, mitochondrial	0	2.606	3	1	360	41.3	7.9	1.95
P13804-1	Electron transfer flavoprotein subunit alpha, mitochondrial	0	2.597	4	1	333	35.1	8.38	2.01

P11387	DNA topoisomerase 1	0	2.593	1	1	765	90.7	9.31	1.64
Q13620-1	Isoform 2 of Cullin-4B	0	2.59	1	1	895	102.2	7.94	2.61
P00505	Aspartate aminotransferase, mitochondrial	0	2.581	2	1	430	47.5	9.01	2.32
Q15637	Splicing factor 1	0	2.559	2	1	639	68.3	8.98	4.11
Q13813	Spectrin alpha chain, non-erythrocytic 1	0	2.52	1	2	2472	284.4	5.35	1.82
Q9Y450-1	HBS1-like protein	0	2.456	2	1	684	75.4	6.61	2.23
P36507	Dual specificity mitogen-activated protein kinase kinase 2	0	2.453	3	1	400	44.4	6.55	1.61
O00154	cytosolic acyl coenzyme A thioester hydrolase	0	2.448	4	1	380	41.8	8.54	0
Q6Y1H2	Very-long-chain (3R)-3-hydroxyacyl-CoA dehydratase 2	0	2.425	4	1	254	28.4	9.55	2.68

P18206	Vinculin	0	2.414	1	1	1134	123.7	5.66	2.12
Q12874	splicing factor 3a subunit 3	0	2.414	4	2	501	58.8	5.38	1.68
P60953	Cell division control protein 42 homolog	0	2.409	5	1	191	21.2	6.55	1.92
Q9BS26	Endoplasmic reticulum resident protein 44	0	2.405	3	1	406	46.9	5.26	1.84
P67870	Casein kinase II subunit beta	0	2.382	5	1	215	24.9	5.55	4.49
P56385	ATP synthase subunit e, mitochondrial	0	2.364	14	1	69	7.9	9.35	4.43
Q14258	E3 ubiquitin/ISG15 ligase TRIM25	0	2.358	2	1	630	70.9	8.09	1.97
P15954	Cytochrome c oxidase subunit 7C, mitochondrial	0	2.342	14	1	63	7.2	10.27	4.93
P29373	Cellular retinoic acid-binding protein 2	0	2.34	7	1	138	15.7	5.4	1.94
Q9UBX3	Mitochondrial dicarboxylate carrier	0	2.327	4	1	287	31.3	9.54	3.76
Q66PJ3-1	ADP-ribosylation factor-like protein 6-	0	2.313	3	1	421	44.9	10.93	2.49

	interacting protein 4								
O94776	Metastasis-associated protein MTA2	0	2.312	1	1	668	75	9.66	1.79
O60831	PRA1 family protein 2	0	2.28	6	1	178	19.2	9.19	2.3
P38159-1	RNA-binding motif protein, X chromosome	0	2.277	3	1	391	42.3	10.05	1.7
Q99873	protein arginine N-methyltransferase 1	0	2.273	4	1	361	41.5	5.43	2.55
Q8N1G4	Leucine-rich repeat-containing protein 47	0	2.272	2	1	583	63.4	8.28	2.33
Q15392	Delta(24)-sterol reductase	0	2.265	2	1	516	60.1	8.16	4.2
Q9BQG0	Myb-binding protein 1A	0	2.264	1	1	1328	148.8	9.28	2.09
O43399	Tumor protein D54	0	2.25	5	1	206	22.2	5.36	3.88
P63279	SUMO-conjugating enzyme ubc9	0	2.236	8	1	158	18	8.66	2.58
Q9NQC3	Reticulon-4	0	2.232	1	1	1192	129.9	4.5	4.49

Q8IY63-1	Angiomotin-like protein 1	0.002	2.209	2	1	956	106.5	7.11	5.88
P61619	Protein transport protein Sec61 subunit alpha isoform 1	0.002	2.205	2	1	476	52.2	8.06	5.29
Q15738	sterol-4-alpha-carboxylate 3-dehydrogenase, decarboxylating	0.002	2.202	3	1	373	41.9	8.06	2.15
Q92900	Regulator of nonsense transcripts 1	0.002	2.2	1	1	1129	124.3	6.61	2.11
Q9UHB6	LIM domain and actin-binding protein 1	0.002	2.198	2	1	759	85.2	6.84	0
Q9BPW8	Protein NipSnap homolog 1	0.002	2.184	4	1	284	33.3	9.31	2
P00403	Cytochrome c oxidase subunit 2	0.002	2.164	4	1	227	25.5	4.82	4.78
Q04760-1	lactoylglutathione lyase	0.002	2.137	5	1	184	20.8	5.31	0
P49736	DNA replication licensing factor mcm2	0.002	2.129	1	1	904	101.8	5.52	2.06
Q8WXE9-1	Stonin-2	0.002	2.127	1	1	905	101.1	5.39	6.54
P30084	Enoyl-CoA hydratase, mitochondrial	0.002	2.125	3	1	290	31.4	8.07	1.93

Q9BSD7	Cancer-related nucleoside-triphosphatase	0.002	2.117	6	1	190	20.7	9.54	1.88
Q7Z739	YTH domain-containing family protein 3	0.002	2.105	2	1	585	63.8	9.04	3.75
Q8N9B5-1	Junction-mediating and -regulatory protein	0.002	2.092	1	1	988	111.4	6.18	0
P84090	Enhancer of rudimentary homolog	0.002	2.08	16	1	104	12.3	5.92	2.16
P17980	26S proteasome regulatory subunit 6A	0.002	2.073	2	1	439	49.2	5.24	2.32
P46459	Vesicle-fusing ATPase	0.002	2.059	1	1	744	82.5	6.95	1.76
Q16822-1	Phosphoenolpyruvate carboxykinase [GTP], mitochondrial	0.002	2.032	2	1	640	70.7	7.62	1.73
O00410	Importin-5	0.002	2.004	1	1	1097	123.6	4.94	1.96
Q15427	Splicing factor 3b subunit 4	0.002	2.001	3	1	424	44.4	8.56	0

P46977	Dolichyl-diphosphooligosaccharide--protein glycosyltransferase subunit STT3A	0.002	1.992	1	1	705	80.5	8.07	3.88
P46063	ATP-dependent DNA helicase Q1	0.002	1.99	2	1	649	73.4	7.88	0
P35250-1	Replication factor C subunit 2	0.002	1.965	5	1	354	39.1	6.44	2.52
P61086	Ubiquitin-conjugating enzyme E2 K	0.002	1.953	6	1	200	22.4	5.44	1.61
P08758	annexin A5	0.002	1.952	3	1	320	35.9	5.05	2.19
Q9BSU3	N-alpha-acetyltransferase 11	0.002	1.952	5	1	229	26	5.17	1.76
Q13724-1	Mannosyl-oligosaccharide glucosidase	0.002	1.948	2	1	837	91.9	8.9	0
P51648	Fatty aldehyde dehydrogenase	0.002	1.948	2	1	485	54.8	7.88	0
Q15904	V-type proton ATPase subunit S1	0.002	1.945	2	1	470	52	6.14	4.47
O43865	S-adenosylhomocysteine hydrolase-like protein 1	0.002	1.943	2	1	530	58.9	6.89	1.84

P35249	replication factor C subunit 4	0.002	1.937	4	1	363	39.7	8.02	2.05
O75251	NADH dehydrogenase [ubiquinone] iron-sulfur protein 7, mitochondrial	0.002	1.935	7	1	213	23.5	9.99	1.63
Q13423	NAD(P) transhydrogenase, mitochondrial	0.002	1.935	1	1	1086	113.8	8.09	0
P06737-1	Glycogen phosphorylase, liver form	0.002	1.929	1	1	847	97.1	7.17	4.56
Q9Y696	Chloride intracellular channel protein 4	0.002	1.928	9	1	253	28.8	5.59	0
P61221	ATP-binding cassette sub-family E member 1	0.002	1.91	2	1	599	67.3	8.34	4.83
Q9UNE7-1	E3 ubiquitin-protein ligase CHIP	0.002	1.906	3	1	303	34.8	5.87	1.96
P46940	Ras GTPase-activating-like protein IQGAP1	0.002	1.9	1	1	1657	189.1	6.48	0
Q03252	Lamin-B2	0.002	1.88	2	1	620	69.9	5.59	1.79

P60468	protein transport protein Sec61 subunit beta	0.002	1.88	10	1	96	10	11.56	4.26
P43243	Matrin-3	0.002	1.84	2	1	847	94.6	6.25	0
Q15363	Transmembrane emp24 domain-containing protein 2	0.002	1.83	4	1	201	22.7	5.17	1.64
Q9BWJ5	Splicing factor 3B subunit 5	0.002	1.829	13	1	86	10.1	6.35	1.89
P07477	Trypsin-1	0.002	1.826	3	1	247	26.5	6.51	0
P40616	ADP-ribosylation factor-like protein 1	0.002	1.811	5	1	181	20.4	5.72	2.2
P35637-1	RNA-binding protein FUS	0.002	1.794	3	1	526	53.4	9.36	2.31
P31350-1	ribonucleoside-diphosphate reductase subunit M2	0.002	1.783	3	1	389	44.8	5.38	1.97
Q12907	Vesicular integral-membrane protein VIP36	0.002	1.783	3	1	356	40.2	6.95	2.58

P09661	U2 small nuclear ribonucleoprotein A'	0.002	1.781	4	1	255	28.4	8.62	1.82
Q8TC12	Retinol dehydrogenase 11	0.002	1.772	3	1	318	35.4	8.82	4.31
Q9Y4P3	Transducin beta-like protein 2	0.002	1.769	3	1	447	49.8	9.44	0
Q9Y6G9	Cytoplasmic dynein 1 light intermediate chain 1	0.003	1.755	3	1	523	56.5	6.42	2.03
O14818-1	Proteasome subunit alpha type-7	0.003	1.754	4	1	248	27.9	8.46	4.13
P11766	alcohol dehydrogenase class-3	0.003	1.745	2	1	374	39.7	7.49	1.97
P07384	Calpain-1 catalytic subunit	0.003	1.743	1	1	714	81.8	5.67	0
P08243-1	Asparagine synthetase [glutamine-hydrolyzing]	0.003	1.738	1	1	561	64.3	6.86	2.19
O60870	DNA/RNA-binding protein KIN17	0.003	1.729	2	1	393	45.3	8.95	1.76
P26639-1	Threonine--tRNA ligase, cytoplasmic	0.003	1.723	2	1	723	83.4	6.67	0
O75845	lathosterol oxidase	0.003	1.721	3	1	299	35.3	8.07	0

Q06787	synaptic functional regulator FMR1	0.003	1.699	1	1	632	71.1	7.42	0
P52209	6-phosphogluconate dehydrogenase, decarboxylating	0.003	1.696	2	1	483	53.1	7.23	0
P09234	U1 small nuclear ribonucleoprotein C	0.003	1.694	6	1	159	17.4	9.67	4.82
P01624	Immunoglobulin kappa variable 3-15	0.003	1.692	8	1	115	12.5	5.19	3.64
Q9UJV9	Probable ATP-dependent RNA helicase DDX41	0.003	1.69	2	1	622	69.8	6.84	1.89
P07741-1	Adenine phosphoribosyltransferase	0.004	1.665	4	1	180	19.6	6.02	1.86
Q9UNX4	WD repeat-containing protein 3	0.004	1.651	1	1	943	106	6.64	0
P48556	26S proteasome non-ATPase regulatory subunit 8	0.004	1.647	2	1	350	39.6	9.7	2.2
P30048	Thioredoxin-dependent peroxide reductase, mitochondrial	0.004	1.632	4	1	256	27.7	7.78	2.17

Q08J23	tRNA (Cytosine(34)-C(5))-methyltransferase	0.004	1.632	1	1	767	86.4	6.77	1.77
P13473-1	Lysosome-associated membrane glycoprotein 2	0.004	1.592	2	1	410	44.9	5.63	1.97
Q9UKA9-1	Polypyrimidine tract-binding protein 2	0.004	1.592	2	1	531	57.5	8.66	2.25
Q96PK6-1	RNA-binding protein 14	0.004	1.585	2	1	669	69.4	9.67	2
O75533-1	splicing factor 3B subunit 1	0.004	1.584	1	1	1304	145.7	7.09	1.61
Q92520	Protein FAM3C	0.004	1.581	7	1	227	24.7	8.29	1.8
Q9NU22	Midasin	0.004	1.566	0	1	5596	632.4	5.68	1.64
Q16527	Cysteine and glycine-rich protein 2	0.004	1.554	8	1	193	20.9	8.62	1.85
Q9NVP1	ATP-dependent RNA helicase DDX18	0.004	1.545	2	1	670	75.4	9.5	1.68
Q08752	peptidyl-prolyl cis-trans isomerase D	0.004	1.541	2	1	370	40.7	7.21	1.86
P10768	S-formylglutathione hydrolase	0.004	1.539	8	1	282	31.4	7.02	2.72

P36957	Dihydrolipoyllysine-residue succinyltransferase component of 2-oxoglutarate dehydrogenase complex, mitochondrial	0.004	1.537	3	1	453	48.7	8.95	3.93
Q9BRX2	Protein pelota homolog	0.004	1.534	2	1	385	43.3	6.34	1.68
P14678-1	Small nuclear ribonucleoprotein-associated proteins B and B'	0.004	1.522	3	1	240	24.6	11.19	1.94
Q04724	Transducin-like enhancer protein 1	0.004	1.514	1	1	770	83.1	7.24	1.97
Q9Y2A7-1	Nck-associated protein 1	0.004	1.513	1	1	1128	128.7	6.62	1.62
Q15645	Pachytene checkpoint protein 2 homolog	0.004	1.512	3	1	432	48.5	6.09	0
P17858-1	ATP-dependent 6-phosphofructokinase, liver type	0.004	1.51	1	1	780	85	7.5	4.24
Q99459	Cell division cycle 5-like protein	0.004	1.506	2	1	802	92.2	8.18	1.69

Q9H3K6-1	bola-like protein 2	0.004	1.498	8	1	86	10.1	6.52	1.7
P78417	Glutathione S-transferase omega-1	0.004	1.494	4	1	241	27.5	6.6	1.9
Q13155	aminoacyl tRNA synthase complex-interacting multifunctional protein 2	0.004	1.49	2	1	320	35.3	8.22	1.62
Q9Y285	Phenylalanine--tRNA ligase alpha subunit	0.004	1.489	3	1	508	57.5	7.8	2.41
O75390	citrate synthase, mitochondrial	0.004	1.475	2	1	466	51.7	8.32	2.14
Q9NUP9	Protein lin-7 homolog C	0.004	1.471	6	1	197	21.8	8.43	2.02
Q969M3	Protein YIPF5	0.004	1.467	4	1	257	28	4.36	0
P61289-1	Proteasome activator complex subunit 3	0.004	1.465	5	1	254	29.5	5.95	0
Q9NZP5	Olfactory receptor 5AC2	0.004	1.455	2	1	309	35.3	8.94	4.07
Q5VTU8	ATP synthase subunit epsilon-like protein, mitochondrial	0.004	1.454	14	1	51	5.8	10.14	0

O43491-1	band 4.1-like protein 2	0.004	1.444	1	1	1005	112.5	5.44	2.03
P35241-1	radixin	0.004	1.43	1	1	583	68.5	6.37	1.74
P27695	DNA-(apurinic or apyrimidinic site) lyase	0.004	1.42	5	1	318	35.5	8.12	2.02
Q99536	Synaptic vesicle membrane protein VAT-1 homolog	0.011	1.385	2	1	393	41.9	6.29	0
Q9UQ35	serine/arginine repetitive matrix protein 2	0.011	1.371	1	1	2752	299.4	12.06	2.03
P08621-1	U1 small nuclear ribonucleoprotein 70 kDa	0.011	1.365	4	1	437	51.5	9.94	0
O00469-1	procollagen-lysine,2-oxoglutarate 5-dioxygenase 2	0.011	1.363	2	1	737	84.6	6.71	2.28
Q99805	Transmembrane 9 superfamily member	0.011	1.363	2	1	663	75.7	7.44	2.23

	2								
P28288	ATP-binding cassette sub-family D member 3	0.011	1.354	1	1	659	75.4	9.36	0
Q8NEZ4	Histone-lysine N-methyltransferase 2C	0.011	1.35	0	1	4911	541	6.49	0
Q9Y4W6	AFG3-like protein 2	0.012	1.339	1	1	797	88.5	8.66	0
O95816	BAG family molecular chaperone regulator 2	0.012	1.333	4	1	211	23.8	6.7	1.97
P27694	Replication protein A 70 kDa DNA-binding subunit	0.012	1.322	2	1	616	68.1	7.21	0
P10644	cAMP-dependent protein kinase type I-alpha regulatory subunit	0.013	1.316	4	1	381	43	5.35	0
P40926	Malate dehydrogenase, mitochondrial	0.013	1.313	2	1	338	35.5	8.68	3.49
Q6UW56	All-trans retinoic acid-induced	0.013	1.311	3	1	229	24.7	7.25	0

	differentiation factor								
Q8N9Q2	protein SREK1IP1	0.013	1.306	5	1	155	18.2	9.85	4.03
Q494V2-1	Cilia- and flagella-associated protein 100	0.013	1.302	1	1	611	71.1	7.11	3.51
P11166	Solute carrier family 2, facilitated glucose transporter member 1	0.013	1.288	2	1	492	54	8.72	0
O75179-1	Ankyrin repeat domain-containing protein 17	0.013	1.286	0	1	2603	274.1	6.52	0
Q7Z3B4	Nucleoporin p54	0.013	1.273	3	1	507	55.4	7.02	0
Q9HB71	Calcyclin-binding protein	0.013	1.27	5	1	228	26.2	8.25	0
P04049	RAF proto-oncogene serine/threonine-protein kinase	0.014	1.246	2	1	648	73	9.2	1.84
P55884	Eukaryotic translation initiation factor 3	0.016	1.238	1	1	814	92.4	5	2.16

	subunit B								
Q01469	Fatty acid-binding protein, epidermal	0.015	1.218	7	1	135	15.2	7.01	1.72
Q7L1Q6-1	Basic leucine zipper and W2 domain-containing protein 1	0.015	1.209	2	1	419	48	5.92	1.84
P11233	Ras-related protein Ral-A	0.015	1.198	3	1	206	23.6	7.11	3.52
P01614	Immunoglobulin kappa variable 2D-40	0.015	1.192	6	1	121	13.3	4.61	2.19
Q8WTS6	Histone-lysine N-methyltransferase SETD7	0.015	1.185	2	1	366	40.7	4.63	0
Q8TBE7	Solute carrier family 35 member G2	0.015	1.182	3	1	412	46.4	7.34	0
Q15003	Condensin complex subunit 2	0.015	1.176	1	1	741	82.5	5.06	0
O75955	Flotillin-1	0.015	1.174	2	1	427	47.3	7.49	0
P30536	translocator protein	0.015	1.173	5	1	169	18.8	9.36	2.11
P53701	Cytochrome c-type heme lyase	0.015	1.163	3	1	268	30.6	6.68	0

P0C0L4-1	Complement C4-A	0.015	1.156	1	1	1744	192.7	7.08	0
O94808	Glutamine--fructose-6-phosphate aminotransferase [isomerizing] 2	0.015	1.156	1	1	682	76.9	7.37	7.67
Q9UBM7	7-dehydrocholesterol reductase	0.016	1.135	2	1	475	54.5	8.7	1.97
Q15120-1	[Pyruvate dehydrogenase (acetyl-transferring)] kinase isozyme 3, mitochondrial	0.016	1.134	4	1	406	46.9	8.37	0
Q66LE6	Serine/threonine-protein phosphatase 2A 55 kDa regulatory subunit B delta isoform	0.016	1.13	1	1	453	52	6.39	0
O60637	Tetraspanin-3	0.016	1.129	3	1	253	28	5.81	3.95
Q15019	septin-2	0.016	1.124	7	1	361	41.5	6.6	0
Q9BTV4	Transmembrane protein 43	0.016	1.123	3	1	400	44.8	8.13	1.76

Q9H3P2-1	Negative elongation factor A	0.016	1.113	3	1	528	57.2	9.03	1.66
Q13428-1	Treacle protein	0.017	1.106	1	1	1488	152	9.04	1.65
Q9NWB6	Arginine and glutamate-rich protein 1	0.018	1.099	3	1	273	33.2	10.35	1.87
P13995	Bifunctional methylenetetrahydrofolate dehydrogenase/cyclohydrolase, mitochondrial	0.019	1.082	3	1	350	37.9	8.73	2.19
Q9Y3A6-1	Transmembrane emp24 domain-containing protein 5	0.019	1.072	5	1	229	26	4.84	0
O00273-1	DNA fragmentation factor subunit alpha	0.019	1.071	4	1	331	36.5	4.79	0
O60942-1	mRNA-capping enzyme	0.019	1.069	2	1	597	68.5	8.13	0
Q86XP3-1	ATP-dependent RNA helicase DDX42	0.019	1.061	1	1	938	102.9	7.02	0
P28838	cytosol aminopeptidase	0.019	1.053	2	1	519	56.1	7.93	0
Q56UQ5	TPT1-like protein	0.019	1.052	9	1	140	15.7	6.19	0

Q969N2	GPI transamidase component PIG-T	0.019	1.045	2	1	578	65.7	8.38	1.81
Q9BZH6	WD repeat-containing protein 11	0.019	1.042	1	1	1224	136.6	6.92	2.06
P05198	Eukaryotic translation initiation factor 2 subunit 1	0.019	1.033	5	1	315	36.1	5.08	0
Q13642	Four and a half LIM domains protein 1	0.019	1.033	2	1	323	36.2	8.97	1.86
Q9Y394	Dehydrogenase/reductase SDR family member 7	0.019	1.032	6	1	339	38.3	8.32	0
Q14966-1	Zinc finger protein 638	0.019	1.026	0	1	1978	220.5	6.38	4.81
Q6UB35-1	Monofunctional C1-tetrahydrofolate synthase, mitochondrial	0.019	1.024	1	1	978	105.7	8.06	0
P67812	Signal peptidase complex catalytic subunit SEC11A	0.019	1.013	4	1	179	20.6	9.48	0
Q14980-1	nuclear mitotic apparatus protein 1	0.019	1.007	1	1	2115	238.1	5.78	0

P43308	Translocon-associated protein subunit beta	0.02	1	3	1	183	20.1	8.35	0
Q9UIL1-1	Short coiled-coil protein	0.02	0.991	16	1	159	18	8.85	0
Q9BQ52-1	Zinc phosphodiesterase ELAC protein 2	0.02	0.983	1	1	826	92.2	7.9	1.77
O95399	Urotensin-2	0.02	0.981	6	1	124	14.3	7.8	0
Q9HA64	Ketosamine-3-kinase	0.022	0.981	5	1	309	34.4	7.33	0
O00506	serine/threonine-protein kinase 25	0.022	0.969	4	1	426	48.1	6.74	0
Q16654	[Pyruvate dehydrogenase (acetyl-transferring)] kinase isozyme 4, mitochondrial	0.022	0.964	2	1	411	46.4	6.65	0
Q6UWP7-1	Lysocardiolipin acyltransferase 1	0.022	0.962	5	1	414	48.9	8.62	0
Q9H3R5	Centromere protein H	0.022	0.959	7	1	247	28.5	5.29	0
P68400-2	Isoform 2 of Casein kinase II subunit	0.025	0.945	2	1	255	29.2	6.71	0

	alpha								
P62304	small nuclear ribonucleoprotein E	0.025	0.944	12	1	92	10.8	9.44	1.66
P01701	immunoglobulin lambda variable 1-51	0.025	0.937	7	1	117	12.2	7.03	0
Q9H159-1	cadherin-19	0.025	0.933	1	1	772	86.9	4.73	1.61
P13611	Versican core protein	0.027	0.912	0	1	3396	372.6	4.51	1.61
A8MVM7	Putative uncharacterized protein ENSP00000382790	0.027	0.907	1	1	634	73.4	9.79	0
O43617-1	Trafficking protein particle complex subunit 3	0.031	0.904	4	1	180	20.3	4.96	0
Q9BZE4	Nucleolar GTP-binding protein 1	0.031	0.901	1	1	634	73.9	9.5	0
P56270-1	Myc-associated zinc finger protein	0.036	0.896	3	1	477	48.6	8.95	0
O00767	acyl-CoA desaturase	0.036	0.893	2	1	359	41.5	9	0
Q86UK5-1	Limbin	0.036	0.891	1	1	1308	147.9	6.96	1.64

O60493-1	sorting nexin-3	0.036	0.891	7	1	162	18.8	8.66	0
P11717	Cation-independent mannose-6-phosphate receptor	0.036	0.889	0	1	2491	274.2	5.94	1.91
Q96DC9	Ubiquitin thioesterase OTUB2	0.036	0.888	3	1	234	27.2	6.23	2.15
Q9NYK1	Toll-like receptor 7	0.038	0.886	1	1	1049	120.8	8.21	0
Q5TA89	Transcription factor HES-5	0.039	0.881	5	1	166	18.2	9.48	2.07
Q8N2S1-3	Isoform 3 of Latent-transforming growth factor beta-binding protein 4	0.039	0.877	1	1	1587	169.4	5.29	0
Q96K17-1	transcription factor BTF3 homolog 4	0.039	0.872	4	1	158	17.3	6.35	0
P08183	multidrug resistance protein 1	0.039	0.871	1	1	1280	141.4	9	1.66
O60841	Eukaryotic translation initiation factor 5B	0.039	0.871	1	1	1220	138.7	5.49	0
E5RG02	Putative serine protease 46	0.039	0.868	3	1	174	19.3	9.1	0

Q6AHZ1-1	Zinc finger protein 518A	0.039	0.867	0	1	1483	166.7	9.28	0
A0PJE2-1	Dehydrogenase/reductase SDR family member 12	0.04	0.865	4	1	317	35.1	7.34	0
P43490	nicotinamide phosphoribosyltransferase	0.04	0.865	1	1	491	55.5	7.15	1.66
Q15696	U2 small nuclear ribonucleoprotein auxiliary factor 35 kDa subunit-related protein 2	0.04	0.861	1	1	482	58	9.72	0
Q6UVM3	potassium channel subfamily T member 2	0.04	0.858	1	1	1135	130.4	7.28	0
Q05397	Focal adhesion kinase 1	0.04	0.857	1	1	1052	119.2	6.62	2.14
Q15022	Polycomb protein suz12	0.04	0.851	1	1	739	83	8.81	0
Q15813	Tubulin-specific chaperone E	0.04	0.848	2	1	527	59.3	6.76	0
Q8WZ42	Titin	0.04	0.844	0	1	34350	3813.7	6.35	4.3

P10155-1	60 kDa SS-A/Ro ribonucleoprotein	0.04	0.838	2	1	538	60.6	8.03	0
Q8NHP8	Putative phospholipase B-like 2	0.04	0.834	1	1	589	65.4	6.8	0
Q15388	Mitochondrial import receptor subunit TOM20 homolog	0.04	0.833	9	1	145	16.3	8.6	2.4
Q1KMD3	heterogeneous nuclear ribonucleoprotein U-like protein 2	0.042	0.824	2	1	747	85.1	4.91	0
Q8IZJ3	C3 and PZP-like alpha-2-macroglobulin domain-containing protein 8	0.043	0.824	0	1	1885	206.6	6.42	0
P08754	Guanine nucleotide-binding protein G(k) subunit alpha	0.045	0.82	4	1	354	40.5	5.69	0

Supplementary Table 6 LC-MS/MS results of the IgG group

Accession	Description	Exp. q-value	Sum PEP Score	Coverage (%)	Unique Peptides	AAs	MW (kDa)	calc. pI	Score
P35579-1	Myosin-9	0	609.219	57	94	1960	226.4	5.6	844.55
P35580	Myosin-10	0	352.222	43	61	1976	228.9	5.54	473.12
P60709	Actin, cytoplasmic 1	0	220.899	66	9	375	41.7	5.48	311.71
P04406-1	glyceraldehyde-3-phosphate dehydrogenase	0	208.062	73	19	335	36	8.46	246.8
P14618	Pyruvate kinase PKM	0	188.371	64	30	531	57.9	7.84	234.41
P68371	Tubulin beta-4B chain	0	155.742	61	4	445	49.8	4.89	224.19
P04264	Keratin, type II cytoskeletal 1	0	155.063	60	26	644	66	8.12	223.62
P07437	tubulin beta chain	0	152.307	61	3	444	49.6	4.89	222.35
P38646	Stress-70 protein, mitochondrial	0	143.938	51	30	679	73.6	6.16	189.92
P13645	Keratin, type I cytoskeletal 10	0	142.736	47	23	584	58.8	5.21	220.44
P08238	Heat shock protein HSP 90-beta	0	133.346	42	18	724	83.2	5.03	197.91
P35908	Keratin, type II cytoskeletal 2	0	121.51	54	22	639	65.4	8	186.86

	epidermal								
P68104	Elongation factor 1-alpha 1	0	115.859	51	9	462	50.1	9.01	177.16
P68363	Tubulin alpha-1B chain	0	108.179	52	3	451	50.1	5.06	158.43
P07900	Heat shock protein HSP 90-alpha	0	107.542	33	14	732	84.6	5.02	153.39
Q13885	Tubulin beta-2A chain	0	101.075	42	1	445	49.9	4.89	156.61
Q9BVA1	Tubulin beta-2B chain	0	99.464	42	1	445	49.9	4.89	155.28
P11142-1	Heat shock cognate 71 kDa protein	0	96.491	36	17	646	70.9	5.52	137.15
P34896-1	Serine hydroxymethyltransferase, cytosolic	0	91.934	42	14	483	53	7.71	114.51
P13639	Elongation factor 2	0	91.097	29	21	858	95.3	6.83	120.47
P10809	60 kDa heat shock protein, mitochondrial	0	90.304	39	18	573	61	5.87	122.36
P0DMV8	heat shock 70 kDa protein 1A	0	87.612	41	20	641	70	5.66	112.62
P08670	Vimentin	0	85.961	53	24	466	53.6	5.12	126.48
Q4VCS5	Angiomotin	0	85.133	26	25	1084	118	7.64	116.85
P11940-1	Polyadenylate-binding protein 1	0	84.181	32	14	636	70.6	9.5	103.18
P35527	Keratin, type I cytoskeletal 9	0	78.322	33	17	623	62	5.24	115.97

P11021	78 kDa glucose-regulated protein	0	77.821	41	20	654	72.3	5.16	96.71
P60842	Eukaryotic initiation factor 4A-I	0	75.723	35	11	406	46.1	5.48	99.18
Q7Z406-1	myosin-14	0	73.488	13	14	1995	227.7	5.6	102.43
Q9BQE3	Tubulin alpha-1C chain	0	69.321	46	1	449	49.9	5.1	103.31
P12268	inosine-5'-monophosphate dehydrogenase 2	0	68.64	30	13	514	55.8	6.9	91.97
P68133	Actin, alpha skeletal muscle	0	68.554	30	1	377	42	5.39	130.45
P52272	Heterogeneous nuclear ribonucleoprotein M	0	62.17	26	18	730	77.5	8.7	92.43
P26641	elongation factor 1-gamma	0	60.674	28	12	437	50.1	6.67	74.62
P06733-1	alpha-enolase	0	60.578	38	14	434	47.1	7.39	83.12
P61978	Heterogeneous nuclear ribonucleoprotein K	0	58.7	30	11	463	50.9	5.54	90.83
O43175	D-3-phosphoglycerate dehydrogenase	0	58.041	23	10	533	56.6	6.71	79.01
P78371-1	T-complex protein 1 subunit beta	0	54.53	29	12	535	57.5	6.46	69.05
P31943	Heterogeneous nuclear	0	51.559	34	8	449	49.2	6.3	70.72

	ribonucleoprotein H								
P09874	Poly [ADP-ribose] polymerase 1	0	51.35	14	14	1014	113	8.88	61.44
P05023	Sodium/potassium-transporting ATPase subunit alpha-1	0	49.707	16	12	1023	112.8	5.49	50.27
P01834	immunoglobulin kappa constant	0	49.413	86	7	107	11.8	6.52	54.26
Q16891	MICOS complex subunit Mic60	0	48.746	21	15	758	83.6	6.48	48.08
P04075	fructose-bisphosphate aldolase A	0	47.928	46	13	364	39.4	8.09	68.61
P09651-1	Heterogeneous nuclear ribonucleoprotein A1	0	47.799	32	9	372	38.7	9.13	70.34
P25705-1	ATP synthase subunit alpha, mitochondrial	0	47.357	27	12	553	59.7	9.13	75.95
P11586	C-1-tetrahydrofolate synthase, cytoplasmic	0	47.161	16	12	935	101.5	7.3	53.82
P50990	T-complex protein 1 subunit theta	0	46.922	28	15	548	59.6	5.6	70.98
P49368-1	T-complex protein 1 subunit gamma	0	46.059	31	13	545	60.5	6.49	59.36
O14950	Myosin regulatory light chain 12B	0	44.838	66	9	172	19.8	4.84	70.89
P12277	Creatine kinase B-type	0	44.563	31	8	381	42.6	5.59	67.85

P36578	60S ribosomal protein L4	0	44.376	28	10	427	47.7	11.06	54.44
Q05639	Elongation factor 1-alpha 2	0	44.31	24	1	463	50.4	9.03	80.87
P12956	X-ray repair cross-complementing protein 6	0	43.816	20	9	609	69.8	6.64	47.02
Q00839	Heterogeneous nuclear ribonucleoprotein U	0	43.514	19	1	825	90.5	6	62.22
Q00839-2	Isoform Short of Heterogeneous nuclear ribonucleoprotein U	0	42.652	19	1	806	88.9	5.78	62.22
Q99623	Prohibitin-2	0	41.209	40	11	299	33.3	9.83	52.35
P18621	60S ribosomal protein L17	0	41.141	46	8	184	21.4	10.17	56.13
P07195	L-lactate dehydrogenase B chain	0	41.076	30	9	334	36.6	6.05	54.23
P14625	Endoplasmin	0	41.024	18	12	803	92.4	4.84	44.61
P61247	40S ribosomal protein S3a	0	39.905	44	11	264	29.9	9.73	62.6
P23528	Cofilin-1	0	37.64	53	8	166	18.5	8.09	60.44
P05387	60S acidic ribosomal protein P2	0	36.818	70	5	115	11.7	4.54	47.77
P23396-1	40S ribosomal protein S3	0	36.367	56	11	243	26.7	9.66	61.45
P22314	Ubiquitin-like modifier-activating	0	35.733	11	8	1058	117.8	5.76	35.95

	enzyme 1								
P22626	heterogeneous nuclear ribonucleoproteins A2/B1	0	35.34	36	10	353	37.4	8.95	51.7
Q02878	60S ribosomal protein L6	0	35.258	31	12	288	32.7	10.58	55.33
P13010	X-ray repair cross-complementing protein 5	0	35.107	14	9	732	82.7	5.81	38.49
P49327	Fatty acid synthase	0	34.1	6	9	2511	273.3	6.44	33.99
P19338	Nucleolin	0	34.022	20	17	710	76.6	4.7	50.64
P00558	phosphoglycerate kinase 1	0	32.931	22	8	417	44.6	8.1	40.8
Q92841-1	Isoform 2 of Probable ATP-dependent RNA helicase DDX17	0	31.942	17	7	650	72.3	8.59	45.32
P30050-1	60S ribosomal protein L12	0	31.174	54	7	165	17.8	9.42	41.99
P06576	ATP synthase subunit beta, mitochondrial	0	31.016	19	7	529	56.5	5.4	41.71
Q15366-3	Isoform 3 of Poly(rC)-binding protein 2	0	30.796	34	6	362	38.2	6.79	36.58
P32969	60S ribosomal protein L9	0	30.707	41	7	192	21.9	9.95	30.09

Q92945	Far upstream element-binding protein 2	0	30.586	20	9	711	73.1	7.3	32.24
P63244	Receptor of activated protein C kinase 1	0	30.412	24	6	317	35.1	7.69	31.18
P49411	elongation factor Tu, mitochondrial	0	30.34	19	6	452	49.5	7.61	31.65
P14868	Aspartate--tRNA ligase, cytoplasmic	0	30.312	18	8	501	57.1	6.55	35.28
P17987	T-complex protein 1 subunit alpha	0	30.073	26	11	556	60.3	6.11	33.5
P13647	keratin, type II cytoskeletal 5	0	30.058	18	5	590	62.3	7.74	46.01
P22234	multifunctional protein ADE2	0	29.954	23	8	425	47	7.23	34.75
P38919	Eukaryotic initiation factor 4A-III	0	29.911	21	5	411	46.8	6.73	29.85
Q9NVI7-2	Isoform 2 of ATPase family AAA domain-containing protein 3A	0	29.838	23	12	586	66.2	9.25	38.72
P23246-1	splicing factor, proline- and glutamine-rich	0	29.77	13	7	707	76.1	9.44	37.01
B9A064-1	Immunoglobulin lambda-like polypeptide 5	0	29.463	31	3	214	23	8.84	39.06
P62937	peptidyl-prolyl cis-trans isomerase A	0	29.416	41	9	165	18	7.81	38.34

P62424	60S ribosomal protein L7a	0	29.175	33	7	266	30	10.61	47
P40227-1	T-complex protein 1 subunit zeta	0	28.877	18	9	531	58	6.68	36
Q9NZI8	Insulin-like growth factor 2 mRNA-binding protein 1	0	28.811	19	10	577	63.4	9.2	30.87
P62701	40S ribosomal protein S4, X isoform	0	28.711	27	9	263	29.6	10.15	45.5
P07910-1	Heterogeneous nuclear ribonucleoproteins C1/C2	0	28.691	24	8	306	33.7	5.08	35.03
P15924-1	Desmoplakin	0	28.588	4	11	2871	331.6	6.81	32.12
P31948	stress-induced-phosphoprotein 1	0	28.47	18	10	543	62.6	6.8	30.2
O94832	Unconventional myosin-Id	0	28.05	10	10	1006	116.1	9.39	27.39
P50991	T-complex protein 1 subunit delta	0	27.637	17	8	539	57.9	7.83	36.33
P05141	ADP/ATP translocase 2	0	27.578	35	4	298	32.8	9.69	49.86
P12236	ADP/ATP translocase 3	0	27.443	31	3	298	32.8	9.74	49.42
P62241	40S ribosomal protein S8	0	27.256	38	7	208	24.2	10.32	42.19
P51991-1	Heterogeneous nuclear ribonucleoprotein A3	0	27.217	18	4	378	39.6	9.01	20.85
P0DOY2	immunoglobulin lambda constant 2	0	27.007	56	2	106	11.3	7.24	45.92

P35232	Prohibitin	0	26.934	32	7	272	29.8	5.76	36.02
P27635	60S ribosomal protein L10	0	26.864	31	9	214	24.6	10.08	33.79
Q9Y597	BTB/POZ domain-containing protein KCTD3	0	26.696	13	8	815	88.9	7.03	30.32
P78527	DNA-dependent protein kinase catalytic subunit	0	26.592	3	11	4128	468.8	7.12	26.71
P13797	Plastin-3	0	26.463	13	6	630	70.8	5.6	29.02
P48643	T-complex protein 1 subunit epsilon	0	26.339	14	7	541	59.6	5.66	34.36
P12004	proliferating cell nuclear antigen	0	26.241	29	7	261	28.8	4.69	35.17
P52597	Heterogeneous nuclear ribonucleoprotein F	0	26.209	16	3	415	45.6	5.58	24.91
Q06830	peroxiredoxin-1	0	26.178	33	6	199	22.1	8.13	40.15
P06748	Nucleophosmin	0	26.095	25	8	294	32.6	4.78	52.13
P39023	60S ribosomal protein L3	0	26.058	24	9	403	46.1	10.18	31.75
Q13310	Polyadenylate-binding protein 4	0	25.979	16	5	644	70.7	9.26	34.55
P04843	Dolichyl-diphosphooligosaccharide--protein glycosyltransferase subunit 1	0	25.902	17	8	607	68.5	6.38	30.02

Q15365	Poly(RC)-binding protein 1	0	25.529	22	3	356	37.5	7.09	31.06
P51659-1	peroxisomal multifunctional enzyme type 2	0	25.208	11	6	736	79.6	8.84	30.67
P15880	40S ribosomal protein S2	0	25.184	27	8	293	31.3	10.24	39.08
P60660	Myosin light polypeptide 6	0	25.113	42	6	151	16.9	4.65	44.44
Q16643	drebrin	0	25.109	14	6	649	71.4	4.45	33.5
Q14204	Cytoplasmic dynein 1 heavy chain 1	0	25.083	2	9	4646	532.1	6.4	27.39
P02533	Keratin, type I cytoskeletal 14	0	25.02	23	7	472	51.5	5.16	25.28
P04259	keratin, type II cytoskeletal 6B	0	24.668	15	1	564	60	8	46.71
P62888	60S ribosomal protein L30	0	24.331	59	5	115	12.8	9.63	22.42
Q12906-1	Interleukin enhancer-binding factor 3	0	24.3	9	6	894	95.3	8.76	23.74
Q12931	heat shock protein 75 kDa, mitochondrial	0	23.565	10	6	704	80.1	8.21	20.75
P67809	Nuclease-sensitive element-binding protein 1	0	23.065	29	4	324	35.9	9.88	23.37
Q8NC51-1	Plasminogen activator inhibitor 1 RNA-binding protein	0	22.812	18	6	408	44.9	8.65	30.22

P62280	40S ribosomal protein S11	0	22.76	39	6	158	18.4	10.3	37.92
P26373-1	60S ribosomal protein L13	0	22.275	32	7	211	24.2	11.65	39.71
P62826	GTP-binding nuclear protein RAN	0	22.203	31	7	216	24.4	7.49	37.44
P27824	Calnexin	0	22.108	8	4	592	67.5	4.6	26.32
O60506	Heterogeneous nuclear ribonucleoprotein Q	0	22.07	12	2	623	69.6	8.59	26.36
P60866	40S ribosomal protein S20	0	21.852	23	3	119	13.4	9.94	26.15
Q99832	T-complex protein 1 subunit eta	0	21.849	16	8	543	59.3	7.65	19.75
O43390-1	heterogeneous nuclear ribonucleoprotein r	0	21.697	15	4	633	70.9	8.13	17.21
P78347	General transcription factor II-I	0	21.625	9	7	998	112.3	6.39	25.88
Q13151	Heterogeneous nuclear ribonucleoprotein A0	0	21.512	24	5	305	30.8	9.29	19.21
Q15233	Non-POU domain-containing octamer-binding protein	0	21.377	16	7	471	54.2	8.95	30.04
Q00610-1	Clathrin heavy chain 1	0	21.271	4	5	1675	191.5	5.69	14.97
P43243	Matrin-3	0	21.155	9	5	847	94.6	6.25	16.23

Q13263	Transcription intermediary factor 1-beta	0	20.871	10	8	835	88.5	5.77	16.43
P62277	40S ribosomal protein S13	0	20.369	36	6	151	17.2	10.54	29.2
P62906	60S ribosomal protein L10A	0	20.029	25	6	217	24.8	9.94	29.18
P00338-1	L-lactate dehydrogenase A chain	0	20.019	22	6	332	36.7	8.27	29.64
Q9Y265	RuvB-like 1	0	19.721	16	7	456	50.2	6.42	31.1
P62750	60S ribosomal protein L23a	0	19.694	42	6	156	17.7	10.45	27.46
P27708	CAD protein	0	19.554	4	7	2225	242.8	6.46	18.16
P17844	probable ATP-dependent RNA helicase DDX5	0	19.491	12	4	614	69.1	8.92	25.3
O00571	ATP-dependent RNA helicase DDX3X	0	19.43	10	6	662	73.2	7.18	27.03
P63104-1	14-3-3 protein zeta/delta	0	19.366	25	3	245	27.7	4.79	29.64
Q9NX63	MICOS complex subunit MIC19	0	19.207	29	6	227	26.1	8.28	29.94
Q9Y3I0	tRNA-splicing ligase RtcB homolog	0	19.14	15	5	505	55.2	7.23	17.91
P62269	40S ribosomal protein S18	0	19.076	43	8	152	17.7	10.99	41.36
P25398	40S ribosomal protein S12	0	19.053	40	5	132	14.5	7.21	31.26

Q9Y383	Putative RNA-binding protein Luc7-like 2	0	18.998	15	5	392	46.5	10.01	25.14
P83731	60S ribosomal protein L24	0	18.948	43	8	157	17.8	11.25	25.7
P62753	40S RIBOSOMAL PROTEIN S6	0	18.923	25	6	249	28.7	10.84	30.53
P39019	40S ribosomal protein S19	0	18.881	42	9	145	16.1	10.32	36.06
P62820	Ras-related protein Rab-1A	0	18.546	35	4	205	22.7	6.21	21.5
Q15084-1	Protein disulfide-isomerase A6	0	18.391	18	5	440	48.1	5.08	19.86
Q02790	Peptidyl-prolyl cis-trans isomerase FKBP4	0	18.184	17	6	459	51.8	5.43	21.73
O95831-1	Apoptosis-inducing factor 1, mitochondrial	0	18.131	12	5	613	66.9	8.95	31.28
P07814	Bifunctional glutamate/proline--tRNA ligase	0	18.089	5	6	1512	170.5	7.33	19.69
P46781	40S ribosomal protein S9	0	18.031	43	9	194	22.6	10.65	38.83
Q9Y230	RuvB-like 2	0	18.011	15	6	463	51.1	5.64	21.66
P42167	Lamina-associated polypeptide 2, isoforms beta/gamma	0	17.872	13	4	454	50.6	9.38	16.79

Q13838-1	spliceosome RNA helicase DDX39B	0	17.805	16	7	428	49	5.67	29.63
P08865	40S ribosomal protein SA	0	17.726	23	4	295	32.8	4.87	20.33
P29966	Myristoylated alanine-rich C-kinase substrate	0	17.701	30	5	332	31.5	4.45	19.91
P27348	14-3-3 protein theta	0	17.606	33	4	245	27.7	4.78	31.16
Q14103	heterogeneous nuclear ribonucleoprotein D0	0	17.531	19	5	355	38.4	7.81	20.07
P10412	Histone H1.4	0	17.068	19	1	219	21.9	11.03	28.5
P63173	60s ribosomal protein l38	0	17.012	36	2	70	8.2	10.1	19.33
P05388	60S acidic ribosomal protein P0	0	16.969	20	5	317	34.3	5.97	28.39
P62917	60S ribosomal protein L8	0	16.918	25	5	257	28	11.03	25.17
P22392	nucleoside diphosphate kinase b	0	16.912	30	4	152	17.3	8.41	28.71
P09211	Glutathione S-transferase P	0	16.691	25	4	210	23.3	5.64	17.37
P16403	Histone H1.2	0	16.644	20	1	213	21.4	10.93	26.4
P39656	Dolichyl-diphosphooligosaccharide--protein glycosyltransferase 48 kDa subunit	0	16.626	14	4	456	50.8	6.55	18.09

P21796	voltage-dependent anion-selective channel protein 1	0	16.594	20	4	283	30.8	8.54	22.04
Q92499	ATP-dependent RNA helicase DDX1	0	16.54	9	6	740	82.4	7.23	15.24
P01857	Immunoglobulin heavy constant gamma 1	0	16.497	15	4	330	36.1	8.19	18.94
P50914	60S ribosomal protein L14	0	16.323	18	4	215	23.4	10.93	21.89
Q99729-2	Isoform 2 of Heterogeneous nuclear ribonucleoprotein A/B	0	16.152	15	4	332	35.9	6.95	17.76
P07737	profilin-1	0	16.07	41	5	140	15	8.27	22.94
P41252	isoleucine--tRNA ligase, cytoplasmic	0	15.982	5	5	1262	144.4	6.15	17.81
Q04637	eukaryotic translation initiation factor 4 gamma 1	0	15.822	4	5	1599	175.4	5.33	18.23
Q08211	Atp-dependent rna helicase a	0	15.683	5	5	1270	140.9	6.84	17.67
P62249	40S ribosomal protein S16	0	15.581	38	6	146	16.4	10.21	33.64
P30041	Peroxiredoxin-6	0	15.411	27	5	224	25	6.38	18.58
P60891	ribose-phosphate pyrophosphokinase	0	15.13	12	3	318	34.8	6.98	20.64

	1								
P24752	Acetyl-CoA acetyltransferase, mitochondrial	0	14.85	12	4	427	45.2	8.85	15.87
P25205	DNA replication licensing factor mcm3	0	14.842	10	6	808	90.9	5.77	15.78
P18124	60S ribosomal protein L7	0	14.586	26	6	248	29.2	10.65	24.45
P08195	4F2 cell-surface antigen heavy chain	0	14.58	10	4	630	68	5.01	14.27
Q86V81	THO complex subunit 4	0	14.51	15	3	257	26.9	11.15	17.43
Q07020	60S ribosomal protein L18	0	14.404	27	5	188	21.6	11.72	23.1
Q07065	Cytoskeleton-associated protein 4	0	14.397	8	3	602	66	5.92	16.15
Q9UJZ1	Stomatin-like protein 2, mitochondrial	0	14.371	12	3	356	38.5	7.39	19.01
P53985	Monocarboxylate transporter 1	0	14.214	8	3	500	53.9	8.66	16.4
P05787	Keratin, type II cytoskeletal 8	0	14.188	10	1	483	53.7	5.59	24.31
P61353	60S ribosomal protein L27	0	14.154	42	5	136	15.8	10.56	17.24
P62913	60S ribosomal protein L11	0	14.076	30	5	178	20.2	9.6	17.96
P36542-1	ATP synthase subunit gamma,	0	14.062	18	5	298	33	9.22	12.44

	mitochondrial								
P62847-1	40S ribosomal protein S24	0	14.004	20	2	133	15.4	10.78	11.08
P40939	Trifunctional enzyme subunit alpha, mitochondrial	0	13.989	6	4	763	82.9	9.04	9.21
P62263	40S ribosomal protein S14	0	13.959	38	4	151	16.3	10.05	19.52
P46783	40S ribosomal protein S10	0	13.929	33	5	165	18.9	10.15	26.81
Q14566	DNA replication licensing factor MCM6	0	13.806	7	4	821	92.8	5.41	8.5
P54136-1	arginine--tRNA ligase, cytoplasmic	0	13.745	10	5	660	75.3	6.68	14.5
Q12905	Interleukin enhancer-binding factor 2	0	13.734	10	3	390	43	5.26	10.69
Q9UHB6	LIM domain and actin-binding protein 1	0	13.323	9	5	759	85.2	6.84	7.44
P31946	14-3-3 protein beta/alpha	0	13.291	16	1	246	28.1	4.83	20.58
P02768-1	Serum albumin	0	13.068	7	4	609	69.3	6.28	29.04
P61254	60S ribosomal protein L26	0	12.86	34	2	145	17.2	10.55	22.8
Q86YZ3	Hornerin	0	12.746	6	3	2850	282.2	10.04	14.74
P58107	epiplakin	0	12.743	4	2	5090	555.3	5.6	12.44

P61586	Transforming protein RhoA	0	12.729	24	4	193	21.8	6.1	3.94
P26599	Polypyrimidine tract-binding protein 1	0	12.707	11	5	531	57.2	9.17	15.29
P61513	60S ribosomal protein L37a	0	12.586	42	3	92	10.3	10.43	12.22
P54577	Tyrosine--tRNA ligase, cytoplasmic	0	12.495	10	5	528	59.1	7.05	12.82
P46782	40S ribosomal protein S5	0	12.438	24	6	204	22.9	9.72	18.12
P62979	Ubiquitin-40S ribosomal protein S27a	0	12.362	24	1	156	18	9.64	17.68
P34897-1	Serine hydroxymethyltransferase, mitochondrial	0	12.28	6	1	504	56	8.53	18.77
P69905	Hemoglobin subunit alpha	0	12.238	11	1	142	15.2	8.68	7.33
Q02543	60S ribosomal protein L18a	0	12.217	32	6	176	20.7	10.71	18.62
P07237	Protein disulfide-isomerase	0	12.209	9	4	508	57.1	4.87	12.73
P46776	60S ribosomal protein L27a	0	12.207	28	4	148	16.6	11	17.74
P08708	40S ribosomal protein S17	0	12.19	33	4	135	15.5	9.85	20.17
P52907	F-actin-capping protein subunit alpha-1	0	12.178	17	3	286	32.9	5.69	15.08

O15372	Eukaryotic translation initiation factor 3 subunit H	0	12.151	12	4	352	39.9	6.54	7.13
P46779	60S ribosomal protein L28	0	12.145	23	4	137	15.7	12.02	20.83
P61313-1	60S ribosomal protein L15	0	12.079	22	5	204	24.1	11.62	22.19
P09972	Fructose-bisphosphate aldolase C	0	12.02	11	2	364	39.4	6.87	13.39
P26196	Probable ATP-dependent RNA helicase DDX6	0	11.913	9	2	483	54.4	8.66	17.2
Q9H0U4	ras-related protein Rab-1B	0	11.819	19	1	201	22.2	5.73	14.54
P62258-1	14-3-3 protein epsilon	0	11.778	16	2	255	29.2	4.74	19.61
P28799	Granulins	0	11.671	9	5	593	63.5	6.83	18.96
P55060-1	Exportin-2	0	11.56	4	4	971	110.3	5.77	17.68
Q96HS1-1	Serine/threonine-protein phosphatase Pgam5, mitochondrial	0	11.316	18	6	289	32	8.68	21.24
P45880	Voltage-dependent anion-selective channel protein 2	0	11.284	18	4	294	31.5	7.56	12.75
P60174-1	Isoform 2 of Triosephosphate isomerase	0	11.263	18	3	249	26.7	6.9	11.68

P53396-1	ATP-citrate synthase	0	11.244	5	5	1101	120.8	7.33	7.97
Q15717	ELAV-like protein 1	0	11.143	14	4	326	36.1	9.17	15.37
O95232-1	Luc7-like protein 3	0	11.098	9	3	432	51.4	9.79	4.72
Q04837	Single-stranded DNA-binding protein, mitochondrial	0	10.888	28	3	148	17.2	9.6	12.44
P62805	histone H4	0	10.831	41	4	103	11.4	11.36	16.97
P24539	ATP synthase F(0) complex subunit B1, mitochondrial	0	10.81	17	4	256	28.9	9.36	10.61
Q00341-1	Vigilin	0	10.771	3	3	1268	141.4	6.87	4.8
P30153	serine/threonine-protein phosphatase 2A 65 kDa regulatory subunit A alpha isoform	0	10.688	6	3	589	65.3	5.11	9.51
P39748	Flap endonuclease 1	0	10.616	11	4	380	42.6	8.62	11.24
P37802	Transgelin-2	0	10.538	23	5	199	22.4	8.25	13.41
Q9UNX3	60S ribosomal protein L26-like 1	0	10.449	28	1	145	17.2	10.55	12.09
Q13283	Ras GTPase-activating protein-binding protein 1	0	10.411	7	2	466	52.1	5.52	10.95

P29692	Elongation factor 1-delta	0	10.398	14	3	281	31.1	5.01	11.26
Q04726	Transducin-like enhancer protein 3	0	10.367	6	4	772	83.4	7.2	14.29
P01023	alpha-2-macroglobulin	0	10.23	3	3	1474	163.2	6.46	14.99
P24534	Elongation factor 1-beta	0	10.214	16	3	225	24.7	4.67	16.4
Q96CS3	FAS-associated factor 2	0	10.162	10	4	445	52.6	5.62	10.49
P13073	Cytochrome c oxidase subunit 4 isoform 1, mitochondrial	0	10.108	26	4	169	19.6	9.51	16.37
P61981	14-3-3 protein gamma	0	10.089	16	1	247	28.3	4.89	17.23
P20700	Lamin-B1	0	10.059	9	4	586	66.4	5.16	14.53
P62851	40S ribosomal protein S25	0	10.041	30	4	125	13.7	10.11	17.73
Q8N8S7	Protein enabled homolog	0	9.945	7	3	591	66.5	6.93	10.74
P10515	Dihydrolipoyllysine-residue acetyltransferase component of pyruvate dehydrogenase complex, mitochondrial	0	9.905	6	3	647	69	7.84	11.53
P48047	ATP synthase subunit O, mitochondrial	0	9.763	20	3	213	23.3	9.96	11.3

P61204	ADP-ribosylation factor 3	0	9.652	22	3	181	20.6	7.43	15.54
Q5QNW6-1	Histone H2B type 2-F	0	9.634	40	6	126	13.9	10.32	13.44
P61006	Ras-related protein Rab-8A	0	9.565	19	3	207	23.7	9.07	8.26
P35637-1	RNA-binding protein FUS	0	9.541	5	3	526	53.4	9.36	11.6
Q14974	Importin subunit beta-1	0	9.32	5	3	876	97.1	4.78	12
P16989-1	Y-box-binding protein 3	0	9.279	10	1	372	40.1	9.77	9.94
P47756-1	F-actin-capping protein subunit beta	0	9.271	17	4	277	31.3	5.59	9.81
P62266	40S ribosomal protein S23	0	9.264	29	4	143	15.8	10.49	22.76
Q71UM5	40S ribosomal protein S27-like	0	9.26	25	2	84	9.5	9.45	12.39
P62081	40S ribosomal protein S7	0	9.238	19	4	194	22.1	10.1	16.52
P80748	Immunoglobulin lambda variable 3-21	0	9.182	23	2	117	12.4	5.29	13.3
P62987	Ubiquitin-60S ribosomal protein L40	0	9.055	27	1	128	14.7	9.83	14.49
P56192	Methionine--tRNA ligase, cytoplasmic	0	8.965	4	3	900	101.1	6.16	10.69
P84098	60S ribosomal protein L19	0	8.753	20	5	196	23.5	11.47	14.11
Q14697-1	Neutral alpha-glucosidase AB	0	8.749	5	3	944	106.8	6.14	14.12

P55209	Nucleosome assembly protein 1-like 1	0	8.66	7	2	391	45.3	4.46	8.9
O43809	Cleavage and polyadenylation specificity factor subunit 5	0	8.637	11	2	227	26.2	8.82	9.87
P62829	60S ribosomal protein L23	0	8.565	21	2	140	14.9	10.51	10.34
P51572	B-cell receptor-associated protein 31	0	8.554	16	5	246	28	8.44	11.89
P16615	Sarcoplasmic/endoplasmic reticulum calcium ATPase 2	0	8.539	3	2	1042	114.7	5.34	8.86
B5ME19	eukaryotic translation initiation factor 3 subunit C-like protein	0	8.463	4	3	914	105.4	5.64	12.41
P62491-1	Ras-related protein Rab-11A	0	8.462	19	4	216	24.4	6.57	11.37
Q9UHI6	Probable ATP-dependent RNA helicase DDX20	0	8.307	5	3	824	92.2	6.95	4.76
P51148	Ras-related protein Rab-5C	0	8.272	12	2	216	23.5	8.41	7.63
P05455	Lupus La protein	0	8.272	6	2	408	46.8	7.12	9.9
P51149	ras-related protein Rab-7a	0	8.271	12	2	207	23.5	6.7	11.07
Q9Y490	Talin-1	0	8.227	2	4	2541	269.6	6.07	2.79

O75396	Vesicle-trafficking protein SEC22b	0	8.157	11	2	215	24.6	6.92	8.9
P49207	60S ribosomal protein L34	0	8.151	29	5	117	13.3	11.47	14.89
Q9P2J5	Leucine--tRNA ligase, cytoplasmic	0	8.048	3	2	1176	134.4	7.3	7.95
Q9NS69	Mitochondrial import receptor subunit TOM22 homolog	0	8.019	26	2	142	15.5	4.34	6.67
P49755	Transmembrane emp24 domain-containing protein 10	0	7.997	11	2	219	25	7.44	8.83
Q9ULV4	coronin-1C	0	7.864	11	4	474	53.2	7.08	13.42
Q9UHX1-1	poly(U)-binding-splicing factor PUF60	0	7.756	7	2	559	59.8	5.29	6.14
Q9P2E9-1	Ribosome-binding protein 1	0	7.744	2	3	1410	152.4	8.6	9.37
P21333	Filamin-A	0	7.679	2	4	2647	280.6	6.06	6.38
Q16576-1	Histone-binding protein RBBP7	0	7.676	4	1	425	47.8	5.05	7.89
P63167	Dynein light chain 1, cytoplasmic	0	7.644	20	2	89	10.4	7.4	4.93
P49588	Alanine--tRNA ligase, cytoplasmic	0	7.637	4	4	968	106.7	5.53	7.82
Q9NRN7	L-aminoadipate-semialdehyde dehydrogenase-phosphopantetheinyl	0	7.617	5	1	309	35.8	6.8	9.64

	transferase								
P20742	Pregnancy zone protein	0	7.562	2	1	1482	163.8	6.38	7.14
Q01081	Splicing factor U2AF 35 kDa subunit	0	7.516	19	3	240	27.9	8.81	5.05
P01619	Immunoglobulin kappa variable 3-20	0	7.486	14	1	116	12.5	4.96	8.07
P32119	Peroxiredoxin-2	0	7.395	13	2	198	21.9	5.97	9.07
P50454	Serpin H1	0	7.185	8	2	418	46.4	8.69	5.99
P51114-1	Fragile X mental retardation syndrome-related protein 1	0	7.143	6	3	621	69.7	6.15	7.35
Q15181	Inorganic pyrophosphatase	0	7.128	11	2	289	32.6	5.86	3.08
Q96AG4	Leucine-rich repeat-containing protein 59	0	7.083	7	1	307	34.9	9.57	3.2
Q6UB35-1	Monofunctional C1-tetrahydrofolate synthase, mitochondrial	0	7.013	4	3	978	105.7	8.06	6.33
Q9NYF8-1	Bcl-2-associated transcription factor 1	0	6.928	2	2	920	106.1	9.98	8.35
P20042	Eukaryotic translation initiation	0	6.918	9	2	333	38.4	5.8	2.74

	factor 2 subunit 2								
Q6IS14	Eukaryotic translation initiation factor 5A-1-like	0	6.896	25	3	154	16.8	5	6.28
O15371	Eukaryotic translation initiation factor 3 subunit D	0	6.889	3	1	548	63.9	6.05	4.9
O00264	Membrane-associated progesterone receptor component 1	0	6.876	16	3	195	21.7	4.7	9.37
P35613	Basigin	0	6.864	8	2	385	42.2	5.66	8.49
O60885-1	Bromodomain-containing protein 4	0	6.801	3	3	1362	152.1	9.19	4.56
P62899	60S ribosomal protein L31	0	6.768	18	2	125	14.5	10.54	11.13
Q9Y3U8	60S ribosomal protein L36	0	6.757	24	3	105	12.2	11.59	9.03
P18077	60S ribosomal protein L35a	0	6.716	32	4	110	12.5	11.06	11.53
Q03252	Lamin-B2	0	6.713	7	3	620	69.9	5.59	7.48
P11177	Pyruvate dehydrogenase E1 component subunit beta, mitochondrial	0	6.699	8	2	359	39.2	6.65	5.14
P00387	NADH-cytochrome b5 reductase 3	0	6.67	8	2	301	34.2	7.59	7.99

Q8N5F7	NF-kappa-B-activating protein	0	6.67	6	2	415	47.1	10.11	7.08
P46777	60S ribosomal protein L5	0	6.647	8	2	297	34.3	9.72	3.87
O95714	E3 ubiquitin-protein ligase HERC2	0	6.645	1	3	4834	526.9	6.28	2.43
P61026	ras-related protein rab-10	0	6.613	10	2	200	22.5	8.38	5.02
Q96AE4	Far upstream element-binding protein 1	0	6.589	6	1	644	67.5	7.61	8.79
P33993-1	DNA replication licensing factor MCM7	0	6.531	4	3	719	81.3	6.46	9.12
P84090	Enhancer of rudimentary homolog	0	6.445	22	2	104	12.3	5.92	5.15
P40429	60S ribosomal protein L13a	0	6.359	13	3	203	23.6	10.93	9.04
Q9UHD8-1	Septin-9	0	6.319	6	3	586	65.4	8.97	6.14
Q9UJV9	Probable ATP-dependent RNA helicase DDX41	0	6.279	7	3	622	69.8	6.84	2.36
P09429	High mobility group protein B1	0	6.266	7	1	215	24.9	5.74	8.95
P14866	Heterogeneous nuclear ribonucleoprotein L	0	6.259	5	3	589	64.1	8.22	5.5
P12532	Creatine kinase U-type,	0	6.188	10	3	417	47	8.34	7.6

	mitochondrial								
Q92598	Heat shock protein 105 kDa	0	6.18	2	2	858	96.8	5.39	6.92
Q14498-1	RNA-binding protein 39	0	6.179	3	1	530	59.3	10.1	8.51
Q96PK6-1	RNA-binding protein 14	0	6.146	8	2	669	69.4	9.67	3.97
P62841	40S ribosomal protein S15	0	6.127	8	1	145	17	10.39	4.98
P05386	60S acidic ribosomal protein P1	0	6.069	14	1	114	11.5	4.32	5.8
P50750	Cyclin-dependent kinase 9	0	6.041	6	2	372	42.8	8.79	7.5
P61163	Alpha-centractin	0	5.997	9	2	376	42.6	6.64	4.65
Q07666	KH domain-containing, RNA-binding, signal transduction-associated protein 1	0	5.953	5	2	443	48.2	8.66	6.23
P62306	Small nuclear ribonucleoprotein F	0	5.939	24	2	86	9.7	4.67	9.27
P13861	cAMP-dependent protein kinase type II-alpha regulatory subunit	0	5.89	6	3	404	45.5	5.07	5.95
Q9Y266	nuclear migration protein nudC	0	5.824	9	3	331	38.2	5.38	9.03
Q9H773	dCTP pyrophosphatase 1	0	5.768	8	1	170	18.7	5.03	7.01
Q96PK6-5	Isoform 5 of RNA-binding protein	0	5.727	9	1	339	37	8.48	2.46

	14								
P61604	10 kDa heat shock protein, mitochondrial	0	5.723	24	2	102	10.9	8.92	5.89
Q13642-1	Isoform 1 of Four and a half LIM domains protein 1	0	5.721	13	3	280	31.9	8.32	8.27
P49736	DNA replication licensing factor mcm2	0	5.698	3	2	904	101.8	5.52	2.97
P61019-1	Ras-related protein Rab-2A	0	5.633	7	1	212	23.5	6.54	8.03
Q14739	Lamin-B receptor	0	5.626	3	2	615	70.7	9.36	8.05
Q9UQ35	serine/arginine repetitive matrix protein 2	0	5.625	1	2	2752	299.4	12.06	10.49
P52292	Importin subunit alpha-1	0	5.612	3	1	529	57.8	5.4	3.52
P13674-1	prolyl 4-hydroxylase subunit alpha-1	0	5.514	4	2	534	61	6.01	2.95
Q9UMS4	Pre-mRNA-processing factor 19	0	5.483	8	2	504	55.1	6.61	4.35
P23526-1	Adenosylhomocysteinase	0	5.475	6	2	432	47.7	6.34	7.02
P49792	E3 SUMO-protein ligase RanBP2	0	5.444	1	2	3224	358	6.2	2.4
P62195-1	26S proteasome regulatory subunit 8	0	5.398	3	1	406	45.6	7.55	5.02

P47897	glutamine--tRNA ligase	0	5.382	2	1	775	87.7	7.15	2.71
Q96H79	Zinc finger CCCH-type antiviral protein 1-like	0	5.37	7	1	300	32.9	8.13	2.35
O75489	NADH dehydrogenase [ubiquinone] iron-sulfur protein 3, mitochondrial	0	5.361	10	2	264	30.2	7.5	6.91
P30101	Protein disulfide-isomerase A3	0	5.341	4	2	505	56.7	6.35	4.72
Q15287-1	RNA-binding protein with serine-rich domain 1	0	5.322	5	1	305	34.2	11.84	5.06
Q9NR30-1	Nucleolar RNA helicase 2	0	5.279	3	2	783	87.3	9.28	4.51
P62191	26S proteasome regulatory subunit 4	0	5.231	5	2	440	49.2	6.21	2.58
P06744	glucose-6-phosphate isomerase	0	5.218	4	2	558	63.1	8.32	5.58
P49756-1	RNA-binding protein 25	0	5.209	2	1	843	100.1	6.32	5.2
Q13555	Calcium/calmodulin-dependent protein kinase type II subunit gamma	0	5.199	5	2	558	62.6	7.83	0
Q8IZP2	Putative protein FAM10A4	0	5.166	10	2	240	27.4	5.08	6.52
Q8WWM7-1	ataxin-2-like protein	0	5.12	4	2	1075	113.3	8.59	5.13
Q8WUM4	Programmed cell death 6-interacting	0	5.057	1	1	868	96	6.52	2.64

	protein								
O43143	Pre-mRNA-splicing factor ATP-dependent RNA helicase DHX15	0	5.033	3	2	795	90.9	7.46	4.06
P84103	Serine/arginine-rich splicing factor 3	0	4.875	14	2	164	19.3	11.65	2.44
Q9NX58	Cell growth-regulating nucleolar protein	0	4.87	5	2	379	43.6	9.54	5.58
Q9BZZ5-4	Apoptosis inhibitor 5	0	4.857	3	1	524	59	7.34	3.48
O95793	double-stranded RNA-binding protein Staufen homolog 1	0	4.838	2	1	577	63.1	9.44	4.38
Q13247	Serine/arginine-rich splicing factor 6	0	4.771	5	2	344	39.6	11.43	6.25
P01859	Immunoglobulin heavy constant gamma 2	0	4.768	8	1	326	35.9	7.59	4.19
Q15428	splicing factor 3a subunit 2	0	4.672	3	1	464	49.2	9.64	3.54
Q53H12	Acylglycerol kinase, mitochondrial	0	4.652	9	2	422	47.1	8.09	4.8
P62316	Small nuclear ribonucleoprotein Sm D2	0	4.644	16	2	118	13.5	9.91	10.49
P46778	60S ribosomal protein L21	0	4.64	7	1	160	18.6	10.49	6.65

Q9P035	Very-long-chain (3R)-3-hydroxyacyl-CoA dehydratase 3	0	4.621	4	1	362	43.1	8.94	5.71
P04844-1	Dolichyl-diphosphooligosaccharide--protein glycosyltransferase subunit 2	0	4.62	2	1	631	69.2	5.69	7.17
Q13155	aminoacyl tRNA synthase complex-interacting multifunctional protein 2	0	4.618	8	1	320	35.3	8.22	3.79
P22061	protein-L-isoaspartate(D-aspartate) O-methyltransferase	0	4.588	12	2	227	24.6	7.21	3.97
P49915	GMP synthase [glutamine-hydrolyzing]	0	4.58	3	2	693	76.7	6.87	6.34
Q9UPN4	centrosomal protein of 131 kDa	0	4.527	1	1	1083	122.1	8.69	1.79
Q9BXP5	serrate RNA effector molecule homolog	0	4.523	2	1	876	100.6	5.96	3.79
Q7RTS7	Keratin, type II cytoskeletal 74	0	4.491	5	1	529	57.8	7.71	13.16
P35268	60S ribosomal protein L22	0	4.483	10	1	128	14.8	9.19	7.92
Q9UMR2-1	ATP-dependent RNA helicase DDX19B	0	4.479	3	1	479	53.9	6.3	6.46

P00918	Carbonic anhydrase 2	0	4.444	7	2	260	29.2	7.4	4.51
Q9HCD5	Nuclear receptor coactivator 5	0	4.437	4	2	579	65.5	9.6	1.82
P62861	40S ribosomal protein S30	0	4.362	31	2	59	6.6	12.15	5.04
Q9UKG1	DCC-interacting protein 13-alpha	0	4.361	3	2	709	79.6	5.41	2.49
Q96C36	Pyrroline-5-carboxylate reductase 2	0	4.361	7	2	320	33.6	7.77	3.52
O43242	26S proteasome non-ATPase regulatory subunit 3	0	4.36	2	1	534	60.9	8.44	5.85
Q96A26	Protein FAM162A	0	4.331	7	1	154	17.3	9.77	3.84
P42766	60S ribosomal protein L35	0	4.291	15	2	123	14.5	11.05	8.85
Q14008	Cytoskeleton-associated protein 5	0	4.289	2	2	2032	225.4	7.8	5.73
Q96TC7	Regulator of microtubule dynamics protein 3	0	4.289	4	1	470	52.1	5.1	2.54
P62873	Guanine nucleotide-binding protein G(I)/G(S)/G(T) subunit beta-1	0	4.258	6	2	340	37.4	6	2.17
P62244	40S ribosomal protein S15a	0	4.211	18	3	130	14.8	10.13	11.75
P02765	Alpha-2-HS-glycoprotein	0	4.199	5	2	367	39.3	5.72	8.49
Q9NRW1	Ras-related protein Rab-6B	0	4.173	6	1	208	23.4	5.53	4.92

O75717	WD repeat and HMG-box DNA-binding protein 1	0	4.162	1	1	1129	125.9	5.62	4.94
Q9NZ45	CDGSH iron-sulfur domain-containing protein 1	0	4.134	14	1	108	12.2	9.09	2.18
Q13435	Splicing factor 3b subunit 2	0	4.131	3	2	895	100.2	5.67	2.88
P26640	Valine--tRNA ligase	0	4.112	1	1	1264	140.4	7.59	1.86
P52732	Kinesin-like protein KIF11	0	4.102	2	2	1056	119.1	5.64	3.8
O43324-1	Eukaryotic translation elongation factor 1 epsilon-1	0	4.075	6	1	174	19.8	8.54	4.6
P13489	Ribonuclease inhibitor	0	4.071	3	1	461	49.9	4.82	2.32
Q9H9B4	Sideroflexin-1	0	4.067	4	1	322	35.6	9.07	7.31
Q00325-1	Phosphate carrier protein, mitochondrial	0	4.058	5	2	362	40.1	9.38	5.96
Q15029	116 kDa U5 small nuclear ribonucleoprotein component	0	4.014	2	1	972	109.4	5	7.86
O14929	histone acetyltransferase type B catalytic subunit	0	3.983	3	1	419	49.5	5.69	5.37

P19474	E3 ubiquitin-protein ligase TRIM21	0	3.976	4	2	475	54.1	6.38	6.19
Q9Y5M8	signal recognition particle receptor subunit beta	0	3.974	12	2	271	29.7	9.04	3.06
P29401	Transketolase	0	3.963	4	1	623	67.8	7.66	2.53
Q86VP6-1	cullin-associated nedd8-dissociated protein 1	0	3.943	1	2	1230	136.3	5.78	2.25
Q15836	Vesicle-associated membrane protein 3	0	3.941	17	1	100	11.3	8.79	2.1
Q16181	Septin-7	0	3.933	5	2	437	50.6	8.63	5.97
P53621-1	coatomer subunit alpha	0	3.929	2	2	1224	138.3	7.66	6.33
Q8WXF1	Paraspeckle component 1	0	3.907	3	1	523	58.7	6.67	1.98
P04637	Cellular tumor antigen p53	0	3.906	5	2	393	43.6	6.79	5.96
Q6NZI2-1	caveolae-associated protein 1	0	3.903	3	1	390	43.5	5.6	6.13
P35606	Coatomer subunit beta'	0	3.893	1	1	906	102.4	5.27	3.9
P23588	eukaryotic translation initiation factor 4B	0	3.857	3	1	611	69.1	5.73	4.45
P05534	HLA class I histocompatibility	0	3.854	7	1	365	40.7	6.34	6

	antigen, A-24 alpha chain								
P22307-1	Non-specific lipid-transfer protein	0	3.837	2	1	547	59	6.89	2.62
P41250	Glycine--tRNA ligase	0	3.813	4	3	739	83.1	7.03	2.55
P67870	Casein kinase II subunit beta	0	3.781	5	1	215	24.9	5.55	4.46
O15397-1	Importin-8	0	3.779	1	1	1037	119.9	5.16	2.77
Q14152	Eukaryotic translation initiation factor 3 subunit A	0	3.752	1	2	1382	166.5	6.79	4.1
Q15459	splicing factor 3A subunit 1	0	3.735	2	1	793	88.8	5.22	4.63
Q9BY77	Polymerase delta-interacting protein 3	0	3.713	3	1	421	46.1	9.99	4.7
P08574	Cytochrome c1, heme protein, mitochondrial	0	3.693	4	1	325	35.4	9	2.82
P51571	translocon-associated protein subunit delta	0	3.674	6	1	173	19	6.15	2.51
Q5BKY9-1	Protein FAM133B	0	3.657	7	2	247	28.4	10.02	1.97
P33991	DNA replication licensing factor MCM4	0	3.61	2	1	863	96.5	6.74	2.4

P49458	Signal recognition particle 9 kDa protein	0	3.607	13	1	86	10.1	7.97	1.93
Q9UKV3-1	Apoptotic chromatin condensation inducer in the nucleus	0	3.575	1	1	1341	151.8	6.43	2
O75821	Eukaryotic translation initiation factor 3 subunit G	0	3.554	4	1	320	35.6	6.13	2.71
P31689-1	DnaJ homolog subfamily A member 1	0	3.525	3	1	397	44.8	7.08	3.02
Q06787	synaptic functional regulator FMR1	0	3.522	3	1	632	71.1	7.42	7.8
P08579	U2 small nuclear ribonucleoprotein B''	0	3.514	5	1	225	25.5	9.72	2.74
P62495	Eukaryotic peptide chain release factor subunit 1	0	3.512	3	1	437	49	5.71	2.12
P62333	26S proteasome regulatory subunit 10B	0	3.493	5	2	389	44.1	7.49	2.4
Q9NR31	GTP-binding protein SAR1a	0	3.46	6	1	198	22.4	6.68	2.18
Q16777	Histone H2A type 2-C	0	3.431	12	2	129	14	10.9	11.43

Q12904-1	aminoacyl tRNA synthase complex-interacting multifunctional protein 1	0	3.399	4	1	312	34.3	8.43	3.56
P62304	small nuclear ribonucleoprotein E	0	3.388	12	1	92	10.8	9.44	2.31
O43776	Asparagine--tRNA ligase, cytoplasmic	0	3.338	3	1	548	62.9	6.25	2.88
B0I1T2-1	unconventional myosin-Ig	0	3.293	2	1	1018	116.4	8.73	3.28
Q8IX12	Cell division cycle and apoptosis regulator protein 1	0	3.288	2	2	1150	132.7	5.76	1.9
P55072	Transitional endoplasmic reticulum ATPase	0	3.28	2	1	806	89.3	5.26	5.36
P62891	60S ribosomal protein L39	0	3.218	20	1	51	6.4	12.56	7.94
Q9NRP0-1	oligosaccharyltransferase complex subunit OSTC	0	3.188	8	1	149	16.8	9.13	4.39
Q7Z6E9	E3 ubiquitin-protein ligase RBBP6	0	3.188	1	2	1792	201.4	9.64	2.13
Q16630	Cleavage and polyadenylation specificity factor subunit 6	0	3.183	5	2	551	59.2	7.15	3.5
Q8N684-1	Cleavage and polyadenylation	0	3.178	2	1	471	52	8	5.08

	specificity factor subunit 7								
Q9BWJ5	Splicing factor 3B subunit 5	0	3.17	13	1	86	10.1	6.35	2.31
P27144	Adenylate kinase 4, mitochondrial	0	3.158	6	1	223	25.3	8.4	2.81
P55884	Eukaryotic translation initiation factor 3 subunit B	0	3.149	1	1	814	92.4	5	2.21
P67936	Tropomyosin alpha-4 chain	0	3.147	4	1	248	28.5	4.69	2.01
P09661	U2 small nuclear ribonucleoprotein A'	0	3.128	4	1	255	28.4	8.62	4.89
Q66PJ3-1	ADP-ribosylation factor-like protein 6-interacting protein 4	0	3.121	4	1	421	44.9	10.93	2.54
P23193	Transcription elongation factor A protein 1	0	3.055	4	1	301	33.9	8.38	2.92
Q09666-1	Neuroblast differentiation-associated protein AHNAK	0	3.052	1	1	5890	628.7	6.15	1.61
P18085	ADP-ribosylation factor 4	0	3.025	6	1	180	20.5	7.14	2.46
Q9HB71	Calcyclin-binding protein	0	3.012	4	1	228	26.2	8.25	0
O00139	Kinesin-like protein KIF2A	0	2.989	2	1	706	79.9	6.68	1.98

O43707	Alpha-actinin-4	0	2.975	1	1	911	104.8	5.44	2.04
Q9Y277	Voltage-dependent anion-selective channel protein 3	0	2.974	4	1	283	30.6	8.66	6.39
P62854	40S ribosomal protein S26	0	2.965	21	2	115	13	11	3.49
P27694	Replication protein A 70 kDa DNA-binding subunit	0	2.949	5	2	616	68.1	7.21	1.71
Q16186	Proteasomal ubiquitin receptor ADRM1	0	2.942	4	1	407	42.1	5.07	4.58
Q5SW79-1	Centrosomal protein of 170 kDa	0	2.927	1	1	1584	175.2	7.11	1.8
Q9NSE4	Isoleucine--tRNA ligase, mitochondrial	0	2.843	1	1	1012	113.7	7.2	2.23
P56545	c-terminal-binding protein 2	0	2.831	4	1	445	48.9	6.95	0
O14980	Exportin-1	0	2.826	2	2	1071	123.3	6.06	2.33
O60841	Eukaryotic translation initiation factor 5B	0	2.824	1	1	1220	138.7	5.49	2.26
P14174	Macrophage Migration inhibitory factor	0	2.771	8	1	115	12.5	7.88	8.06

Q6PKG0-1	La-related protein 1	0	2.744	1	1	1096	123.4	8.82	5.7
Q86UP2-1	Kinectin	0	2.727	1	1	1357	156.2	5.64	2.04
Q15019	septin-2	0	2.719	4	1	361	41.5	6.6	1.77
A0A075B6S5	immunoglobulin kappa variable 1-27	0	2.707	14	1	117	12.7	8.29	5.28
O95613	Pericentrin	0	2.705	1	2	3336	377.8	5.55	3.84
Q5D862	Filaggrin-2	0	2.685	1	1	2391	247.9	8.31	2.35
Q12874	splicing factor 3a subunit 3	0	2.681	3	2	501	58.8	5.38	3.67
P60953	Cell division control protein 42 homolog	0	2.673	5	1	191	21.2	6.55	0
P60763	Ras-related C3 botulinum toxin substrate 3	0	2.666	5	1	192	21.4	8.15	1.63
P04181-1	Ornithine aminotransferase, mitochondrial	0	2.663	3	1	439	48.5	7.03	1.87
Q92734-1	Protein TFG	0	2.652	3	1	400	43.4	5.1	4.35
P25789	Proteasome subunit alpha type-4	0	2.651	4	1	261	29.5	7.72	1.62
Q9Y262	eukaryotic translation initiation factor 3 subunit L	0	2.624	2	1	564	66.7	6.34	4.99

P61221	ATP-binding cassette sub-family E member 1	0	2.601	2	1	599	67.3	8.34	2.36
Q92522	Histone H1x	0	2.572	7	1	213	22.5	10.76	1.91
P04792	Heat shock protein beta-1	0	2.566	5	1	205	22.8	6.4	1.95
P02786	Transferrin receptor protein 1	0	2.554	1	1	760	84.8	6.61	2.74
P26368	Splicing factor U2AF 65 kDa subunit	0	2.542	4	1	475	53.5	9.09	2.11
Q15154-1	Pericentriolar material 1 protein	0	2.538	1	2	2024	228.4	5.02	0
Q9HCE1	Putative helicase MOV-10	0	2.508	1	1	1003	113.6	8.82	1.61
P56385	ATP synthase subunit e, mitochondrial	0	2.508	14	1	69	7.9	9.35	4.5
Q99961	Endophilin-A2	0	2.506	3	1	368	41.5	5.43	4.64
Q969Q0	60S ribosomal protein L36a-like	0	2.482	8	1	106	12.5	10.65	4.59
Q9H078-1	Caseinolytic peptidase B protein homolog	0	2.446	3	1	707	78.7	9.01	0
P61421	V-type proton ATPase subunit d 1	0	2.445	3	1	351	40.3	5	1.75
P35998	26S proteasome regulatory subunit 7	0	2.428	3	1	433	48.6	5.95	0

Q8WXD5	gem-associated protein 6	0	2.41	8	1	167	18.8	5.12	1.85
P40926	Malate dehydrogenase, mitochondrial	0	2.406	4	2	338	35.5	8.68	2.32
O75400	pre-mRNA-processing factor 40 homolog A	0	2.401	1	1	957	108.7	7.56	0
O75964	ATP synthase subunit g, mitochondrial	0	2.384	11	1	103	11.4	9.64	2.16
P33240	cleavage stimulation factor subunit 2	0	2.364	2	1	577	60.9	6.83	2.53
Q96P70	Importin-9	0	2.346	1	1	1041	115.9	4.81	1.97
P11310-1	medium-chain specific acyl-CoA dehydrogenase, mitochondrial	0	2.343	3	1	421	46.6	8.37	0
Q8N9Q2	protein SREK1IP1	0	2.323	6	1	155	18.2	9.85	2.18
P13804-1	Electron transfer flavoprotein subunit alpha, mitochondrial	0	2.311	6	2	333	35.1	8.38	0
P30086	phosphatidylethanolamine-binding protein 1	0	2.307	4	1	187	21	7.53	0
Q9Y2L1-1	exosome complex exonuclease	0	2.306	2	1	958	108.9	7.14	1.68

	RRP44								
Q96T37-1	Putative RNA-binding protein 15	0	2.287	2	1	977	107.1	10.08	1.72
P62318	small nuclear ribonucleoprotein sm d3	0	2.284	7	1	126	13.9	10.32	1.89
O60749	Sorting nexin-2	0	2.281	2	1	519	58.4	5.12	2.19
P00846	ATP synthase subunit A	0	2.276	4	1	226	24.8	10.1	0
Q9Y295	developmentally-regulated GTP-binding protein 1	0	2.256	3	1	367	40.5	8.9	2
P62714	serine/threonine-protein phosphatase 2A catalytic subunit beta isoform	0	2.245	4	1	309	35.6	5.43	2.01
Q9NUP9	Protein lin-7 homolog C	0	2.242	6	1	197	21.8	8.43	1.77
P00403	Cytochrome c oxidase subunit 2	0	2.229	4	1	227	25.5	4.82	4.22
Q13428-1	Treacle protein	0	2.218	1	1	1488	152	9.04	2.79
Q9Y3Y2	Chromatin target of PRMT1 protein	0	2.208	6	1	248	26.4	12.23	0
O95747	Serine/threonine-protein kinase OSR1	0	2.205	3	1	527	58	6.43	4.25
O75369-1	Filamin-B	0	2.201	1	1	2602	278	5.73	2.29

P62910	60S ribosomal protein L32	0	2.197	7	1	135	15.9	11.33	2.82
Q9P289	serine/threonine-protein kinase 26	0	2.195	4	1	416	46.5	5.29	1.92
P42704	Leucine-rich PPR motif-containing protein, mitochondrial	0	2.195	1	1	1394	157.8	6.13	2.01
O00231	26S proteasome non-ATPase regulatory subunit 11	0	2.191	3	1	422	47.4	6.48	2.14
A0A075B6I1	immunoglobulin lambda variable 4-60	0	2.186	8	1	120	13	6.25	7.1
P49591	Serine--tRNA ligase, cytoplasmic	0	2.179	2	1	514	58.7	6.43	2.02
O75534	cold shock domain-containing protein E1	0	2.171	1	1	798	88.8	6.25	2.55
Q9NQC3	Reticulon-4	0	2.143	1	1	1192	129.9	4.5	2.5
P27816-1	Microtubule-associated protein 4	0	2.142	1	1	1152	120.9	5.43	4.83
Q6UN15-1	Pre-mRNA 3'-end-processing factor FIP1	0	2.134	2	1	594	66.5	5.59	0
P40925	Malate dehydrogenase, cytoplasmic	0	2.128	4	1	334	36.4	7.36	2.14
Q7Z739	YTH domain-containing family	0	2.118	2	1	585	63.8	9.04	1.86

	protein 3								
O00273-1	DNA fragmentation factor subunit alpha	0	2.106	2	1	331	36.5	4.79	0
Q9Y2W1	Thyroid hormone receptor-associated protein 3	0	2.086	1	1	955	108.6	10.15	2.29
P48556	26S proteasome non-ATPase regulatory subunit 8	0	2.083	3	1	350	39.6	9.7	1.7
P59190-1	Ras-related protein Rab-15	0	2.076	5	1	212	24.4	5.71	2.71
P47914	60S ribosomal protein L29	0	2.069	5	1	159	17.7	11.66	3.96
Q14677-1	Clathrin interactor 1	0	2.039	2	1	625	68.2	6.42	0
O60232	Sjoegren syndrome/scleroderma autoantigen 1	0	2.034	4	1	199	21.5	5.24	1.93
Q16543	Hsp90 co-chaperone Cdc37	0	2.029	2	1	378	44.4	5.25	1.94
P53999	Activated RNA polymerase II transcriptional coactivator p15	0.001	2.011	9	1	127	14.4	9.6	2.32
P36957	Dihydrolipoyllysine-residue succinyltransferase component of 2-	0.001	1.991	3	1	453	48.7	8.95	3.68

	oxoglutarate dehydrogenase complex, mitochondrial								
P00505	Aspartate aminotransferase, mitochondrial	0.001	1.989	2	1	430	47.5	9.01	2.43
Q92900	Regulator of nonsense transcripts 1	0.003	1.977	2	2	1129	124.3	6.61	0
P09669	Cytochrome c oxidase subunit 6C	0.003	1.949	11	1	75	8.8	10.39	0
P40616	ADP-ribosylation factor-like protein 1	0.003	1.947	5	1	181	20.4	5.72	1.63
Q8N1F7-1	Nuclear pore complex protein Nup93	0.003	1.928	1	1	819	93.4	5.72	2.02
Q9Y6C9	Mitochondrial carrier homolog 2	0.003	1.926	4	1	303	33.3	7.97	2.01
P48444	Coatomer subunit delta	0.003	1.907	2	1	511	57.2	6.21	3.94
P18669	Phosphoglycerate mutase 1	0.003	1.907	4	1	254	28.8	7.18	4.57
Q15637	Splicing factor 1	0.003	1.905	2	1	639	68.3	8.98	2.32
O95292	Vesicle-associated membrane protein-associated protein B/C	0.003	1.898	5	1	243	27.2	7.3	0
O95816	BAG family molecular chaperone regulator 2	0.003	1.89	4	1	211	23.8	6.7	2.24

Q9NWB6	Arginine and glutamate-rich protein 1	0.003	1.876	3	1	273	33.2	10.35	1.71
Q08J23	tRNA (Cytosine(34)-C(5))-methyltransferase	0.003	1.873	1	1	767	86.4	6.77	2.03
O43399	Tumor protein D54	0.003	1.861	5	1	206	22.2	5.36	3.91
Q16637	Survival motor neuron protein	0.003	1.843	2	1	294	31.8	6.55	4.73
Q1KMD3	heterogeneous nuclear ribonucleoprotein U-like protein 2	0.003	1.827	4	2	747	85.1	4.91	0
Q04760-1	lactoylglutathione lyase	0.003	1.819	5	1	184	20.8	5.31	2
Q9BPW8	Protein NipSnap homolog 1	0.003	1.816	4	1	284	33.3	9.31	5.21
Q5VTU8	ATP synthase subunit epsilon-like protein, mitochondrial	0.003	1.809	14	1	51	5.8	10.14	0
P51570	galactokinase	0.003	1.803	3	1	392	42.2	6.46	2.02
P22102-1	trifunctional purine biosynthetic protein adenosine-3	0.003	1.788	2	1	1010	107.7	6.7	2.19
Q15393-1	Splicing factor 3B subunit 3	0.003	1.781	1	1	1217	135.5	5.26	2.05
Q9NSD9	Phenylalanine--tRNA ligase beta	0.003	1.778	2	1	589	66.1	6.84	3.96

	subunit								
O94906-1	Pre-mRNA-processing factor 6	0.003	1.777	1	1	941	106.9	8.25	2.04
P49321	Nuclear autoantigenic sperm protein	0.003	1.752	1	1	788	85.2	4.3	1.99
P61923	Coatomer subunit zeta-1	0.003	1.749	6	1	177	20.2	4.81	1.63
P35520-1	Cystathionine beta-synthase	0.003	1.749	2	1	551	60.5	6.65	2.29
O75643-1	U5 small nuclear ribonucleoprotein 200 kDa helicase	0.003	1.738	0	1	2136	244.4	6.06	1.94
Q15363	Transmembrane emp24 domain-containing protein 2	0.003	1.73	4	1	201	22.7	5.17	0
O15479	Melanoma-associated antigen B2	0.003	1.717	4	1	319	35.3	8.76	0
Q14254	Flotillin-2	0.003	1.712	2	1	428	47	5.25	0
Q16822-1	Phosphoenolpyruvate carboxykinase [GTP], mitochondrial	0.003	1.705	2	1	640	70.7	7.62	1.66
O76094	Signal recognition particle subunit SRP72	0.003	1.7	1	1	671	74.6	9.26	1.73
Q6P2E9-1	Enhancer of mRNA-decapping protein 4	0.003	1.696	1	1	1401	151.6	5.86	3.87

Q96JN8-1	Neuralized-like protein 4	0.003	1.691	0	1	1562	166.8	5.86	0
P60468	protein transport protein Sec61 subunit beta	0.003	1.689	10	1	96	10	11.56	2.1
P07477	Trypsin-1	0.003	1.667	3	1	247	26.5	6.51	3.61
P37108	Signal recognition particle 14 kDa protein	0.003	1.64	6	1	136	14.6	10.04	2.05
O75746	Calcium-binding mitochondrial carrier protein Aralar1	0.003	1.636	1	1	678	74.7	8.38	0
P07741-1	Adenine phosphoribosyltransferase	0.003	1.635	4	1	180	19.6	6.02	0
P28288	ATP-binding cassette sub-family D member 3	0.003	1.631	1	1	659	75.4	9.36	3.32
P13995	Bifunctional methylenetetrahydrofolate dehydrogenase/cyclohydrolase, mitochondrial	0.002	1.621	3	1	350	37.9	8.73	2.73
P00492	Hypoxanthine-guanine phosphoribosyltransferase	0.002	1.619	5	1	218	24.6	6.68	0

Q9UNE7-1	E3 ubiquitin-protein ligase CHIP	0.002	1.617	3	1	303	34.8	5.87	1.71
P61619	Protein transport protein Sec61 subunit alpha isoform 1	0.002	1.615	2	1	476	52.2	8.06	4.37
Q8ND56	protein LSM14 homolog A	0.002	1.603	2	1	463	50.5	9.52	0
P23258	tubulin gamma-1 chain	0.003	1.599	4	1	451	51.1	6.14	0
Q16204	coiled-coil domain-containing protein 6	0.003	1.578	2	1	474	53.3	7.34	3.52
Q9NP64	nucleolar protein of 40 kDa	0.003	1.574	3	1	241	27.6	9.7	0
Q13347	Eukaryotic translation initiation factor 3 subunit I	0.003	1.557	3	1	325	36.5	5.64	0
P35611-1	Alpha-adducin	0.003	1.554	1	1	737	80.9	5.83	0
Q8NE71-1	ATP-binding cassette sub-family F member 1	0.003	1.554	1	1	845	95.9	6.8	0
P54727	UV excision repair protein RAD23 homolog B	0.003	1.533	2	1	409	43.1	4.84	1.63
O43684	Mitotic checkpoint protein BUB3	0.003	1.515	3	1	328	37.1	6.84	0
Q8WXE9-1	Stonin-2	0.003	1.496	1	1	905	101.1	5.39	6.58

P30049	ATP synthase subunit delta, mitochondrial	0.003	1.488	5	1	168	17.5	5.49	1.64
O15027-1	Protein transport protein Sec16A	0.003	1.483	1	1	2179	233.4	5.63	0
Q9Y3F4	Serine-threonine kinase receptor-associated protein	0.003	1.481	3	1	350	38.4	5.12	0
P56134	ATP synthase subunit f, mitochondrial	0.003	1.466	12	1	94	10.9	9.67	0
Q06210-1	glutamine--fructose-6-phosphate aminotransferase [isomerizing] 1	0.003	1.466	2	1	699	78.8	7.11	1.64
O14974	Protein phosphatase 1 regulatory subunit 12A	0.003	1.465	3	1	1030	115.2	5.4	2.34
Q9Y520	Protein Prrc2c	0.003	1.463	0	1	2896	316.7	9.13	2.08
Q9H8M2-1	Isoform 5 of Bromodomain-containing protein 9	0.003	1.456	1	1	501	55.6	5.34	0
P12270	Nucleoprotein TPR	0.003	1.437	0	1	2363	267.1	5.02	3.97
Q9NQX3	Gephyrin	0.003	1.432	1	1	736	79.7	5.43	0
O94808	Glutamine--fructose-6-phosphate	0.003	1.405	1	1	682	76.9	7.37	4.3

	aminotransferase [isomerizing] 2								
Q13123	Protein Red	0.003	1.397	1	1	557	65.6	6.64	0
A6NEC2	puromycin-sensitive aminopeptidase-like protein	0.003	1.395	2	1	478	53.7	5.34	0
Q96F86	Enhancer of mRNA-decapping protein 3	0.003	1.39	3	1	508	56	7.11	2.36
Q9H974-1	Queuine tRNA-ribosyltransferase accessory subunit 2	0.003	1.382	5	1	415	46.7	6.81	0
Q13547	histone deacetylase 1	0.003	1.377	2	1	482	55.1	5.48	1.83
Q15738	sterol-4-alpha-carboxylate 3-dehydrogenase, decarboxylating	0.003	1.377	3	1	373	41.9	8.06	1.8
Q15392	Delta(24)-sterol reductase	0.003	1.369	2	1	516	60.1	8.16	0
Q9H3P2-1	Negative elongation factor A	0.003	1.366	3	1	528	57.2	9.03	1.83
Q92616	eIF-2-alpha kinase activator GCN1	0.003	1.357	0	1	2671	292.6	7.47	0
Q99497	protein/nucleic acid deglycase DJ-1	0.003	1.356	4	1	189	19.9	6.79	0
Q99541	perilipin-2	0.003	1.354	2	1	437	48	6.8	2.02
P10644	cAMP-dependent protein kinase type	0.005	1.334	4	1	381	43	5.35	1.62

	I-alpha regulatory subunit								
P84243	histone H3.3	0.005	1.322	7	1	136	15.3	11.27	2.84
Q7RTV0	PHD finger-like domain-containing protein 5A	0.005	1.322	12	1	110	12.4	8.41	0
Q9HC07	Transmembrane protein 165	0.005	1.321	8	1	324	34.9	7.02	0
P01715	Immunoglobulin lambda variable 3-1	0.006	1.319	7	1	115	12.3	4.94	4.48
P54886	delta-1-pyrroline-5-carboxylate synthase	0.006	1.317	1	1	795	87.2	7.12	0
P62136-1	serine/threonine-protein phosphatase PP1-alpha catalytic subunit	0.006	1.303	6	1	330	37.5	6.33	2.5
Q15691	Microtubule-associated protein RP/EB family member 1	0.006	1.296	7	1	268	30	5.14	0
Q9Y285	Phenylalanine--tRNA ligase alpha subunit	0.006	1.296	3	1	508	57.5	7.8	2.78
P55084	Trifunctional enzyme subunit beta, mitochondrial	0.006	1.29	2	1	474	51.3	9.41	0

P08758	annexin A5	0.006	1.29	3	1	320	35.9	5.05	1.64
Q9Y6G9	Cytoplasmic dynein 1 light intermediate chain 1	0.006	1.289	4	1	523	56.5	6.42	0
O60762	Dolichol-phosphate mannosyltransferase subunit 1	0.006	1.286	4	1	260	29.6	9.57	0
Q9BZI7-1	Regulator of nonsense transcripts 3B	0.006	1.286	2	1	483	57.7	9.48	0
Q9UNZ5	Leydig cell tumor 10 kDa protein homolog	0.006	1.273	9	1	99	10.6	11.55	1.71
Q3ZAQ7	Vacuolar ATPase assembly integral membrane protein vma21	0.006	1.273	12	1	101	11.3	7.24	0
P06730	Eukaryotic translation initiation factor 4E	0.006	1.272	3	1	217	25.1	6.15	3.56
Q00577	Transcriptional activator protein Pur-alpha	0.006	1.266	8	1	322	34.9	6.44	0
Q14157-1	Isoform 2 of Ubiquitin-associated protein 2-like	0.006	1.245	2	1	983	103.9	6.98	0
Q9P258	Protein RCC2	0.006	1.238	2	1	522	56	8.78	0

Q99714-1	3-hydroxyacyl-CoA dehydrogenase type-2	0.006	1.235	9	1	261	26.9	7.78	0
P09936	Ubiquitin carboxyl-terminal hydrolase isozyme L1	0.006	1.231	8	1	223	24.8	5.48	0
Q16555-1	Dihydropyrimidinase-related protein 2	0.006	1.202	3	1	572	62.3	6.38	0
Q96SN8	CDK5 regulatory subunit-associated protein 2	0.006	1.202	1	1	1893	214.9	5.58	0
P14324	Farnesyl pyrophosphate synthase	0.006	1.197	2	1	419	48.2	6.15	0
Q9NZP5	Olfactory receptor 5AC2	0.008	1.191	2	1	309	35.3	8.94	4.05
P08621-1	U1 small nuclear ribonucleoprotein 70 kDa	0.011	1.188	2	1	437	51.5	9.94	0
P01705	Immunoglobulin lambda variable 2-23	0.012	1.17	7	1	113	11.9	5.91	4.07
P60981-1	Destrin	0.012	1.163	4	1	165	18.5	7.85	0
Q92851-1	caspase-10	0.012	1.142	3	1	521	58.9	7.33	0
Q9UNF1	Melanoma-associated antigen D2	0.012	1.134	4	1	606	64.9	9.32	0

P30084	Enoyl-CoA hydratase, mitochondrial	0.012	1.129	3	1	290	31.4	8.07	1.71
P49257	Protein ERGIC-53	0.012	1.122	2	1	510	57.5	6.77	0
P11233	Ras-related protein Ral-A	0.012	1.121	3	1	206	23.6	7.11	1.76
Q9BRX2	Protein pelota homolog	0.013	1.105	2	1	385	43.3	6.34	1.9
Q96JB1-1	Dynein heavy chain 8, axonemal	0.013	1.096	0	1	4490	514.3	6.32	2.27
Q9H6R7	WD repeat and coiled-coil-containing protein	0.013	1.096	1	1	721	79.1	6.7	1.68
P08559	Pyruvate dehydrogenase E1 component subunit alpha, somatic form, mitochondrial	0.013	1.095	2	1	390	43.3	8.06	0
Q8IUD2-1	ELKS/Rab6-interacting/CAST family member 1	0.013	1.093	1	1	1116	128	5.97	0
O75844	caax prenyl protease 1 homolog	0.013	1.078	2	1	475	54.8	7.49	0
Q01105	Protein SET	0.015	1.056	4	1	290	33.5	4.32	0
P60228	Eukaryotic translation initiation factor 3 subunit E	0.015	1.055	4	1	445	52.2	6.04	0
Q9P0L0	vesicle-associated membrane	0.015	1.044	2	1	249	27.9	8.62	1.61

	protein-associated protein A								
Q99996	A-kinase anchor protein 9	0.015	1.042	0	1	3911	453.4	4.98	0
Q92973-1	transportin-1	0.015	1.04	2	1	898	102.3	4.98	0
Q07812	Apoptosis regulator BAX	0.015	1.039	7	1	192	21.2	5.22	1.91
Q8IZJ3	C3 and PZP-like alpha-2-macroglobulin domain-containing protein 8	0.026	1.006	0	1	1885	206.6	6.42	0
Q5C9Z4	nucleolar MIF4G domain-containing protein 1	0.026	0.999	2	1	860	96.2	8.1	0
P50402	Emerin	0.027	0.988	3	1	254	29	5.5	1.66
Q5VUA4	Zinc finger protein 318	0.027	0.983	0	1	2279	251	7.2	0
P45974-1	Ubiquitin carboxyl-terminal hydrolase 5	0.027	0.977	1	1	858	95.7	5.03	0
P01701	immunoglobulin lambda variable 1-51	0.027	0.97	7	1	117	12.2	7.03	1.6
Q7Z333	Probable helicase senataxin	0.031	0.957	0	1	2677	302.7	7.17	0
P06331	immunoglobulin heavy variable 4-34	0.031	0.957	13	1	123	13.8	9.33	0

Q9BUP0	EF-hand domain-containing protein D1	0.031	0.955	4	1	239	26.9	5.39	0
Q13427	peptidyl-prolyl cis-trans isomerase g	0.031	0.952	1	1	754	88.6	10.29	0
P68400-2	Isoform 2 of Casein kinase II subunit alpha	0.031	0.948	2	1	255	29.2	6.71	0
Q96Q15-1	Serine/threonine-protein kinase SMG1	0.032	0.943	0	1	3661	410.2	6.46	0
Q8NI27-1	THO complex subunit 2	0.032	0.937	1	1	1593	182.7	8.44	0
P02042	Hemoglobin subunit delta	0.032	0.927	6	1	147	16	8.05	1.98
Q14203	Dynactin subunit 1	0.032	0.927	1	1	1278	141.6	5.81	0
Q2NL82	Pre-rRNA-processing protein TSR1 homolog	0.034	0.901	1	1	804	91.8	7.42	0
O60942-1	mRNA-capping enzyme	0.034	0.899	2	1	597	68.5	8.13	0
Q96DC9	Ubiquitin thioesterase OTUB2	0.034	0.893	3	1	234	27.2	6.23	4.27
A6H8Y1	Transcription factor TFIIIB component B" homolog	0.034	0.89	0	1	2624	293.7	5.11	1.65
Q14571-1	Inositol 1,4,5-trisphosphate receptor	0.034	0.888	0	1	2701	307.9	6.43	2.02

	type 2								
Q9NYU2-1	UDP-glucose:glycoprotein glucosyltransferase 1	0.034	0.883	1	1	1555	177.1	5.63	0
A6NHQ2	rRNA/tRNA 2'-O-methyltransferase fibrillarin-like protein 1	0.035	0.879	3	1	334	34.8	10.35	0
P26038	Moesin	0.035	0.877	1	1	577	67.8	6.4	0
Q13367	AP-3 complex subunit beta-2	0.035	0.869	1	1	1082	119	5.59	0
P63279	SUMO-conjugating enzyme ubc9	0.035	0.868	8	1	158	18	8.66	0
Q9UMX0-1	Ubiquilin-1	0.035	0.866	3	1	589	62.5	5.11	0
P33992	DNA replication licensing factor mcm5	0.035	0.859	1	1	734	82.2	8.37	0
P28340	DNA polymerase delta catalytic subunit	0.035	0.857	1	1	1107	123.6	7.03	0
P54709	sodium/potassium-transporting ATPase subunit beta-3	0.036	0.84	5	1	279	31.5	8.35	0
Q9P0K7-1	Ankycorbin	0.036	0.84	1	1	980	110	6.21	0
P62273-1	40S ribosomal protein S29	0.036	0.838	13	1	56	6.7	10.13	1.64

Q69YQ0-1	Cytospin-A	0.035	0.826	1	1	1117	124.5	5.72	0
Q9Y450-1	HBS1-like protein	0.035	0.824	1	1	684	75.4	6.61	0
P04632	Calpain small subunit 1	0.035	0.821	6	1	268	28.3	5.2	0
P31040	Succinate dehydrogenase [ubiquinone] flavoprotein subunit, mitochondrial	0.035	0.818	3	1	664	72.6	7.39	0
Q9Y6N6	Laminin subunit gamma-3	0.035	0.815	1	1	1575	171.1	6.58	0
Q7Z2W7	Transient receptor potential cation channel subfamily M member 8	0.035	0.8	1	1	1104	127.6	7.24	0
O15347	High mobility group protein B3	0.035	0.793	4	1	200	23	8.37	0
O75886	Signal transducing adapter molecule 2	0.037	0.79	2	1	525	58.1	5.07	0
P18206	Vinculin	0.037	0.778	1	1	1134	123.7	5.66	0
Q01082-1	Spectrin beta chain, non-erythrocytic 1	0.038	0.757	0	1	2364	274.4	5.57	0
Q6NUP7-1	Serine/threonine-protein phosphatase 4 regulatory subunit 4	0.039	0.749	1	1	873	99.4	7.8	1.83

Supplementary Table 7 Reagents information in the study

Reagents	Source	Identifier
Dulbecco's modified Eagle's medium	Gibco	22320030
Fetal bovine serum	Gibco	10099133C
Trypsin-EDTA (0.25%)	Gibco	25200056
Crystal violet	Sigma-Aldrich	C0775
D-Luciferin potassium	Goldbio	LUCK-1G
TRIzol™ Reagent	Invitrogen	15596026
PrimeScript™ RT reagent Kit	TaKaRa	RR037A
TB Green® Premix Ex Taq™ II	TaKaRa	RR820A
RIPA Lysis and Extraction Buffer	Thermo Scientific	89901
Protease and phosphatase inhibitors	Thermo Scientific	78442
Pierce™ BCA Protein Assay Kit	Thermo Scientific	23225
LDS Sample Buffer (4X)	Invitrogen	NP0007
Immobilon Western Chemiluminescent HRP Substrate	Millipore	WBKLS0500
Lipofectamine™ RNAiMAX Transfection Reagent	Invitrogen	13778075
Lipofectamine™ 3000 Transfection Reagent	Invitrogen	L3000015
Polybrene	Sigma-Aldrich	TR-1003
Active Rac1 Detection Kit	CST	8815
Dynabeads™ Protein A Immunoprecipitation Kit	Invitrogen	10006D
Cell Counting Kit-8	DOJINDO	CK04
Magna MeRIP™ m6A Kit	Merck Millipore	17-10499

EZ-Magna RIP™ RNA-Binding Protein Immunoprecipitation Kit	Merck Millipore	17-701
Dual-Luciferase® Reporter Assay System Kit	Promega	E1910
SP Rabbit & Mouse HRP DAB Kit	CWBIO	CW2069
Actinomycin D	Tocris	NO.1229
Curdlan	WAKO	030-09903
Recombinant Human TNF-α	R&D Systems	210-TA
Human TNF-α Antibody	R&D Systems	MAB610
Human IL-17 Antibody	R&D Systems	AF-317
Human IL-23 Antibody	R&D Systems	AF-1716
Anti-GAPDH antibody	Abcam	ab8245
Anti-ELMO1 antibody	Abcam	ab174298
Anti-ELMO2 antibody	Abcam	ab181234
Anti-ELMO3 antibody	Abcam	ab219791
Anti-METTL3 antibody	Abcam	ab195352
Anti-METTL14 antibody	Abcam	ab220030<br>ab252562 (for CLIP)
Anti-FTO antibody	Abcam	ab126605
Anti-ALKBH5 antibody	Abcam	ab195377
Anti-WTAP antibody	Abcam	ab195380
Anti-DOCK1 antibody	Abcam	ab97325
Anti-DOCK2 antibody	Abcam	ab124838

Anti-DOCK4 antibody	Abcam	ab85723
DOCK5 Polyclonal Antibody	Thermofisher	A304-988A
Anti-DOCK8 antibody	Abcam	ab175208
Anti-CD105 antibody	Abcam	ab231774<br>ab2529
Anti-YTHDC1 antibody	Abcam	ab264375
Anti-YTHDC2 antibody	Abcam	ab220160
Anti-YTHDF1 antibody	Abcam	ab220162
Anti-YTHDF2 antibody	Abcam	ab220163
Anti-YTHDF3 antibody	Abcam	ab220161
Goat Anti-Rabbit IgG H&L (HRP)	Abcam	ab6721
Goat Anti-Mouse IgG H&L (HRP)	Abcam	ab6789
Goat Anti-Rabbit IgG H&L (Alexa Fluor® 488)	Abcam	ab150077
Goat Anti-Mouse IgG H&L (Alexa Fluor® 488)	Abcam	ab150113
Goat Anti-Rabbit IgG H&L (Alexa Fluor® 647)	Abcam	ab150079
Goat Anti-Mouse IgG H&L (Alexa Fluor® 647)	Abcam	ab150115
Rabbit IgG, monoclonal Isotype Control	Abcam	ab172730
Anti mouse-CD68 antibody	Abcam	ab125212
Anti mouse-TNF-α antibody	Abcam	ab183218
Anti mouse-CD105 antibody	Abcam	ab221675
Anti-HLA Class 1 ABC antibody	Abcam	ab70328
DAPI	ThermoFisher	D1306

HEK293T	ATCC	CRL-3216
