# Supplementary material for: TNF-α-mediated m6A modification of ELMO1 triggers directional migration of mesenchymal stem cell in ankylosing spondylitis
Source: Nat Commun. 2021 Sep 10;12:5373. doi: 10.1038/s41467-021-25710-4 (PMC8433149; doi:10.1038/s41467-021-25710-4)
Supplement: Supplementary file 3 — Reporting Summary [file 41467_2021_25710_MOESM3_ESM.pdf]

nature portfolio

Corresponding author(s): Huiyong Shen

Last updated by author(s): Jun 17, 2021

Reporting Summary

Nature Portfolio wishes to improve the reproducibility of the work that we publish. This form provides structure for consistency and transparency in reporting. For further information on Nature Portfolio policies, see our Editorial Policies and the Editorial Policy Checklist.

Statistics

For all statistical analyses, confirm that the following items are present in the figure legend, table legend, main text, or Methods section.

n/a	Confirmed	
[ ]	[x]	The exact sample size (*n*) for each experimental group/condition, given as a discrete number and unit of measurement
[ ]	[x]	A statement on whether measurements were taken from distinct samples or whether the same sample was measured repeatedly
[ ]	[x]	The statistical test(s) used AND whether they are one- or two-sided<br>*Only common tests should be described solely by name; describe more complex techniques in the Methods section.*
[x]	[ ]	A description of all covariates tested
[ ]	[x]	A description of any assumptions or corrections, such as tests of normality and adjustment for multiple comparisons
[ ]	[x]	A full description of the statistical parameters including central tendency (e.g. means) or other basic estimates (e.g. regression coefficient) AND variation (e.g. standard deviation) or associated estimates of uncertainty (e.g. confidence intervals)
[ ]	[x]	For null hypothesis testing, the test statistic (e.g. *F*, *t*, *r*) with confidence intervals, effect sizes, degrees of freedom and *P* value noted<br>*Give P values as exact values whenever suitable.*
[x]	[ ]	For Bayesian analysis, information on the choice of priors and Markov chain Monte Carlo settings
[x]	[ ]	For hierarchical and complex designs, identification of the appropriate level for tests and full reporting of outcomes
[x]	[ ]	Estimates of effect sizes (e.g. Cohen's *d*, Pearson's *r*), indicating how they were calculated

Our web collection on statistics for biologists contains articles on many of the points above.

Software and code

Policy information about availability of computer code

Data collection	SOAPnuke 1.5.2, HISAT2 2.0.4, Bowtie2 2.2.5
Data analysis	ImageJ 1.51, ImagePro Plus 6.0, BGI Dr. Tom 2.0, RadiAnt DICOM Viewer 5.0.2 software, RSEM 1.2.12, DESeq2 1.4.5, Proteome Discoverer 1.4

For manuscripts utilizing custom algorithms or software that are central to the research but not yet described in published literature, software must be made available to editors and reviewers. We strongly encourage code deposition in a community repository (e.g. GitHub). See the Nature Portfolio guidelines for submitting code & software for further information.

Data

Policy information about availability of data

All manuscripts must include a data availability statement. This statement should provide the following information, where applicable:

- Accession codes, unique identifiers, or web links for publicly available datasets
- A description of any restrictions on data availability
- For clinical datasets or third party data, please ensure that the statement adheres to our policy

The data of RNA sequencing can be obtained from the NCBI SRA database (ID SUB9027579). UniProt database (https://www.uniprot.org/) was used in the study. All other data supporting the findings of this study are available within the article and its supplementary data.

Field-specific reporting

Please select the one below that is the best fit for your research. If you are not sure, read the appropriate sections before making your selection.

- [x] Life sciences
- [ ] Behavioural & social sciences
- [ ] Ecological, evolutionary & environmental sciences

For a reference copy of the document with all sections, see nature.com/documents/nr-reporting-summary-flat.pdf

Life sciences study design

All studies must disclose on these points even when the disclosure is negative.

Sample size	No statistical methods were used to predetermine the sample size. The sample sizes were determined based on previous studies with similar experiments (Nat Commun. 2020 Jan 9;11(1):155.; J Clin Invest. 2018 Oct 1;128(10):4510-4524). For all these experiments, at least three separate experiments containing at least triplicate samples were performed, which allowed sufficient statistics to perform unpaired student t-test or ANOVA analysis, and gave p values to indicate the significance.
Data exclusions	No data was excluded.
Replication	All the results were determined based on at least three separate in vitro experiments containing at least triplicate samples.
Randomization	All samples was randomly assigned, and analyzed together in each experiment.
Blinding	Data acquisition and analysis were performed by investigators who are blinded to the groups.

Reporting for specific materials, systems and methods

We require information from authors about some types of materials, experimental systems and methods used in many studies. Here, indicate whether each material, system or method listed is relevant to your study. If you are not sure if a list item applies to your research, read the appropriate section before selecting a response.

Materials & experimental systems

n/a	Involved in the study	
☐	☒	Antibodies
☐	☒	Eukaryotic cell lines
☒	☐	Palaeontology and archaeology
☐	☒	Animals and other organisms
☐	☒	Human research participants
☒	☐	Clinical data
☒	☐	Dual use research of concern

Methods

n/a	Involved in the study	
☒	☐	ChIP-seq
☒	☐	Flow cytometry
☒	☐	MRI-based neuroimaging

Antibodies

Antibodies used	The information of all the antibodies used in the study were listed in the supplemental table 7.
Validation	The validation information of all the antibodies could be searched in the manufacturer`s websites according to the source and identifier.<br>Human TNF-α Antibody (MAB610): human, neutralization (https://www.rndsystems.com/cn/products/human-tnf-alpha-antibody-28401_mab610)<br>Human IL-17 Antibody (AF-317): human, neutralization (https://www.rndsystems.com/cn/products/human-il-17-il-17a-antibody_af-317-na)<br>Human IL-23 Antibody(AF-1716): human, neutralization (https://www.rndsystems.com/cn/products/human-il-23-p19-antibody_af1716)<br>Anti-GAPDH antibody(ab8245): human, WB (https://www.abcam.cn/gapdh-antibody-6c5-loading-control-ab8245.html)<br>Anti-ELMO1 antibody(ab174298): human, WB/IP/ICC/IF (https://www.abcam.cn/elmo1-antibody-epr12919-ab174298.html)<br>Anti-ELMO2 antibody(ab181234): human, WB (https://www.abcam.cn/elmo2-antibody-epr13567-ab181234.html)<br>Anti-ELMO3 antibody(ab219791): human, WB (https://www.abcam.cn/elmo3-antibody-ab219791.html)<br>Anti-METTL3 antibody(ab195352): human, WB (https://www.abcam.cn/mettl3-antibody-epr18810-ab195352.html)<br>Anti-METTL14 antibody(ab220030): human, WB/ICC/IF (https://www.abcam.cn/mettl14-antibody-cl4252-ab220030.html)<br>Anti-METTL14 antibody(ab252562): human, CLIP (https://www.abcam.cn/mettl14-antibody-ab252562.html)<br>Anti-FTO antibody(ab126605): human, WB (https://www.abcam.cn/fto-antibody-epr6894-ab126605.html)<br>Anti-ALKBH5 antibody(ab195377): human, WB (https://www.abcam.cn/alkbh5-antibody-epr18958-ab195377.html)<br>Anti-WTAP antibody(ab195380): human, WB (https://www.abcam.cn/wtap-antibody-epr18744-ab195380.html)<br>Anti-DOCK1 antibody(ab97325): human, WB (https://www.abcam.cn/dock180-antibody-ab97325.html)

Anti-DOCK2 antibody(ab124838): human, WB (https://www.abcam.cn/dock2-antibody-epr6647-ab124838.html)<br>Anti-DOCK4 antibody(ab85723): human, WB (https://www.abcam.cn/dock4-antibody-ab85723.html)<br>DOCK5 Polyclonal Antibody(A304-988A): human, WB (https://www.thermofisher.cn/cn/zh/antibody/product/DOCK5-Antibody-Polyclonal/A304-988A)<br>Anti-DOCK8 antibody(ab175208): human, WB (https://www.abcam.cn/dock8-antibody-epr12511-ab175208.html)<br>Anti-CD105 antibody(ab231774): human, IHC (https://www.abcam.cn/cd105-antibody-epr22811-18-ab231774.html)<br>Anti-CD105 antibody(ab2529): human, ICC/IF (https://www.abcam.cn/cd105-antibody-mem-226-ab2529.html)<br>Anti-YTHDC1 antibody(ab264375): human, WB/IP (https://www.abcam.cn/ythdc1-antibody-ab264375.html)<br>Anti-YTHDC2 antibody(ab220160): human, WB/IP (https://www.abcam.cn/ythdc2-antibody-epr21820-49-ab220160.html)<br>Anti-YTHDF1 antibody(ab220162): human, WB/IP (https://www.abcam.cn/ythdf1-antibody-epr22349-41-ab220162.html)<br>Anti-YTHDF2 antibody(ab220163): human, WB/IP (https://www.abcam.cn/ythdf1-antibody-epr22349-41-ab220163.html)<br>Anti-YTHDF3 antibody(ab220161): human, WB/IP (https://www.abcam.cn/ythdf1-antibody-epr22349-41-ab220161.html)<br>Anti mouse-CD68 antibody(ab125212): mouse, IP(https://www.abcam.cn/cd68-antibody-ab125212.html)<br>Anti mouse-TNF antibody(ab183218): mouse, IP(https://www.abcam.cn/tnf-alpha-antibody-epr19147-ab183218.html)<br>Anti mouse-CD105 antibody(ab221675): mouse, IP(https://www.abcam.cn/cd105-antibody-epr21846-ab221675.html)<br>Anti-HLA Class 1 ABC antibody(ab70328): human, IP (https://www.abcam.cn/hla-class-1-abc-antibody-emr8-5-ab70328.html)

Eukaryotic cell lines

Policy information about cell lines

Cell line source(s)	HEK293T cells
Authentication	HEK293T cells were purchased and authenticated from ATCC.
Mycoplasma contamination	HEK293T cells were monthly tested for Mycoplasma contamination. All the tested were negative for Mycoplasma.
Commonly misidentified lines (See ICLAC register)	No commonly misidentified cell line was used in this study.

Animals and other organisms

Policy information about studies involving animals; ARRIVE guidelines recommended for reporting animal research

Laboratory animals	Male SKG mice at 4 weeks of age were purchased from CLEA Japan, Inc.. Male SKG mice at 8 weeks of age were used for experiments. Female BALB/c-nu/nu mice at 8 weeks of age were purchased from Laboratory Animal Center of Sun Yat-Sen University and used for experiments. Mice were housed at the Laboratory Animal Center of Sun Yat-Sen University under specific pathogen-free conditions, with a 12-hour light/dark cycle in a temperature and humidity-controlled room (22±2°C; 60%) with free access to water and food.
Wild animals	No wild animals were used in this study.
Field-collected samples	No field-collected samples were used in this study.
Ethics oversight	The experiments on mice were approved by the Institutional Animal Care and Use Committee of Sun Yat-Sen University, Guangzhou, China.

Note that full information on the approval of the study protocol must also be provided in the manuscript.

Human research participants

Policy information about studies involving human research participants

Population characteristics	The information about the study subjects was shown in Supplemental Table 1 and 4.
Recruitment	A total of 15 patients with AS and 15 healthy controls were randomly recruited for this study voluntarily from the Department of Orthopedics, The Eighth Affiliated Hospital, Sun Yat-sen University. No potential self-selection bias or other biases was present in this study.
Ethics oversight	This study was approved by the Ethics Committee of the Eighth Affiliated Hospital, Sun Yat-Sen University, Guangzhou, China.

Note that full information on the approval of the study protocol must also be provided in the manuscript.
